# Light‐Driven Competitive Selection in a Protein‐Catalyzed Dissipative Peptide Replication

**DOI:** 10.1002/anie.202518911

**Published:** 2026-02-01

**Authors:** Éva Bartus, Edit Wéber, Attila Tököli, Ferenc Bogár, Momen R. F. Mohamed, Gábor Kecskeméti, Zoltán Szabó, Zoltán Kele, András Perczel, Márton Gadanecz, Zoltán Orgován, György M. Keserű, Tamás A. Martinek

**Affiliations:** ^1^ Department of Medical Chemistry University of Szeged Szeged Hungary; ^2^ HUN‐REN‐SZTE Biomimetic Systems Research Group University of Szeged Szeged Hungary; ^3^ Department of Biochemistry University of Cambridge Cambridge UK; ^4^ Department of Medicinal Chemistry Faculty of Pharmacy Minia University Minia Egypt; ^5^ Laboratory of Structural Chemistry and Biology Institute of Chemistry Eötvös Loránd University Budapest Hungary; ^6^ HUN‐REN‐ELTE Protein Modeling Research Group, Institute of Chemistry Eötvös Loránd University Budapest Hungary; ^7^ HUN‐REN Research Centre For Natural Sciences Drug Innovation Centre and National Drug Discovery and Development Laboratory Budapest Hungary; ^8^ Hevesy György PhD School of Chemistry Institute of Chemistry Eötvös Loránd University Budapest Hungary; ^9^ Medicinal Chemistry Research Group HUN‐REN Research Centre For Natural Sciences Budapest Hungary; ^10^ Department of Organic Chemistry and Technology Faculty of Chemical Technology and Biotechnology Budapest University of Technology and Economics Budapest Hungary

**Keywords:** catalyzed replication, competitive selection, dissipative, foldamer, light‐driven disulfide rearrangement

## Abstract

Theoretical models of prebiotic evolution propose that catalytic modulation can influence autocatalytic replication cycles. We hypothesized that a protein catalyst could create a favorable environment for proximity‐controlled, dissipative replication of primitive peptides. This setup, in principle, allows competitive selection by making the catalyst a limited resource. Here, we show that a structurally flexible and promiscuous protein, calmodulin, catalyzes UVA light‐driven replication in a helical foldamer‐based replicator system. The protein accelerates both non‐autocatalytic synthesis and autocatalytic replication, with autocatalysis being the dominant pathway. The system demonstrates light‐intensity‐dependent competitive selection, where replicators compete for scarce binding sites on the catalyst. This mechanism enables efficient selection even within a large replicator pool. Our results provide evidence that the specific catalyst–replicator interactions envisioned in Eigen's hypercycle model can be approximated under dissipative conditions in catalyzed primitive replicator systems.

## Graphical abstract

A structurally flexible protein catalyzes replication within a dissipative reaction network driven by UVA‐induced disulfide rearrangements. The protein accelerates templated autocatalytic cycles with replication emerging as the dominant pathway. Competition among replicators for limited catalyst binding sites enables light intensity‐dependent competitive selection.

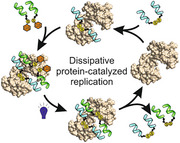

## 1 Introduction

Darwinian‐like evolution at the molecular level is expected to emerge from the functional integration of replication, metabolism, and compartmentalization in out‐of‐equilibrium systems [1]. Chemical evolution can be viewed as an intermediate stage on the pathway toward full biological evolution. It is characterized by three key requirements [2]: (i) replication that transmits the properties of the parent, (ii) a physicochemical selection mechanism that allows fitter individuals to survive and/or reproduce more efficiently than less fit ones, and (iii) a mechanism that generates diversity within the population. Nonequilibrium molecular systems capable of undergoing chemical evolution combine templated replication, kinetically asymmetric replicator breakdown, and a diversity‐generating random replicator synthesis process [1, 3, 4]. Together, these competing reactions enable the transfer of structural information and selection without relying on a biologically evolvable information‐coding macromolecule such as DNA. Effective selection is entropically favored only far from equilibrium, particularly in large replicator populations [5]. Consequently, chemical evolution must take place in dissipative systems that use chemical [6, 7, 8, 9, 10] or light energy [11, 12, 13, 14] to sustain off‐equilibrium replicator populations. However, primitive replicators typically exhibit weak templating interactions, which limit their capacity for selective amplification. Scarcity of resources can enhance selection efficiency through competitive selection or, in extreme cases, competitive exclusion [15, 16, 17, 18, 19]. Competition for chemical precursors in parallel replication pathways provides a clear example of this mechanism. Nonetheless, additional selection routes can also be envisaged in the broader context of chemical evolution.

**FIGURE 1:**
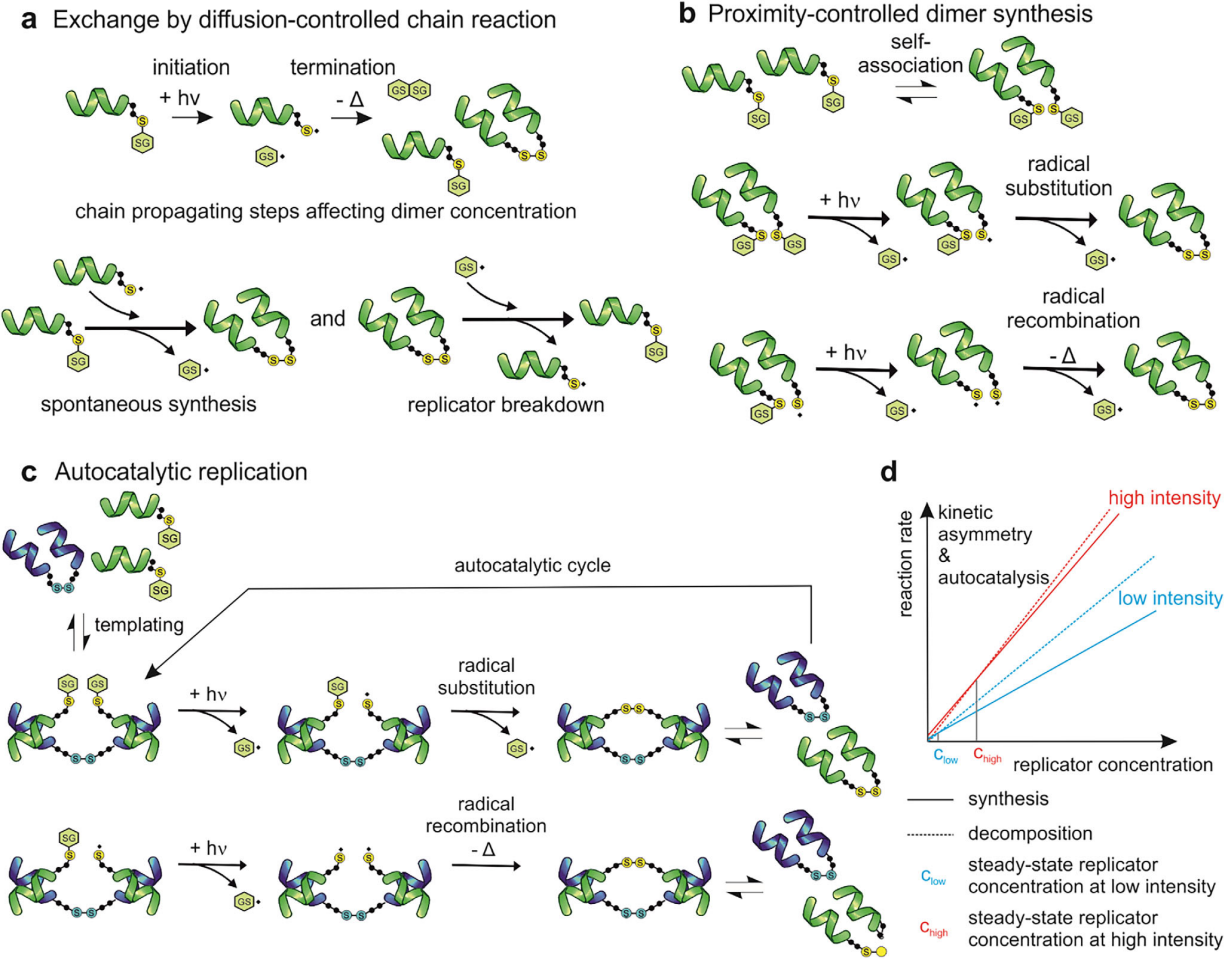
Schematic representation of the dynamic model of the chemically evolvable foldameric replicator system. Foldamers (helices) are functionalized with a terminal Cys. Terminal sulfurs and thiyl radicals are depicted with yellow circles. GS and SG in hexagons indicate glutathione. (a) Spontaneous synthesis and replicator breakdown through diffusion‐controlled radical chain reaction. (b) Spontaneous synthesis via foldamer association pre‐equilibrium and subsequent proximity‐controlled radical substitution or concerted metathesis. (c) Templated autocatalysis under proximity control facilitates the proximity‐controlled radical substitution or concerted metathesis. (d) Schematic representation of the effects of kinetic asymmetry and autocatalysis on the light‐induced steady‐state replicator concentrations. Lines represent the replicator concentration‐dependent reaction rates of replicator synthesis and breakdown. Because of the kinetic asymmetry between the replication and the breakdown, the slopes of the curves depend on light intensity in a different order. The steady state replicator concentration is reached at the intercept of the rate curves. Thus, linear light intensity increase induces a hyperbolic increase of the replicator concentration [4].

Experimental models that display features of chemical evolution are crucial for understanding self‐organization processes that give rise to life‐like behavior at the molecular scale. Previously, we constructed a chemical replicator system from primitive peptidic foldamer components that preferentially adopt helical conformations [20]. This autocatalytic network was driven into off‐equilibrium states in a dissipative manner by a UVA‐light‐induced disulfide rearrangement [4]. In this setup, a diffusion‐controlled radical chain reaction did not contribute to replicator formation (Figure 1a), while replicator breakdown into precursors proceeded through this pathway. Sequence‐dependent self‐association of the foldamers provided proximity control, enabling their spontaneous, non‐autocatalytic formation (Figure 1b). Templating then introduced auto‐ and cross‐catalysis, promoting replicator formation via proximity‐controlled radical substitution and recombination (Figure 1c). The kinetically asymmetric replication and degradation pathways produced a hyperbolic increase in replicator concentration at elevated light intensity (Figure 1d). These competing dissipative processes generated sequence‐dependent selection within an adaptive dynamic kinetic stability regime that is characteristic of chemical evolution.

In principle, a templated replication cycle can be further accelerated by an additional molecular entity that creates a favorable environment for the templating step. This catalytic function can occur within a complex involving the catalyst, the replicator, and its precursors. Eigen's theoretical hypercycle model introduced the idea of catalyzed autocatalytic replication [21], which underpins the coupling of primitive replication cycles through interactions that are specific to individual replicators. This approach differs from simply passing templates between subsystems [22]. In this way, a closed loop of specifically catalyzed replication cycles forms what Eigen termed a hypercycle. However, in a typical random autocatalytic set, a catalyst is unlikely to be entirely specific to just one replication cycle. Under these more general conditions, replicators compete for the catalyst, paving the way for a potentially powerful competitive selection mechanism. This catalyst‐driven competitive selection is expected to yield high efficiency, even within a large pool of replicators. Moreover, it can operate in adaptive dissipative dynamics without depleting precursors—a key necessity for chemical evolution.

**FIGURE 2:**
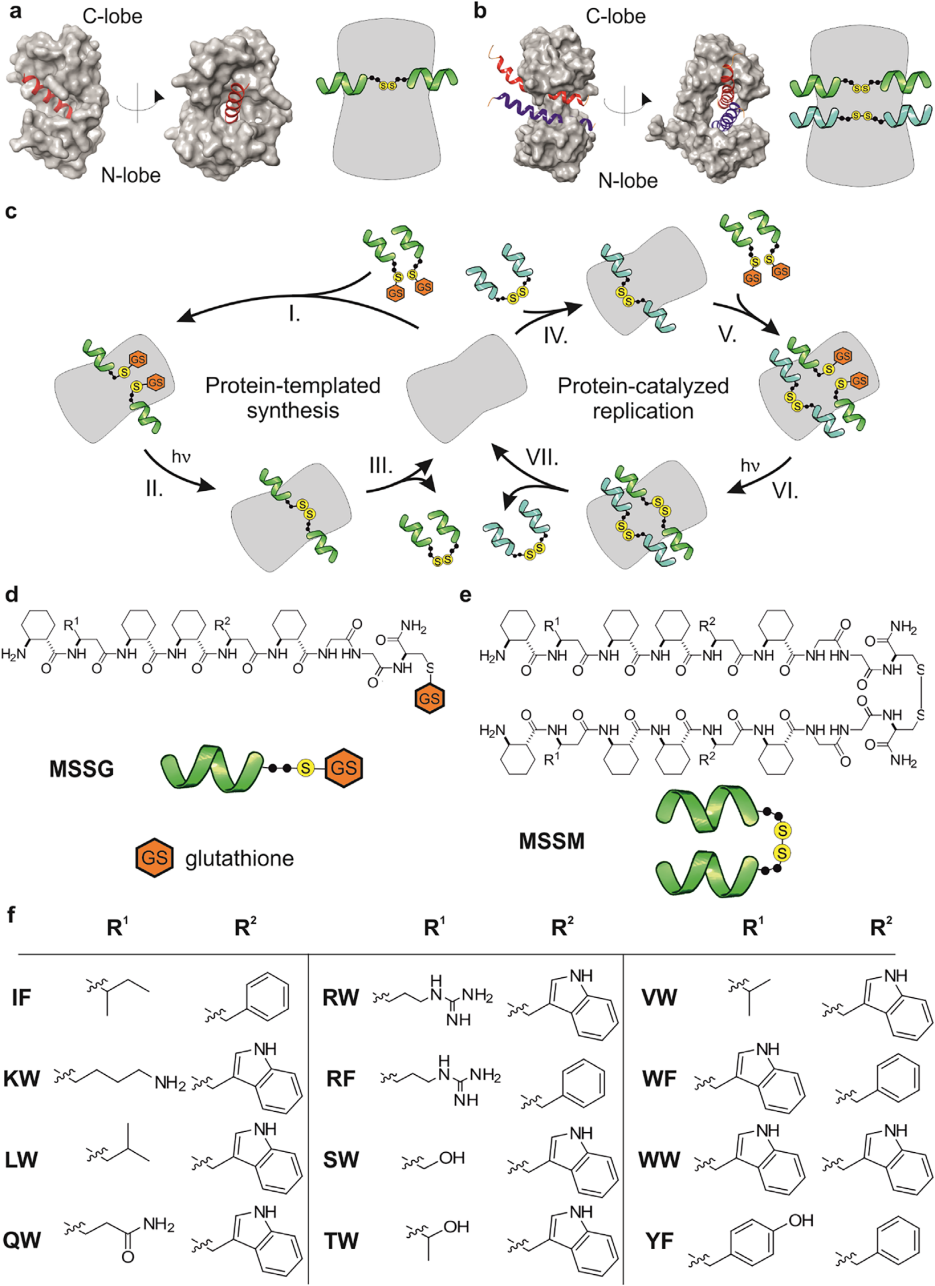
Protein‐catalyzed dissipative replicator system. (a) Structure of CaM bound to a single helical sequence, which is the hydrophobic IQ domain of the cardiac Ca_V_1.2 calcium channel (PDB ID: 2F3Y) [29]. Schematic representation of CaM bound to a replicator (right). (b) Structure of an unusual CaM‐peptide complex: helices hA and hB of K_V_7.2 K+ channel (KCNQ2) simultaneously bound to CaM (PDB ID: 6FEH) [28]. Schematic representation of the supposed structure of CaM bound to two replicators simultaneously, resembling the transition state of the catalyzed replication (right). (c) General scheme for coupling the formation‐recombination cycle of the UVA‐light‐induced thiyl radicals to the non‐autocatalytic templated synthesis (left) and the protein‐catalyzed replication (right). The following reaction steps were hypothesized (see Figure S1 for details): I. binding of precursors to the protein; II. protein‐catalyzed synthesis of a dimer via radical substitution or recombination; III. dissociation of the dimer from the binding site; IV. formation of a protein‐replicator complex; V. precursor binding to the protein‐replicator complex, VI. protein‐catalyzed radical substitution or recombination in the replication step, VII. dissociation of the replicator(s) from the protein. (d) Schematic representation and the corresponding general sequences of the foldameric building blocks (MSSG: glutathione‐protected monomers), glutathione: γ‐L‐glutamyl‐L‐cysteinyl‐glycine, and (e) dimeric replicator (MSSM). (f) Side chain combinations in the highlighted positions *R* ^1^ and *R* ^2^. β^3^‐amino acids with proteinogenic side chains were incorporated in the marked combinations, resulting in 12 different β‐peptidic sequences: IF, KW, LW, QW, RW, RF, SW, TW, VW, WF, WW, and YF. One‐letter codes correspond to the side chains in the standard α‐amino notation. The initial library was hereinafter referred to as Mix12.

In this study, the goal was to identify a suitable catalyst that influences dissipative replication cycles and to investigate the potential for catalyst‐mediated competitive selection. To achieve this, a protein partner was envisioned that could promiscuously bind both the foldameric replicator templates and their precursors, thereby boosting the replication rate. Calmodulin (CaM) was selected as the catalyst due to its well‐documented binding patterns and structural adaptability [23, 24, 25]. This 148‐residue calcium‐binding protein undergoes substantial conformational shifts when interacting with partners. Its remarkable flexibility enables promiscuous binding to targets of diverse sizes and sequences. In prior work, interactions between CaM and a variety of short helical foldamer sequences were examined [26]. These foldamers bind to the protein with low micromolar affinities, mirroring the patterns seen in traditional CaM‐ligand complexes [23, 24] (Figure 2a). Due to this flexibility, CaM can also accommodate two helix segments at once between its hydrophobic patches [27, 28] (Figure 2b). Building on these observations, it was hypothesized that CaM could bind peptide‐templated precursors in the rate‐determining transition state complex, thereby enhancing sequence‐dependent replication rates via a proximity‐controlled pathway (Figure 2c). It was further hypothesized that CaM's structural plasticity might enable protein‐templated synthesis without relying on an autocatalytic cycle, potentially generating initial replicator sequences to launch their production. That said, it was not possible to exclude beforehand the chance that precursor or replicator interactions with the protein might stabilize unproductive complexes, thereby inhibiting both non‐autocatalytic formation and the replication cycles.

Here, we show that a protein can serve a dual role in a dissipative replicator system: it catalyzes the templated autocatalytic cycle of the replicators while also introducing a protein‐templated synthesis pathway that provides an effective initiating mechanism for replicator production. Adjusting light intensity and catalyst concentration highlighted competition for the protein's catalytic environment, resulting in an efficient competitive selection mechanism.

## 2 Results and Discussion

### 2.1 Adaptation of the Dissipative Replicator System to the Presence of the Protein

The experiments were built upon a recently published foldameric system that exhibits features of chemical evolution, including UVA light‐driven dissipative primitive replication and a kinetically asymmetric replicator breakdown mechanism [4]. This replicator system leverages its off‐equilibrium adaptive behavior, dynamically adjusting steady‐state replicator concentrations in response to varying light energy input and seeding. The competing replication and breakdown reactions are driven by photochemical disulfide rearrangement at the C‐terminal Cys residues (Figure 1, Scheme S1). The setup features 12 distinct helical foldamers bearing proteinogenic side chains at variable positions (**Mix12**, Figure 2d–f). Sequence‐dependent templating in the auto‐ and cross‐catalytic replication is controlled by weak nonbonding interactions within the peptidic foldamer network. Previous findings showed that templated auto/cross‐catalysis, combined with asymmetric breakdown, triggers a hyperbolic (switch‐like) rise in replicator concentration as illuminating light intensity increases linearly [4].

**FIGURE 3:**
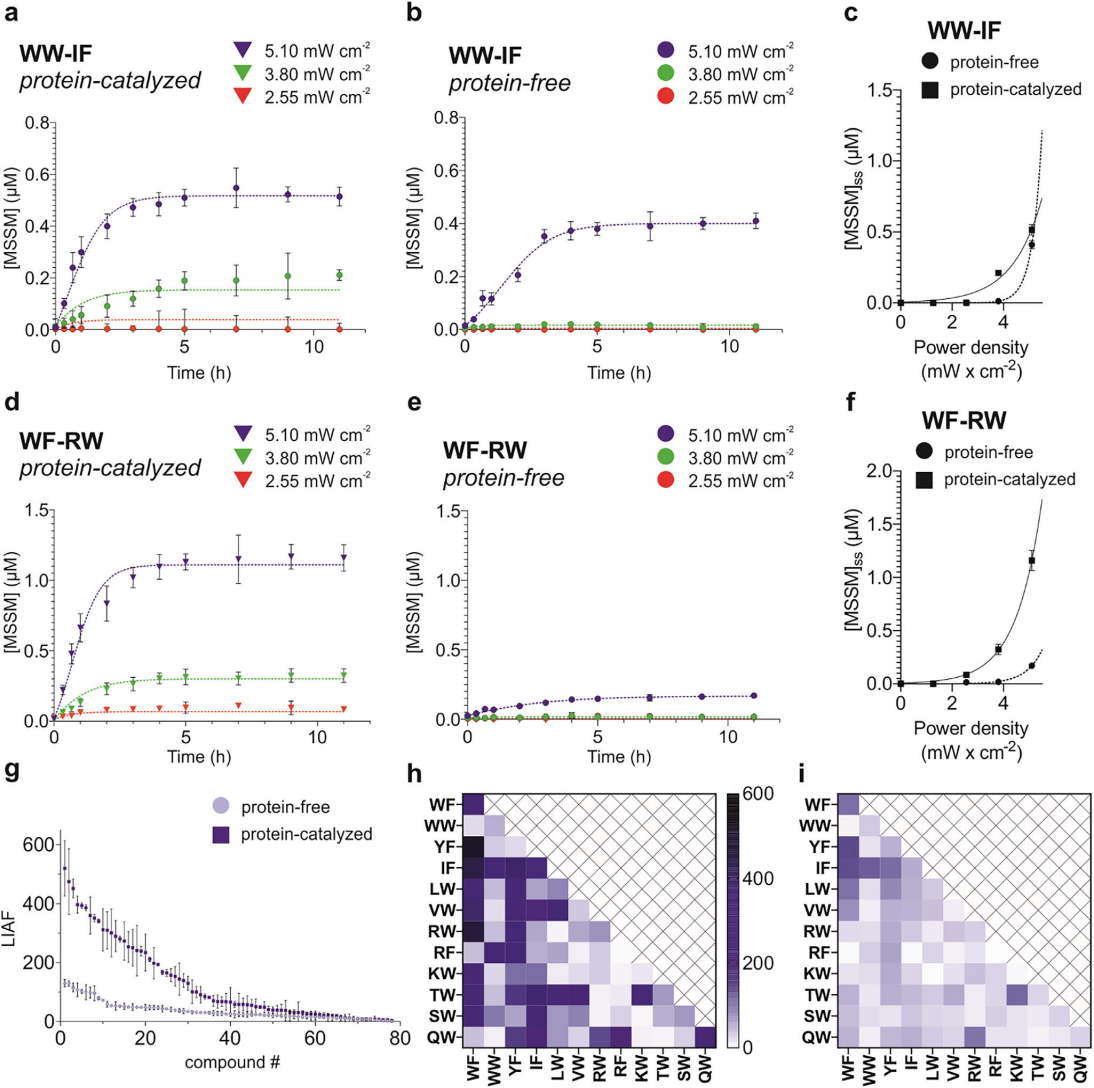
Protein‐catalyzed light intensity‐dependent dissipative replication. Time‐ and light intensity‐dependent concentrations of representative replicators **WW‐IF** and **WF‐RW** in the protein‐templated system (a) and (d) and in the protein‐free system (b) and (e). Irradiation was performed at light intensities: 2.55 mW cm^−2^ (red, 50%), 3.80 mW cm^−2^ (green, 75%), and 5.10 mW cm^−2^ (blue, 100%). Dashed curves display the fitted dynamic simulations based on the dissipative dynamic model [4] (Figure S3). Light intensity‐dependent steady‐state concentrations for the representative sequences (c) and (f) in the protein‐free (circle) and the protein‐catalyzed (square) systems. The fitted curve represents the simulated hyperbolic relationship between steady‐state concentrations and illumination power density. (g) LIAF obtained upon increasing power density from 2.55 to 5.10 mW cm^−2^ for the protein‐free (light blue circle) and protein‐containing (dark blue square) samples. All data are expressed as mean ± SD (*n* = 3). Heatmap representations of the LIAFs obtained upon increasing power density from 2.55 to 5.10 mW cm^−2^ for the protein‐containing (h) and the protein‐free (i) systems.

Before assessing CaM's impact on the replicator system, UVA light‐induced protein degradation was ruled out over the measurement duration (Figure S2, Table S1). Upon illuminating CaM‐containing samples, steady‐state concentrations rose sharply for most dimers (Figures 3a,d, and S3) compared to the protein‐free controls (Figures 3b,e, and S3). Hyperbolic growth in steady‐state replicator populations emerged with linear increases in light intensity (Figure 3c,f), confirming templated autocatalytic processes at work. Without autocatalysis, steady‐state concentrations would follow a shallower power‐law dependence on light intensity (Figure S4).

Replicator diversity was quantified using the light‐induced amplification factor (LIAF), defined as the ratio of replicator concentration at 100% light intensity (5.1 mW cm^−2^) to that at 50% (2.55 mW cm^−2^). LIAF values for the top ten dimers ranged from 520 to 290 (Figure 3g, Table S2), far exceeding the protein‐free system's range of 130 to 73. System selectivity is evident in the ratio of best‐to‐worst LIAFs: 85.0 without protein and 197.7 with CaM catalysis. These figures reflect enhanced discrimination between the compounds in the presence of the protein. Sequence‐dependent selection patterns were similar for the protein‐catalyzed and protein‐free systems (Figure 3h,i). These observations confirm that CaM functions as a catalyst rather than an inhibitor in this dissipative setup and underscore the system's sensitive adaptation to the protein's catalytic influence.

### 2.2 Catalytic Effect of the Protein on the Autocatalysis

**FIGURE 4:**
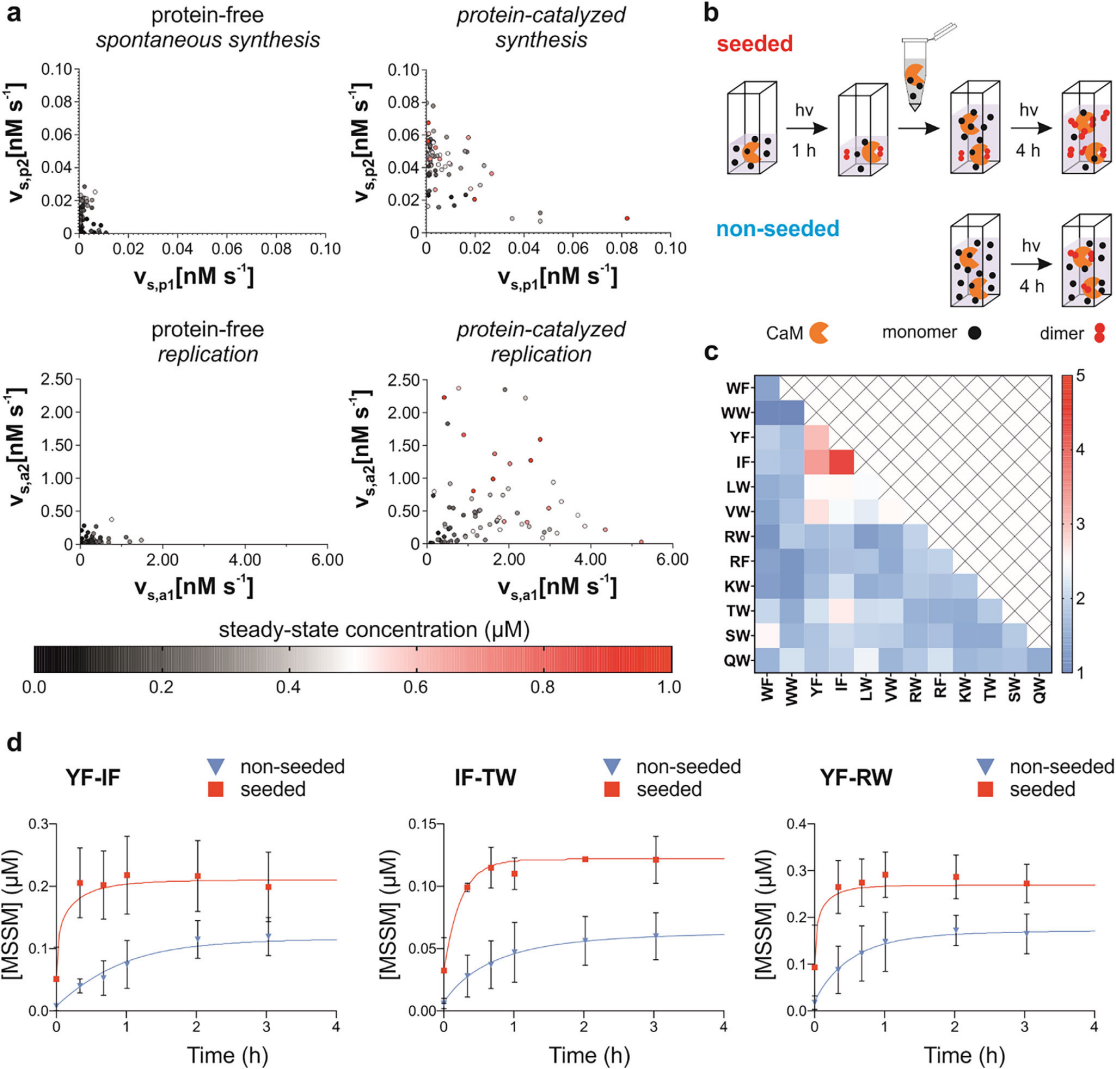
Catalytic effects of the CaM protein and influence of replicator seeding. (a) Steady‐state reaction rates of the following replicator synthesis pathways: non‐autocatalytic radical substitution (v_s,p1_), non‐autocatalytic radical recombination (v_s,p2_), autocatalytic radical substitution (v_s,a1_), autocatalytic radical recombination (v_s,a2_). Each dot represents a unique replicator in the system. Color coding indicates the steady‐state concentration of the replicator. Protein‐free and protein‐catalyzed systems are displayed in separate panels. (b) Schematic representation of the seeding experiment: a mixture of precursors and replicators was prepared in an appropriate ratio (Table S5) by irradiating a monomeric mixture **Mix12** in the presence of CaM at 4.2 mW cm^−2^ for 1 h, and this sample was used for seeding a freshly prepared monomeric mixture. As a non‐seeded reference, a reaction was started in parallel without the seeding mixture, and both samples were irradiated at 4.2 mW cm^−2^ for a further 4 h. (c) Heatmap representation of the sequence‐dependent initial rate increase upon seeding expressed as v_seeded_/v_non‐seeded_ at 40 min (Table S6). (d) Time evolution of the concentrations for representative replicators **YF‐IF**, **IF‐TW**, **YF‐RW** in the seeded (red square) and in the non‐seeded (blue triangle) samples, fitted curves are depicted with matching colors. All data are expressed as mean ± SD (*n *= 3 independent repeats). Experimental data of the full set of replicators are given in Figures S5 and S6.

Next, the catalytic effects of CaM were quantified by fitting a previously published kinetic model of chemical evolution [4] to the experimental data (Supporting Information). This model incorporates proximity‐controlled non‐autocatalytic and templated autocatalytic pathways for dimer synthesis, including radical substitution and recombination steps. It also accounts for replicator decomposition via the radical chain reaction mechanism [4]. Reaction rates and apparent rate constants were calculated for each replicator (Figure 4a, Tables S3 and S4). Protein effects are reflected in the adjusted rate constants. Comparison between protein‐free and CaM‐catalyzed systems showed acceleration of the proximity‐controlled radical recombination in non‐autocatalytic dimer synthesis (v_s,p2_) for most replicators, creating an efficient initiation mechanism. In some cases, proximity‐controlled non‐autocatalytic radical substitution (v_s,p1_) rates also increased. Meanwhile, CaM boosted templated autocatalytic synthesis rates (v_s,a1_ and v_s,a2_) for many sequences—more so than for non‐autocatalytic routes—with sequence‐dependent effects on both recombination and substitution pathways. These results strongly indicate that CaM catalyzes dimer‐templated autocatalytic replication. Notably, replication rates exceeded non‐autocatalytic synthesis by an order of magnitude, confirming the protein‐catalyzed autocatalytic pathway as the dominant replicator formation route (Figure 4a).

To verify autocatalysis, the CaM‐catalyzed system was seeded with a mixture of pre‐synthesized replicators. This seeding mix was prepared by irradiating the protein‐containing foldamer library with UV light for 1 h, yielding a dimer‐rich population that CaM effectively replicates. After quantitatively analyzing the mixture, we set the total seeding dimer concentration at 6 µM (Table S5). The monomer and protein levels were matched to the non‐seeded library (Figure 4b). This setup mimicked an early evolutionary stage replicator distribution. A non‐seeded reference was irradiated alongside for 4 h, with regular analysis. Kinetic differences between seeded and non‐seeded CaM samples were assessed by model fitting and Monte Carlo simulations (*n* = 1000 per replicator) for 95% confidence intervals. Of 78 replicators, 42 (53.8%) showed significant nonoverlapping intervals (Figure S6), 30 (38.5%) had partial overlap, and 6 (7.7%) fully overlapped, indicating no difference. Seeding markedly raised initial synthesis rates for several dimers (Figures 4c,d, and S5), confirming autocatalysis in the CaM‐foldamer system.

The initial **Mix12** was also seeded with a light‐insensitive thioether dimer derivative (**WF‐S‐RW**), a strong CaM binder [26]. This had a negligible seeding effect on the protein‐free system (Figure S7) [4], though its disulfide parent replicated well under CaM catalysis. Applied at equimolar CaM concentration under 4.20 mW cm^−2^ light, **WF‐S‐RW** inhibited dimer formation across nearly all cases (Figure S8). Reducing it to 0.5 µM eliminated inhibition but yielded no seeding. This inhibition likely stems from the displacement of monomer units or the failure to form productive transition states with CaM‐bound **WF‐S‐RW**‐templated monomers.

### 2.3 Structural Background of the Catalyzed Autocatalysis

**FIGURE 5:**
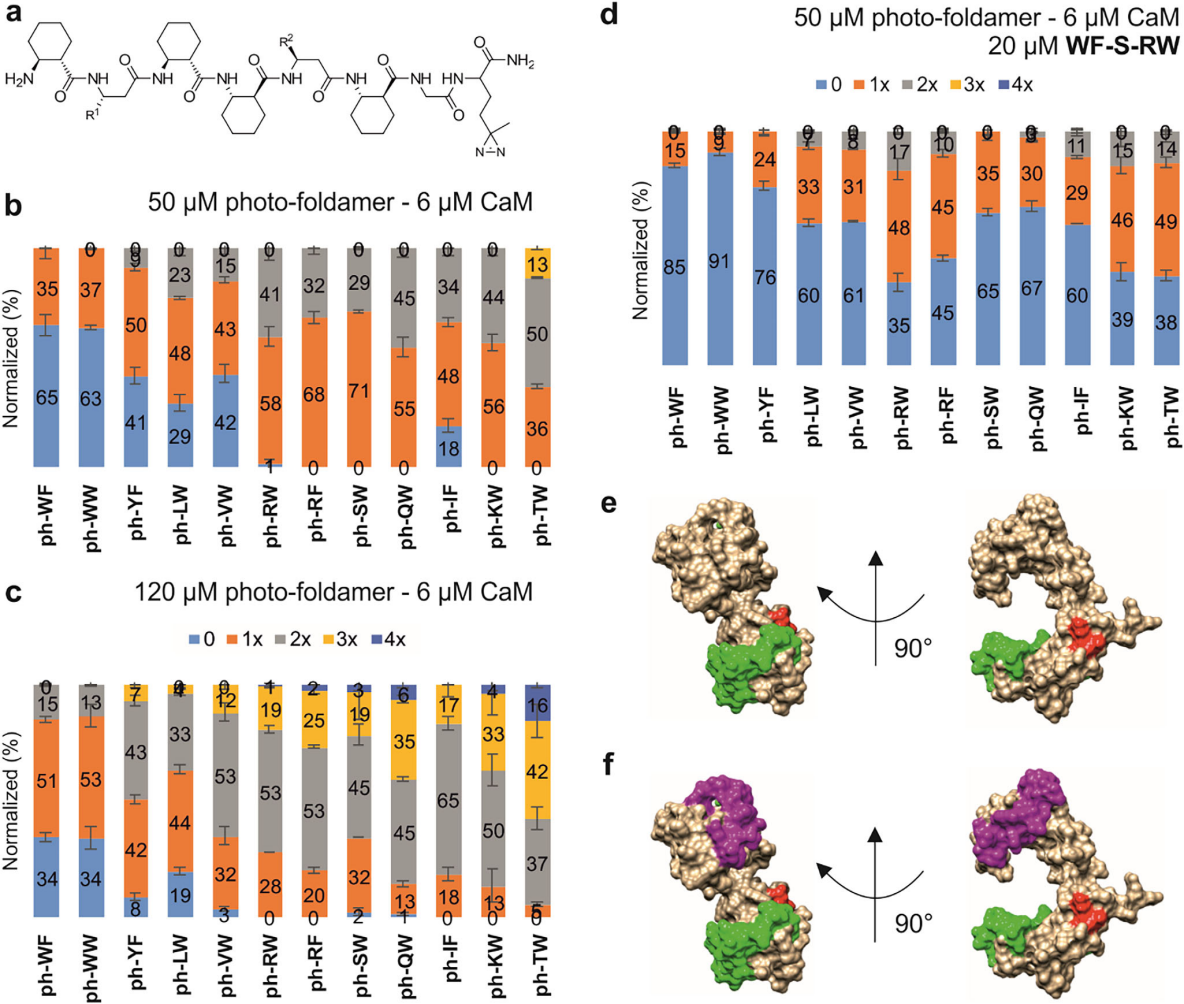
Localization of the precursor binding sites on CaM with photo‐foldamer screening. (a) General sequence of the foldamers with the photoreactive diazirine residue attached to the C‐terminus. β^3^‐amino acids with proteinogenic side chains at positions *R* ^1^ and *R* ^2^ correspond to the monomeric precursors given in Figure 2f. For the UV‐induced metathesis reaction, the following side chain combinations were used: **ph‐IF**, **ph‐KW**, **ph‐LW**, **ph‐QW**, **ph‐RW**, **ph‐RF**, **ph‐SW**, **ph‐TW**, **ph‐VW**, **ph‐WF**, **ph‐WW,** and **ph‐YF**. One‐letter codes correspond to the side chains in the standard α‐amino notation. For the concentration‐dependent crosslinking reactions, CaM was dissolved in 20 mM HEPES, 150 mM NaCl, and 2 mM CaCl_2_ at a concentration of 6 µM, and photo‐foldamers were applied at two different concentrations: 50 µM (b) and 120 µM (c). Crosslinked foldamer‐CaM complexes with different stoichiometries are indicated with different colors: CaM:foldamer 1:1 (orange), CaM:foldamer 1:2 (gray), CaM:foldamer 1:3 (yellow), and CaM:foldamer 1:4 (dark blue). (d) Crosslinking experiment in the presence of a replicator mimetic (**WF‐S‐RW**) at 20 µM, CaM at 6 µM, and photo‐foldamer at 50 µM. All data are expressed as mean ± SD (*n *= 3). e) Front and side views of CaM (PDB ID: 6FEH) with the highlighted residues 15–16 (red) and residues 39–67 (green). f) Front and side views of CaM (PDB ID: 6FEH) with the photo‐foldamer anchor regions in the presence of the replicator mimetic (**WF‐S‐RW**).

The data above confirm that templated autocatalytic replicator synthesis proceeds in a protein‐catalyzed manner. This outcome supports the initial hypothesis that CaM can accommodate the transition state for templated dimer formation, with two replicators bound simultaneously. To gain deeper insight into this mechanism, we detected the precise localization of the contact sites for the catalytically relevant transient interactions. Recently, we detected weak protein binding and mapped using photoaffinity tags on foldamer segments [30]. To assess CaM's capacity to bind a foldameric replicator while simultaneously templating its monomeric subunits, C‐terminal Gly‐Cys residues of **Mix12** monomers were substituted with diazirine‐based photoaffinity tags (photo‐foldamers, Figure 5a). The cross‐linking efficiency to CaM was measured at two concentrations (Figure 5b,c). At 6 µM CaM and 50 µM photo‐foldamer, CaM reliably bound two monomeric segments at once. Raising the photo‐foldamer excess to twenty‐fold produced sequence‐dependent 3:1 monomer‐CaM complexes.

Binding sites were pinpointed via LC‐MS/MS after enzymatic digestion of the cross‐linked complexes. Two anchor sites emerged on CaM's N‐terminal lobe (Figure 5e: residues 15–16 in red, 39–67 in green; Table S7). Despite CaM's high level of symmetry, the C‐terminal lobe showed no tagging. Cross‐linking persisted even with 20 µM dimeric binder **WF‐S‐RW** present (Figure 5d), detectable for all studied monomers. Multiple labelling events were found for more than half of the foldamer derivatives (**ph‐LW, ph‐VW, ph‐RW, ph‐RF, ph‐IF, ph‐KW, ph‐TW**). This proves that **WF‐S‐RW** does not displace monomers, allowing simultaneous templating of dimer and precursor monomer interactions. Proteomic analysis with **WF‐S‐RW** revealed additional sites beyond those without a dimer. A new surface patch appeared on the C‐terminal lobe for binding the following sequences: **ph‐RW**, **ph‐RF**, **ph‐TW**, **ph‐LW**, **ph‐YF** (Figure 5f, residues 117–136 labelled with magenta, Table S7). Given **WF‐S‐RW**’s low nanomolar affinity to CaM, its binding site should be saturated in this experimental setup. These findings highlight CaM's ability to recruit extra monomers even when occupied by a high‐affinity dimer, providing structural evidence for protein‐catalyzed autocatalytic replication. Monomer displacement can be ruled out as the reason for **WF‐S‐RW**’s lack of seeding. We speculate that the thioether linkage fails to stabilize the templated dimer transition state within CaM.

CaM's remarkable promiscuity and structural flexibility likely underpin its catalytic success. To test generalizability, replication was attempted with a challenging protein K−Ras G12D, featuring targetable surface patches for foldamers. The foldameric library was previously pre‐screened specifically for K‐Ras [30] binding and synthesized as glutathione‐protected monomers (Table S8). The K‐Ras‐optimized foldamer library showed minimal overlap with the CaM library because of differences in their protein surfaces. Dimer formation via UV‐induced disulfide exchange was compared with and without K‐Ras. In the protein‐free sample, K‐Ras‐selected monomers dimerized far less than the CaM library (Figure S9), with concentrations near HPLC‐MS detection limits even at maximum light intensity. The decreased reactivity precluded light‐intensity dependence studies or distinction between spontaneous synthesis and replication. These findings show that the composition of the foldamer library is crucial for a sufficient baseline replication rate. Adding K‐Ras raised steady‐state dimer levels for specific sequences (**QR‐QF**, **QW‐LR**, **QR‐QW**), suggesting potential protein catalysis.

### 2.4 Dissipative Adaptation Leads to Energy Influx‐Dependent Competitive Selection

Our initial hypothesis was that replicators would compete for protein‐binding sites as increasing light intensity raises their total concentration in the protein‐catalyzed system. Precursor depletion is minimal in this setup, so any limitation should arise primarily from access to the catalytic protein rather than from a lack of building blocks. If such competition occurs, the protein‐induced amplification factors (PIAF) should not increase monotonically with light intensity for every replicator. PIAF is defined as the ratio of a given replicator's concentration in the protein‐catalyzed system to its concentration in the protein‐free system. Once catalytic binding sites on the protein become limiting, the fittest replicators are expected to displace weaker binders, reducing their formation rates, while effective breakdown further suppresses the concentrations of the weaker species.

**FIGURE 6:**
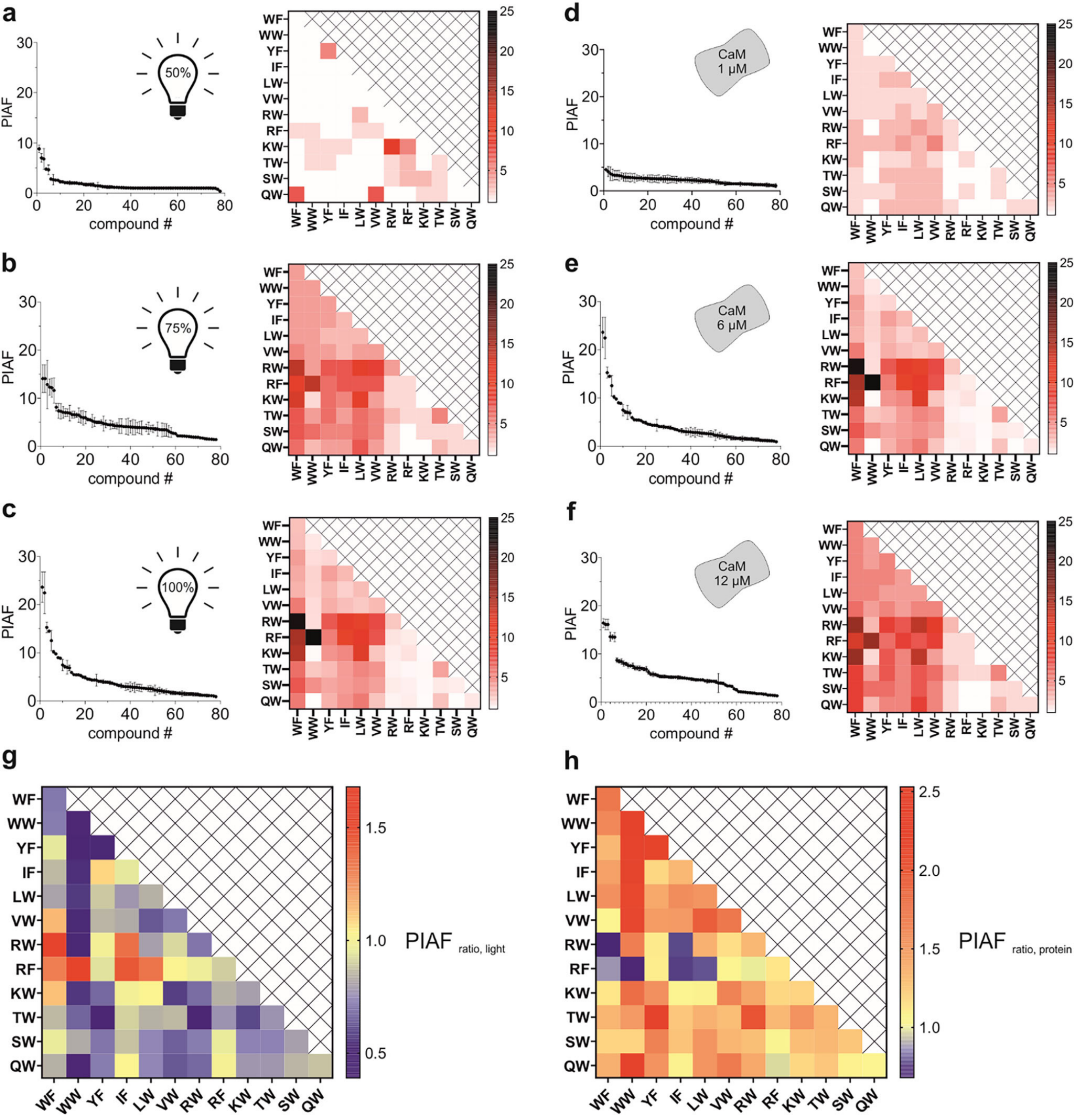
PIAFs and light‐driven competitive selection. PIAFs were calculated for each replicator as the ratios of the steady‐state concentrations obtained in the protein‐catalyzed and the protein‐free systems. PIAF values are visualized as amplification profiles and heatmaps, ordered in descending order (Tables S9 and S10). Data were measured at CaM concentration of 6 µM and light intensities of 2.55 mW cm^−2^ (50%) (a), 3.80 mW cm^−2^ (75%) (b), 5.10 mW cm^−2^ (100%) (c). PIAF values were also measured at 100% power density and protein concentrations of 1 µM (d), 6 µM (e), and 12 µM (f). Data are expressed as mean ± SD (*n* = 3 independent repeats). Heatmap representations of the PIAF value ratios. (g) Ratio of the PIAF values determined at 3.8 and 5.1 mW cm^−2^ and constant 6 µM CaM (PIAF_ratio,light_ = PIAF_5.1,6 µM_/PIAF_3.8, 6 µM_). (h) PIAF ratios at constant light intensity of 5.1 mW cm^−2^ and protein concentrations of 6 µM and 12 µM (PIAF_ratio,protein_ = PIAF_5.1,12 µM_/PIAF_5.1,6 µM_). Red and blue colors indicate values above and below 1.0, respectively.

To probe this effect, total replicator concentrations and PIAF values were determined at different irradiation intensities and protein concentrations (Figures 6a–f, Tables S9 and S10). With 6 µM CaM, the total steady‐state replicator concentrations were 1.8, 5.5, and 32.5 µM at power densities of 2.55, 3.8, and 5.1 mW cm^−2^, respectively. These data show that the total replicator and CaM concentrations are roughly comparable at 3.8 mW cm^−2^, a regime where competitive selection is not expected to dominate. At 5.1 mW cm^−2^, however, the replicator‐to‐CaM ratio reaches about 5, making competition between sequences likely. Consistent with this, PIAF values did not increase monotonically for all replicators when the light intensity was raised from 3.8 to 5.1 mW cm^−2^ at 6 µM CaM, in line with competition for limited protein binding sites. This effect was visualized in a heatmap showing the ratio of PIAF values at 3.8 and 5.1 mW cm^−2^ under constant 6 µM CaM (PIAF_ratio,light_ = PIAF_5.1,6 µM_ / PIAF_3.8,6 µM_). Values above 1.0 highlight replicators that boost their PIAF with higher light intensity, thanks to effective competition for CaM binding sites (Figure 6g). By contrast, several replicators showed PIAF_ratio,light_ below 1.0, indicating their displacement from the protein's catalytic sites at elevated light levels.

To test whether doubling the protein concentration reduces replicator competition, PIAF ratios were calculated at a fixed light intensity of 5.1 mW cm^−2^ and CaM concentrations of 6 µM and 12 µM (PIAF_ratio,protein_ = PIAF_5.1,12 µM_ / PIAF_5.1,6 µM_). As anticipated, the higher CaM level produced a more even PIAF distribution (Figure 6f) by accelerating replication for weaker replicators (Figure 6h). Meanwhile, the top replicator's PIAF dropped (PIAF_ratio,protein_ < 1.0), creating a nearly inverse pattern compared to the PIAF_ratio,light_ heatmap. Closer analysis revealed that top performers were only mildly suppressed (to ∼80% of the original PIAF), while poor replicators gained 2.0–2.5‐fold boosts. Given the 47.4 µM total replicator concentration at 12 µM CaM and 5.1 mW cm^−2^, protein binding sites remain limiting—explaining the modest suppression relative to the gains.

**FIGURE 7:**
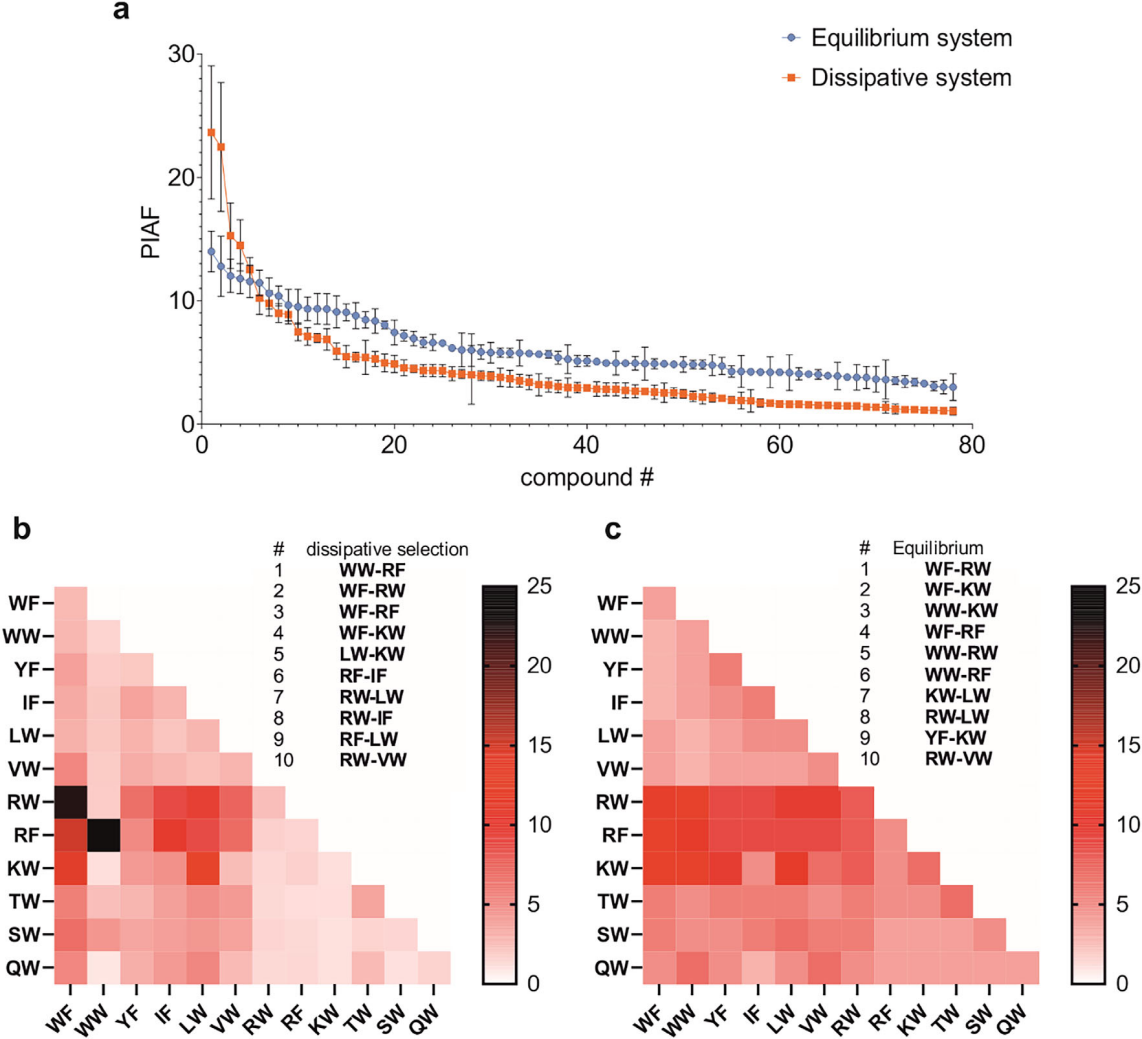
Selectivity of the protein‐induced amplification in the dissipative and the equilibrium systems. (a) Ordered PIAF profile obtained for the light‐driven dissipative system at 6 µM CaM and light intensity of 5.1 mW cm^−2^ (orange). Ordered PIAF profile measured at 6 µM CaM for the equilibrium system (blue). Precursor concentrations were identical. Data are expressed as mean ± SD (*n* = 3) and are given in Tables S10 and S11. PIAF values in heatmap representation and the ten most amplified dimers for the light‐driven dissipative replication (b) and for the equilibrium system **(c**). Concentrations of the dimers at steady‐state and in equilibrium are shown in Figure S13 and Table S12.

Studies on equilibrium dynamic covalent libraries (DCLs) show that component diversity and affinities dictate amplification factors [31], with selectivity heavily influenced by template concentrations [32, 33]. Distinguishing affinity‐driven equilibrium effects from nonequilibrium dissipative selection is thus critical. To that end, the UV‐driven dissipative system was directly compared to a thermodynamic control using thiolate‐mediated nucleophilic exchange in glutathione redox buffer at pH 8.0 [26], with identical initial monomers and 6 µM CaM. PIAF distributions highlighted the light‐driven system's superior selectivity via competitive selection (Figure 7a, Table S11). Selectivity (PIAF_max_/PIAF_min_) reached 4.7 in the equilibrium DCL (PIAF_max_ = 14.0, PIAF_min_ = 3.0; Figure 7c), versus 22.3 in the dissipative system at 5.1 mW cm^−2^ (PIAF_max_ = 23.6, PIAF_min_ = 1.1; Figure 7b). These findings indicate increased selectivity for the dissipative system. Total dimer concentration was higher in equilibrium DCL (49.2 µM) than in the dissipative case (32.5 µM). Equilibrium affinities and dimer excess over protein thus fail to account for the dissipative system's selectivity, which arises from amplified, nonequilibrium competitive mechanisms, where affinities propagate through complex dynamics. Although dissipative and thermodynamic control mechanisms differ fundamentally, the sets of selected ligands overlap significantly (Figures 7b,c insets). This suggests that dissipative competitive selection is closely linked to the affinity of molecular recognition steps. Yet, increasing energy input pushes the system to achieve selectivity far from equilibrium. Two of the top ligands (**WW‐RF** and **WF‐RW**) were independently examined for their affinities to the template, revealing low nanomolar binding at the protein's orthosteric site (Figure S10).

The results imply that competitive selection shapes the replicator distribution later in the process when dimers outnumber active catalytic sites. This temporal evolution of the replicator population is supported by comparing PIAF values at 1 h versus steady state under full light intensity (Figure S11a). The heatmaps show distinct selection patterns. The five most amplified replicators at 1 h (**WW‐LW, VW‐RF, LW‐RW, LW‐KW, VW‐RW**) differ completely from those dominating at steady state (**WF‐RW, WW‐RF, WF‐RF, WF‐KW, LW‐KW**). To explore how the initial replicator population influences competitive selection, we separated the effects of protein‐driven selection from seeding. An initial replicator population was generated in a protein‐free sample and then used to seed both protein‐catalyzed and protein‐free systems. Using consistent seeding allowed us to isolate catalysis‐driven competitive selection effects by comparing PIAF profiles between seeded and non‐seeded protein‐containing samples (Figure S12a). This comparison revealed that initial replicator populations affect steady‐state distributions. Although an entirely new steady state did not form after seeding, statistically significant concentration differences emerged for several replicators. Notably, seeding suppressed hydrophobic replicators while enhancing those with Arg and Lys side chains (Figures S12b and S12c). The seeded and unseeded systems followed distinct evolutionary paths, leading to different steady states, highlighting that initial population composition shapes competitive selection.

Adaptive, out‐of‐equilibrium selection of chemical replicators requires operation within a replication–destruction regime, allowing continuous rearrangement of components [1, 9]. Regardless of the specific degradation pathway, this regime inevitably restricts the overall population growth of replicators, even when replication follows purely exponential kinetics [2, 34, 35]. Consequently, selection based solely on exponential growth cannot occur under these conditions, and competitive selection among chemical replicators must rely on additional mechanisms.

In known systems, the crucial autocatalytic replication step depends exclusively on interactions between the replicator products and their precursors [6, 16, 18, 36, 37]. Hence, changes in reaction conditions lead to selective behavior primarily due to differences in precursor reactivity toward various replicator structures and differences in replicator destruction rates, or both. *Otto and colleagues* demonstrated that when multiple replicators compete for a shared precursor pool, competitive exclusion arises under oxidative, out‐of‐equilibrium conditions in disulfide‐based replication systems. Serial transfer into thiol‐containing vessels enhanced the destruction mechanism [17]. A comparable adaptive competition was observed between foldamer structures and templated autocatalytic fibril assembly, where stepwise addition of TCEP promoted replicator elimination [38]. When the oxidative environment was modified by introducing a dye‐sensitized photocatalytic system capable of generating singlet oxygen in a continuously stirred‐tank reactor (CSTR), the disulfide replicator system adaptively shifted its preference toward the kinetically favored replicator structure under oxidative pressure [12]. Similarly, *Semenov and co‐workers* reported an out‐of‐equilibrium selection process during the autocatalytic formation of thioester macrocycles [19]. In their system, negative feedback was achieved via acrylamide‐mediated thiol removal in a CSTR. The competition for a shared building block—paired with a kinetic preference for the destruction pathway—led to the selection of a specific macrocycle within the replication–destruction framework. *Fletcher and co‐workers* also described selection mechanisms in autocatalytic surfactant formation, where precursor reactivity dictated the outcome [39, 40]. In these kinetically controlled systems, diverse reaction pathways enabled rapid replication, giving rise to selective behavior.

In the present system, a protein catalyst participates in the critical replication step, binding both the template and the precursors. By enhancing proximity control, the catalyst accelerates replication and introduces an additional selection factor through molecular recognition. As a result, selectivity depends not only on differences in replicators’ intrinsic autocatalytic rates and their rates of degradation but also on competition for the catalyst's binding sites. While effector‐induced selection is well established in DCLs [41, 42, 43], the light‐driven, dissipative nature of this system situates the selection process in a distinct context. Increasing energy input drives competition for the protein binding sites beyond the equilibrium distribution dictated by binding free energy. Consequently, the protein‐mediated replication selectivity—comparing the most and least favored replicators—increased by a factor of 6.9 when transitioning from an equilibrium DCL to the light‐driven dissipative system.

Sequence‐dependent selection in this system occurs among a relatively large population of replicators (78 variants). Considering the entropic barrier associated with such diversity, the protein‐catalyzed, dissipative system shows robust selectivity compared to previous literature examples that involve only a small number of structures. The replicator building blocks are foldamers that mimic protein‐surface fragments [30]. These primitive sequences encode a minimal form of structural information that can be interpreted by other interacting molecules, such as proteins [44] or foldamers. On this basis, they can be viewed as primitive, information‐carrying replicators of nonbiological origin [43].

## 3 Conclusion

In this work, a light‐driven dissipative replicator system was constructed in which autocatalytic replication cycles of peptides are catalyzed within a protein environment. The promiscuous and flexible nature of the model protein catalyst, CaM, together with the strong helical folding tendency of the short foldamer precursors, enables simultaneous interaction between the protein, the replicator template, and the precursors. This coupling between the catalyst and the replication cycles provides an experimental model for a key mechanistic element of Eigen's hypercycle theory. Although perfectly specific replicator–catalyst interactions are not attainable in such a primitive system, the combination of catalyzed replication with dissipative dynamics leads to effective replicator selection that depends on both the catalyst and the energy input. This behavior represents a new form of competitive selection that appears to be an intrinsic consequence of catalyst–replicator interactions in evolvable chemical systems. The results strongly suggest that selectively coupling primitive replicator cycles through catalysis requires dissipative conditions in order to approximate the specific hypercycle interactions originally proposed by Eigen.

## Funding

This research was funded by the National Research, Development and Innovation Office of Hungary, PharmaLab RRF‐2.3.1‐21‐2022‐00015, NKKP_ADVANCED 153477 and 2020–1.1.6‐JÖVŐ‐2021‐00004 (G.M.K.), NKFIH K135150 (G.M.K.), 2018–1.2.1‐ NKP‐2018‐00005 (HunProtExc) financed under the 2018– 1.2.1‐NKP funding Scheme. Project no TKP2021‐EGA‐32 has been implemented with the support provided by the Ministry of Culture and Innovation of Hungary from the National Research, Development and Innovation Fund, financed under the TKP2021‐EGA funding scheme (T.A.M.). Project number RRF‐2.3.1‐21‐2022‐00015 is implemented with the support of the European Union's Recovery and Resilience Instrument. This research work was conducted with the support of the National Academy of Scientist Education Program of the National Biomedical Foundation under the sponsorship of the Hungarian Ministry of Culture and Innovation. The work of E.B. was supported by the Postdoctoral Research Grant of the Albert Szent‐Györgyi Medical School, University of Szeged. The work was supported by the University of Szeged Open Access Fund (Grant Number: 7311).

## Conflicts of Interest

The authors declare no conflict of interest.

## Supporting information




**Supporting File 1**: The authors have cited additional references within the Supporting Information [45, 46, 47, 48, 49].

## Data Availability

The data that support the findings of this study are available in the Supporting Information of this article.
