# Supplementary material for: Light‐Driven Competitive Selection in a Protein‐Catalyzed Dissipative Peptide Replication
Source: Angew Chem Int Ed Engl. 2026 Feb 1;65(18):e18911. doi: 10.1002/anie.202518911 (PMC13110758; doi:10.1002/anie.202518911)
Supplement: Supplementary file 1 — Supporting File 1: The authors have cited additional references within the Supporting Information [45, 46, 47, 48, 49]. [file ANIE-65-e18911-s001.docx]

Supplementary Information for

**Light-Driven Competitive Selection in a Protein-Catalyzed Dissipative Peptide Replication**

Éva Bartus^a^, Edit Wéber^a,b^, Attila Tököli^a,c^, Ferenc Bogár^a,b^, Momen R. F. Mohamed^a,d^, Gábor Kecskeméti^a^, Zoltán Szabó^a^, Zoltán Kele^a^, András Perczel^e,f,g^, Márton Gadanecz^e,g,h^, Zoltán Orgován^g,i^, György M. Keserű^g,i,j^, Tamás A. Martinek^a,b^*

[a] É. Bartus, E. Wéber, A. Tököli, F. Bogár, M. R. F. Mohamed, G. Kecskeméti, Z. Szabó, Z. Kele, Prof. T. A. Martinek
Department of Medical Chemistry
University of Szeged
Dóm tér 8, H-6720 Szeged, Hungary
E-mail: martinek.tamas@ med.u-szeged.hu

[b] E. Wéber, F. Bogár, Prof. T. A. Martinek
HUN-REN-SZTE Biomimetic Systems Research Group
University of Szeged
Dóm tér 8, H-6720 Szeged, Hungary

[c] A. Tököli
University of Cambridge
Department of Biochemistry
Hopkin Building, Downing Site
Tennis Court Road, Cambridge CB2 1QW, UK

[d] M. R. F. Mohamed
Department of Medicinal Chemistry
Faculty of Pharmacy, Minia University,
Minia 61519, Egypt

[e] Prof. A. Perczel, M. Gadanecz
Laboratory of Structural Chemistry and Biology
Institute of Chemistry, Eötvös Loránd University
Pázmány Péter sétány 1/A, H-1117 Budapest, Hungary

[f] Prof. A. Perczel
HUN-REN-ELTE Protein Modeling Research Group
Institute of Chemistry, Eötvös Loránd University
Pázmány Péter sétány 1/A, H-1117 Budapest, Hungary

[g] Prof. A. Perczel, M. Gadanecz, Z. Orgován, Prof. G. M. Keserű
HUN-REN Research Centre for Natural Sciences, Drug Innovation Centre and National Drug Discovery and Development Laboratory, Magyar Tudósok krt. 2, H-1117 Budapest, Hungary

[h] M. Gadanecz
Hevesy György PhD School of Chemistry
Institute of Chemistry, Eötvös Loránd University
Pázmány Péter sétány 1/A, H-1117 Budapest, Hungary

[i] Z. Orgován, Prof. G. M. Keserű
Medicinal Chemistry Research Group
HUN-REN Research Centre for Natural Sciences
Magyar Tudósok krt. 2, H-1117 Budapest, Hungary

[j] Prof. G. M. Keserű
Department of Organic Chemistry and Technology
Faculty of Chemical Technology and Biotechnology, Budapest University of Technology and Economics
Budafoki út 8, H-1111 Budapest, Hungary

Table of Contents

[Experimental procedures 4](#_Toc216172081)

[Synthesis and purification of the foldameric sequences 4](#_Toc216172082)

[Synthesis and purification of the glutathione protected monomers 4](#_Toc216172083)

[CaM expression and purification 4](#_Toc216172084)

[K-Ras G12D expression and purification 5](#_Toc216172085)

[UV-light-driven disulfide exchange reaction in the presence of CaM 5](#_Toc216172086)

[Seeding experiments 5](#_Toc216172087)

[Calculation of significance of the kinetic curve differences between the seeded and non-seeded protein-catalyzed samples 6](#_Toc216172088)

[UV-light-driven disulfide exchange reaction in the presence of K-Ras G12D 6](#_Toc216172089)

[LC-MS measurements and MS data analysis 6](#_Toc216172090)

[Testing the sensitivity of CaM against UV irradiation 7](#_Toc216172091)

[Proteomic analysis of the oxidized CaM samples 7](#_Toc216172092)

[Photo-foldamer screening study 7](#_Toc216172093)

[Binding site localization by mass spectrometric analysis 8](#_Toc216172094)

[Isothermal titration calorimetry (ITC) experiments 9](#_Toc216172095)

[Supplementary Text 10](#_Toc216172096)

[Equation (S4): Rate of spontaneous dimer synthesis through diffusion-controlled radical substitution 11](#_Toc216172097)

[Equation (S8): Rate of dimer breakdown through diffusion-controlled radical substitution 11](#_Toc216172098)

[Equations (S11) and (S12): Rates of dimer synthesis through proximity-controlled mechanisms 12](#_Toc216172099)

[Equations (S14) and (S15): Rates of autocatalytic dimer synthesis 13](#_Toc216172100)

[Dynamic model for the foldamer-based photochemical disulfide exchange system 13](#_Toc216172101)

[Fitting the dynamic model to the experimental time- and energy-dependent data arrays 13](#_Toc216172102)

[ITC characterization of the binding of foldameric dimers to CaM 14](#_Toc216172103)

[Supplementary Figures 15](#_Toc216172104)

[Figure S1. Schematic representation of protein-induced photochemical disulfide exchange reactions. 15](#_Toc216172105)

[Figure S2. Monitoring the UV-induced degradation of CaM 16](#_Toc216172106)

[Figure S3 Fitting of the dynamic model of chemical evolution to experimental data for representative replicators 22](#_Toc216172107)

[Figure S4. Effect of autocatalysis on protein-induced dimer formation. 22](#_Toc216172108)

[Figure S5. Results of the seeding experiments. 28](#_Toc216172109)

[Figure S7. Seeding effect of WF-S-RF on protein-free foldamer system. 35](#_Toc216172110)

[Figure S8. Time-dependent data array for representative replicators in a seeding experiment. 36](#_Toc216172111)

[Figure S9. Results of the UV-light-driven disulfide exchange reaction in the presence of K-Ras G12D. 37](#_Toc216172112)

[Figure S10. ITC competitive and control titrations for WF-S-RW and WW-S-RW ligands. 38](#_Toc216172113)

[Figure S11. Evolution of replicator population in time. 39](#_Toc216172114)

[Figure S12. Effect of seeding (initial replicator population) on competitive selection 40](#_Toc216172115)

[Figure S13. Comparison of product distributions in the thermodynamically controlled and light-driven systems. 41](#_Toc216172116)

[Figure S14. Comparison of product distribution shown in heatmap representation. 42](#_Toc216172117)

[Supplementary Tables 43](#_Toc216172118)

[Table S1. Identification of the oxidized Met residues in the CaM sequence. 43](#_Toc216172119)

[Table S2. Light intensity-dependent amplifications (LIAF) obtained upon increasing power density from 2.55 to 5.10 mW cm^-2^ for control and CaM containing sample 43](#_Toc216172120)

[Table S3. Fitted rate constants for representative dimers without the protein template. 45](#_Toc216172121)

[Table S4. Fitted rate constants for representative dimers with the protein template. 46](#_Toc216172122)

[Table S5. Product distribution of the seeding pre-irradiated mixture 47](#_Toc216172123)

[Table S6. Concentration amplifications (CA) for the MSSM dimers upon seeding with the pre-irradiated mixture at 40 min. 48](#_Toc216172124)

[Table S7. Binding site analysis of the photo-foldamers on CaM. 49](#_Toc216172125)

[Table S8. Characterization of the glutathione-protected monomers for K-Ras G12D. 50](#_Toc216172126)

[Table S9. Protein-induced amplification factors (PIAFs) of the building blocks in steady state measured with different power densities 50](#_Toc216172127)

[Table S10. Protein-induced amplification factors of the building blocks in steady state as a function of CaM concentration 52](#_Toc216172128)

[Table S11. Protein-induced amplification factors (PIAFs) of the building blocks in equilibrium 53](#_Toc216172129)

[Table S12. Product distribution in the thermodynamically controlled system and in the UV-induced system 54](#_Toc216172130)

[Peptide characterization data 56](#_Toc216172131)

[Supporting references 62](#_Toc216172132)

[Author Contributions 62](#_Toc216172133)

[References 62](#_Toc216172134)

# Experimental procedures

## Synthesis and purification of the foldameric sequences

Production of the foldameric sequence was performed as described previously^[1]^. Briefly, Foldameric sequences were synthesized manually by standard solid-phase peptide synthesis with Fmoc/tBu chemistry. Rink Amide AM resin was used as solid support (capacity: 0.71 mmol/g) and HATU (1-[bis(dimethylamino)methylene]-1*H*-1,2,3-triazolo[4,5-b]pyridinium 3-oxid hexafluorophosphate) as coupling reagent in the presence of DIEA (*N,N*-diisopropylethylamine). Amino acids and coupling reagents were used in excess of 3 equivalents and shaking was applied at room temperature for 3 h. Deprotection was carried out in a DMF (*N,N*-dimethylformamide) solution containing 2% DBU (1,8-diazabicycloundec-7-ene) and 2% piperidine. Cleavage was performed with TFA/H_2_O/DTT (DL-dithiothreitol)/TIS (triisopropylsilane) (90:5:2.5:2.5), which was followed by precipitation in ice-cold diethyl ether. The resin was washed with acetic acid and water, filtered, then lyophilized. Peptides were purified by RP-HPLC on a C18 column (Phenomenex Luna C18, 250 x 10.00 mm, particle size: 10 μm, pore size: 100 Å). The HPLC eluents were 0.1% TFA in water (Eluent A), and 0.1% TFA/ 80% ACN (acetonitrile) in water (Eluent B). Different gradient elution was used according to the hydrophobicity of the peptides. Purity was confirmed by analytical RP-HPLC and ESI-MS measurements.

## Synthesis and purification of the glutathione protected monomers

Glutathione-protected monomers were synthesized by oxidative coupling of thiols in the solution phase according to the protocol described previously^[1]^. Purified foldamers were dissolved in 20% DMSO (dimethyl sulfoxide) in water separately to 1 mM concentration in the presence of 20 times excess of GSH (reduced glutathione) and stirred overnight at room temperature exposed to atmospheric oxygen. The completeness of the oxidation reaction was monitored by HPLC-MS, and the reaction mixture was injected directly onto a semi-preparative HPLC column (Phenomenex Luna C18, 250 x 10.00 mm; particle size: 10 μm; pore size: 100 Å) and purified. Under this reaction condition the amount of the homodimeric foldamer was negligible and could be completely separated from the foldamer-glutathione adduct.

## CaM expression and purification

Calmodulin (CaM) (bovine) gene was cloned into pET28a vector. The sequenced plasmid was then transformed to competent *E. coli* cells (BL21 DE3) for protein expression. Cells were grown on LB liquid media at 37°C until OD600 = 0.5, then expression of CaM was induced by adding 200 μM IPTG and was carried out overnight (~19 hours) at 22°C. After centrifugation, cell pellets were resuspended in Ni-NTA Lysis Buffer (50 mM NaH_2_PO_4_, 300 mM NaCl, 10 mM imidazole, pH 8.0) and were lysed by sonication with addition of 1 μM Leupeptin, 0.1 μg ml-1 Pepstatin A and 20 μM PMSF (Phenylmethylsulfonyl fluoride). The cleared lysate was first purified using a Ni-NTA filled column according to the manufacturer’s protocol (Expression and purification of proteins using 6×Histidine-tag): After equilibration of the Ni-NTA column, lysate was added for a short incubation on ice (30 minutes), washed with Ni-NTA Wash Buffer (50 mM NaH_2_PO_4_, 300 mM NaCl, 20 mM imidazole, pH 8.0), and finally CaM was eluted with small volumes of Ni-NTA Elution buffer (50 mM NaH_2_PO_4_, 300 mM NaCl, 250 mM imidazole, pH 8.0).The clear fractions were concentrated using Amicon Ultra Filter Device (10K) and the buffer was changed to 20 mM HEPES, pH 7.0. The His-tag was removed with an overnight Thrombin digestion at 4 °C leaving 9 extra amino acid (GSHMARSNS) on the N terminus of the protein. Thus, the molecular weight of the protein was 17765.58 g mol^-1^, which was confirmed by ESI-MS measurements. In order to remove additional cleavage fragments after thrombin treatment CaM was purified by using RP-HPLC on a C4 column (Phenomenex, Jupiter 250x10 mm), using the following gradient elution: 40-70% Eluent B over 60 minutes at 4 ml min-1 flow rate with A: 0.1% TFA in water and B: 0.1% TFA/80% ACN in water. After lyophilization, the protein was subjected to dialysis in 20 mM, pH 7.0 HEPES buffer in order to remove TFA traces and ensure correct folding. Purity and folding was assessed by HPLC-MS, native ESI-MS and NMR measurements. CaM concentration was measured by using BCA assay (Thermo Scientific, Pierce) utilizing the manufacturer’s protocol.

## K-Ras G12D expression and purification

K-Ras G12D protein was expressed and purified as described previously^[2]^.

## UV-light-driven disulfide exchange reaction in the presence of CaM

Twelve different foldamer-glutathione disulfides (**Mix12**) were dissolved in 20 mM HEPES, 150 mM NaCl, 2 mM CaCl_2_, (pH = 7.0) each one at 10 μM concentration without the protein (control) and with the protein at appropriate concentration. The reaction mixtures were transferred separately into quartz cuvettes having PTFE stopper and kept under argon atmosphere during the experiment. Solutions were stirred at 150 RPM and kept at the constant temperature of 303 ± 1 K *via* air cooling system (modified Jasco Jetstream 2 Plus Column Thermostat). Temperature was monitored with a laser gun thermometer. Continuous illumination of the samples was carried out with UVL-28 EL Series UV Lamp (Analytic Jena US, Upland, CA). The power density and its position/distance dependence and the emission spectra of the lamp were determined before the experiments, and the irradiation intensity was controlled by the distance between the lamp and the sample. 100 μL samples were taken from the reaction mixture at each time point and quenched with 100 μL of 10% TFA in water to significantly slow down any other disulfide exchange reaction in the mixture until the analysis. The product distribution of the samples was analysed by HPLC/ESI-MS measurements.

## Seeding experiments

1. For the first seeding experiment a replicator population was performed rich in dimers of which replication is effectively catalyzed by the protein. Twelve different glutathione protected monomers were dissolved in the above mentioned buffer at 10 μM concentration containing CaM at 6 μM concentration and the mixture was irradiated in a closed quartz cuvette under argon atmosphere with UV light (365 nm, 4.2 mW cm^-2^) for 1 h. 100 μL sample was taken and quenched for HPLC/ESI-MS analysis and product distribution was analyzed by the quantitative evaluation of the chromatograms (Supplementary Table 5). This presynthetized repilcator population was diluted with a freshly prepared reaction mixture (10 μM twelve foldamer-G with 6 μM CaM) in a 1:1 ratio (seeded CaM) and a freshly prepared reaction mixture (10 μM twelve foldamer-G with 6 μM CaM) was started at that time point as a reference (CaM). Light intensity was set to 4.20 mW cm^-2^ and both reaction mixtures were further irradiated with UV-light for 4 h and samples were taken for analysis.
2. Second seeding experiment was carried out with a light insensitive foldameric dimer **WF-S-RW** known to bind to CaM with high affinity. Synthesis and characterization of this thioether dimer was described previously^[1]^. Twelve different glutathione protected monomers were dissolved in 20 mM HEPES, 150 mM NaCl, 2 mM CaCl_2_ (pH = 7.0) each one at 10 μM concentration without the protein (control), with the protein at 6 μM concentration (CaM) and with the protein in the presence of **WF-S-RW** at 6 μM (seeded CaM). The reaction mixtures were transferred separately into quartz cuvettes having PTFE stopper and kept under argon atmosphere during the experiment. Solutions were stirred at 150 RPM and kept at the constant temperature of 303 ± 1 K *via* air cooling system (modified Jasco Jetstream 2 Plus Column Thermostat). Temperature was monitored with a laser gun thermometer. Continuous illumination of the samples was carried out with UVL-28 EL Series UV Lamp (Analytic Jena US, Upland, CA). 100 μL samples were taken from the reaction mixture at 7 time points and quenched with 100 μL of 10% TFA in water:acetonitrile 1:1 to slow down any other disulfide exchange reaction in the mixture until the analysis. The product distribution of the samples was analyzed by HPLC/ESI-MS measurements.
3. To evaluate the effect of initial replicator population on competitive selection we first generated an initial dimer population by irradiating the mixture of precursors (**Mix12**) at 10 μM for 1 h. An aliquot of this pre-formed dimer mixture was then mixed 1:1 with a freshly prepared reaction mixture either in the presence of the protein (protein-catalyzed seeded sample) or in its absence (protein-free seeded sample). The CaM concentration in the protein-containing mixture was adjusted to 12 µM prior to mixing to yield a final concentration of 6 µM after dilution. The seeded reaction mixtures were subsequently irradiated with UV light for an additional 4 h, and samples were collected for analysis as described previously.

## Calculation of significance of the kinetic curve differences between the seeded and non-seeded protein-catalyzed samples

To confirm the presence of autocatalytic replication, we seeded the CaM-catalyzed system with a mixture of pre-synthesized replicators and compared it to the non-seeded system. The dimer concentrations were measured at 7 time points within a 4-hour interval. We aimed to show that the time evolution of the replicator concentrations in the two systems is significantly different for a considerable part of the replicants.

To this end, optimal parameters of our kinetic model were obtained by weighted least-squares fitting to the experimental concentration data using least_squares_fitting procedure from the scipy**^[3]^.** Python package with a trust-region reflective algorithm**^[4]^** and non-negativity constraints. The confidence intervals of curve fitting were estimated using Monte Carlo simulation, a stochastic resampling method that directly incorporates empirical measurement error distributions**^[4]^**. Synthetic datasets were generated by corrupting fitted model predictions with measurement noise sampled from observed standard deviations. The kinetic model was independently refitted to each of 1000 synthetic datasets using identical optimization procedures. Confidence intervals (95%) were computed as percentiles of the Monte Carlo ensemble. Comparison between seeded and non-seeded datasets was performed via confidence interval overlap; non-overlapping CIs indicate statistically significant differences. The ODE system was integrated using scipy.integrate.solve_ivp with LSODA method^[5]^**.** All analyses were implemented in Python 3.10 using NumPy, SciPy, Pandas, and Matplotlib.

## UV-light-driven disulfide exchange reaction in the presence of K-Ras G12D

Eight different glutathione protected monomers **(QR-G, LR-G, WR-G, QF-G, QW-G, WQ-G, VF-G, VW-G)** were dissolved in 20 mM HEPES, 150 mM NaCl, 10 mM MgCl_2_, (pH = 7.0) each one at 15 μM concentration without the protein (control) and K-Ras G12D at 15 μM concentration. The reaction was carried out as described in the case of CaM induced setup: reaction mixtures were transferred separately into quartz cuvettes having PTFE stopper and kept under argon atmosphere during the experiment. Solutions were stirred at 150 RPM and kept at the constant temperature of 303 ± 1 K *via* air cooling system (modified Jasco Jetstream 2 Plus Column Thermostat). Temperature was monitored with a laser gun thermometer. Continuous illumination of the samples was carried out with UVL-28 EL Series UV Lamp (Analytic Jena US, Upland, CA). The power density was set to 5.1 mW cm^-2^. 100 μL samples were taken from the reaction mixture at each time point and quenched with 100 μL of 10% TFA in water to significantly slow down any other disulfide exchange reaction in the mixture until the analysis. The product distribution of the samples was analyzed by HPLC/ESI-MS measurements.

## LC-MS measurements and MS data analysis

LC-MS analysis was performed with a Dionex UltiMate 3000 HPLC system interfaced to an LTQ ion trap mass spectrometer (Thermo Electron Corp., San Jose, CA, USA). Analytic procedure was the same as described previously^[1]^. Samples were injected onto an AerisTM Widepore XB-C18 (250 x 4.6 mm, particle size: 3.6 μm, pore size 100Å) analytical HPLC column using gradient elution 5-80% solution B during 25 minutes at 0.7 mL min^-1^ flow rate. Eluent composition was 0.1% formic acid in distilled water (Solution A) and 0.1% formic acid in acetonitrile (Solution B). The MS instrument was operated in the positive-ion mode using the equipped HESI-II source with the following parameters: capillary temperature: 350°C; spray voltage: 3.0 kV; source heater temperature: 250°C; sheath gas flow: 30 mL min^-1^; aux gas flow: 10 mL min_-1_. Mass spectra were acquired in full scan mode from 200 to 2000 m/z range. Thermo Xcalibur 2.2 software was used for peak identification and integration. The 96% of the foldameric building blocks could be resolved independently via HPLC-MS/MS measurements based on molecular weight, MS fragmentation pattern and retention time considering the relative hydrophobicity of the sidechains. Those components which could not be resolved independently were integrated together and averaged. A representative raw file for the library was utilized to create a processing method where each sample component was associated with a chromatographic peak based on the previously identified mass (m/z) and retention time^[1]^. Using the ICIS peak detection algorithm, the general detection and integration criteria were smoothing points: 5, baseline window: 60, area noise factor: 5, peak noise factor: 10. All raw data files were reprocessed with these processing setups together and analyzed. Errors in peak identification during the automatic processing were corrected manually.

## Testing the sensitivity of CaM against UV irradiation

CaM in a concentration of 6 μM was irradiated with UV light (365 nm, 5.1 mW cm^-2^) for 11 h under argon atmosphere and samples were taken in every hour to monitor the stability of the protein. Samples were measured directly with HPLC-MS technique. Besides the intact protein (CaM, 17765.1992 Da) the mass of the presumably oxidized protein was detectable, expected to be Met oxidized products after 5 h irradiation (Figure S2). In order to identify the affected Met residues in the oxidation, after the enzymatic digestion (Trypsin protease) of the protein, samples were analyzed *via* HPLC-MS/MS. List of the resulted peptidic fragment sequences with their calculated mass was generated with ProteinProspector v 6.2.1 MS-Digest webpage (University of California, San Francisco, CA, USA). Presence and intensity of the oxidized fragments were characterized with the area under the curve (AUC) values and expressed in relative % to the intact protein (Supplementary Table 1). However, almost all of the 11 Met in the sequence were affected in the oxidation, 95% of the protein remain intact after 5 h irradiation. Especially the Met^119^ was affected of which 4.8% was oxidized after 5 h UV irradiation but it has a minor effect in ligand binding^[6]^.

Sequence of CaM (calculated MW= 17765.43 g mol^-1^): GSHM^4^ARSNSM^10^ADQLTEEQIAEFKEAFSLFDKDGDGTITTKELGTVM^46^RSLGQNPTEAELQDM^61^INEVDADGNGTIDFPEFLTM^81^M^82^ARKM^86^KDTDSEEEIREAFRVFDKDGNGYISAAELRHVM^119^TNLGEKLTDEEVDEM^134^IREADIDGDGQVNYEEFVQM^154^M^155^TAK

## Proteomic analysis of the oxidized CaM samples

After enzymatic digestion the product composition was analyzed with UHPLC-MS/MS measurements using an ACQUITY I-Class UPLC™ liquid chromatography system (Waters, Manchester, UK) coupled with a Q Exactive™ Plus Hybrid Quadrupole-Orbitrap Mass Spectrometer (Thermo Fisher Scientific, San Jose, CA, USA). Chromatographic separation was carried out at 25 °C using 0.1% formic acid in water as solvent A and ACN containing 0.1% formic acid as solvent B. The following multistep gradient was used: 5-50% over 20 minutes then 50-80% over 5 minutes and finally 80% solvent B for an additional 5 minutes at 0.7 mL min^-1^ flow rate. Samples where incubated at 5°C until the measurement and 15 μL of the sample was injected into the UHPLC–MS/MS system. The MS instrument was operated in the positive-ion mode using the equipped HESI-II source with the following parameters: capillary temperature: 256°C; spray voltage: 3.5 kV; aux gas heater temperature: 412°C; sheath gas flow: 47.5 mL min^-1^; aux gas flow: 11 mL min^-1^; and S-lens RF level, 50.0 (source auto-defaults). Full scan was conducted with a mass range of 150–2000 *m/z* with resolution of 70,000. The ACG (automatic gain control) setting was defined as 3 × 10^6^ charges, and the maximum injection time was set to 100 ms. Data dependent MS/MS was acquired in a mass range of 200-2000 *m/z* with a resolution of 17,500. AGC setting was defined as 5 × 10^5^ charges, and the maximum injection time was set to 150 ms.

## Photo-foldamer screening study

**Sample preparation.** Stock solutions were prepared in DMSO at 8 and 10 mM concentrations from the 12 photo-foldamers (**ph-IF, ph-IF, ph-KW, ph-LW, ph-QW, ph-RW, ph-RF, ph-SW, ph-TW, ph-VW, ph-WF, ph-WW** and **ph-YF**). CaM stock solution was prepared at 6.67 μM concentration in the following buffer: 20 mM HEPES with 150 mM NaCl and 2 mM CaCl_2_, pH 7.0 and the target concentration of the protein was 6 μM photo-foldamers were applied at two different concentrations: 50 μM, and 120 μM. For competitive crosslinking experiments CaM was applied at 6 μM, photo-foldamers were applied at 50 μM concentrations in the presence of **WF-S-RW** at 20 μM concentration.

Experiments were performed in 96-well Flat-Bottom Microplates. 90 µL protein solution was pipetted to each well than photo-foldamer stock and additional buffer was added to get the final concentration in 100 µL/well. DMSO amount was maximized at 1%. Proteins were incubated with photo-foldamers for 20 minutes at room temperature in the dark. Subsequently, samples were irradiated with a LED UV light (38 W) at 365 nm for 10 minutes.

**Analysis of the samples.** Samples were directly analyzed without enzymatic digestion. LC-MS analysis was performed with a Dionex UltiMate 3000 HPLC system interfaced with an LTQ ion trap mass spectrometer (Thermo Electron Corp., San Jose, CA, USA). Samples were injected onto a C4 column (BioZen 2.6 μm WidePore C4; 150 x 2.1 mm). The HPLC eluents were 0.1% formic acid in water (Eluent A), and 0.1% formic acid in acetonitrile (Eluent B). Gradient elution started at 20% Eluent B, and after 10 minutes, 90% was reached with 0.4 mL min^-1^ flow. The MS instrument operated in the positive-ion mode using the equipped HESI-II source with the following parameters: capillary temperature: 350°C; spray voltage: 3.0 kV; source heater temperature: 250°C; sheath gas flow: 30 mL min^-1^; aux gas flow: 10 mL min^-1^. Mass spectra were acquired in full-scan mode from 200 to 2000 *m/z*. UniDec 5.0.2 software^[7]^ as used for peak deconvolution, with a mass range adjusted between 15000 and 25000 Da. Peak normalization was adjusted on total mode.

Crosslinking yields were calculated using the following formula:

$$crosslinking yield \left( \% \right)=\frac{I_{crosslinked protein}}{I_{crosslinked protein}+I_{non-labeled protein}}*100\%$$

where I_crosslinked protein_ and I_non-labeled protein_ are the intensities of the photo-foldamer-labelled and the non-labelled CaM. The standard error of mean in the crosslinking yields across three replicates was below 2%.

## Binding site localization by mass spectrometric analysis

**Sample preparation.** Two sample series were prepared in parallel by using the following buffer: 20 mM HEPES, 150 mM NaCl and 2 mM CaCl_2,_ pH 7.0: i) 6 μM of CaM with 120 μM of photo-foldamer and ii) 6 μM of CaM, 120 μM of photo-foldamer and 6 μM of **WF-S-RW** as a competitor. Samples were preincubated at room temperature for 20 min and then irradiated with UV light at 365 nm for 10 minutes. Samples containing 5 µg protein were processed by on-pellet digestion. Briefly, the samples were reduced with 10 mM dithiothreitol (DTT) at 60 °C for 30 min and alkylated with 20 mM iodoacetamide (IAA) in the dark at room temperature for 30 min. The protein content was precipitated by adding a 7-fold volume of ice-cold acetone and incubated at −20°C overnight. The supernatant was discarded after centrifugation with 15,000 x g, 10 min, 4°C. The protein pellet was washed twice with 500 µL acetone/water (85:15, v/v). After centrifugation with 14,000 x g, 10 min, 4°C, the protein pellet was dissolved in 80 µL Tris-Formic acid buffer (50 mM, pH 8). A total of 0.25 µg trypsin was added to the samples in two steps and incubated at 37°C for 6 hours. Digestion was stopped by the addition of 1 µL concentrated formic acid. After centrifugation, 1 µL of the supernatant was injected into the Nano-Liquid Chromatography-Mass Spectrometry (nanoLC–MS) system.

**LC-MS/MS analysis**. NanoLC–MS analysis was carried out on a Waters ACQUITY UPLC M-Class LC system (Waters, Milford, MA, United States) coupled with an Orbitrap Exploris™ 240 mass spectrometer (Thermo Fisher Scientific, Waltham, MA, United States). Symmetry® C18 (100 Å, 5 µm, 180 µm × 20 mm) trap column was used for trapping and desalting the samples. Chromatographic separation of peptides was accomplished on an ACQUITY UPLC® M-Class Peptide BEH C18 analytical column (130 Å, 1.7 µm, 75 µm × 250 mm) at 45°C by gradient elution. Water (solvent A) and acetonitrile (solvent B) containing 0.1% formic acid were used as mobile phases at a flow rate of 200 nL min^-1^. The sample temperature was maintained at 5°C. The mass spectrometer operated in positive mode using the equipped Nanospray Flex Ion Source. Data were collected using the data-dependent acquisition (DDA) method in the 360-2200 m/z range, and preferred charges states were set between +2 and +6. MS spectra were acquired with a resolution of 60,000, while MS/MS spectra were collected with a resolution of 30,000. The collision energy was stepped between 16, 24, and 32 NCE. The maximum inject time was set to auto. Data was acquired using XcaliburTM 4.6 (Thermo Fisher Scientific, Waltham, MA, United States).

A peptide identification database search was performed on raw MS/MS data files using FragPipe version 22.0. Default mass offset search settings were applied, specifying all photo-foldamer modifier masses, methionine oxidation, and cysteine carbamidation as possible mass offsets with a mass precision of 10 ppm. A false discovery rate (FDR) filter of 0.01 was applied to peptide matches. Peptide intensities were calculated by the IonQuant component of FragPipe using default settings.

## Isothermal titration calorimetry (ITC) experiments

ITC experiments were carried out with a MicroCalTM VP-ITC microcalorimeter (GE Healthcare, Northampton, MA, USA). **WF-S-RW** (MW: 2151.26 g mol^-1^) and **WW-S-RF** (MW: 2151.26 g mol^-1^) conjugates were dissolved in 20 mM HEPES, 150 mM NaCl, 5 mM CaCl_2_ pH=7.4 buffer and they were sonicated for 20 minutes before titration to avoid aggregation. Foldamer solution (15 μl) was injected from the computer-controlled microsyringe into the CaM solution at intervals of 240 s. Concentration of CaM in the cell was 3 μM, and the concentration of foldamers in the syringe was 75-80 μM. The temperature was adjusted to 303 K. The control experiments were performed by injecting foldamers into the cell containing buffer with no target. Experiments were repeated twice. The experimental data were fitted to the two independent sites model (adjustable parameters: ΔH_b1_, K_d1_, n_1_ and ΔH_b2_, K_d2_, n_2_) using a nonlinear least-squares procedure. Errors were calculated via jackknife resampling.

# Supplementary Text

Scheme S1. Reaction mechanism of the Dynamic Model^[[1]](#footnote-2)^

**Exchange by chain reaction (diffusion control)**

| $MSSG\begin{matrix} +h\nu\\ \rightleftarrows\\ -\Delta\end{matrix}MS· + GS\boldsymbol{·}$ | [1a,b] |
| --- | --- |
| $MSSM\begin{matrix} +h\nu\\ \rightleftarrows\\ -\Delta\end{matrix}2MS·$ | [2a,b] |
| $MSSG+MS· \rightleftarrows MSSM + GS\boldsymbol{·}$ | [3a,b] |
| $MSSG+GS· \rightleftarrows GSSG + MS\boldsymbol{·}$ | [4a,b] |

**Exchange *via* self-association (proximity-control)**

| $2 MSSG\rightleftharpoons\left( MSSG \right)_{2}$ | [5] |
| --- | --- |
| ${(\mathrm{MSSG})}_{2}\underset{\to}{+h\nu}MSSG­MS·+GS·$ | [6] |
| $MSSG­MS·\longrightarrow MSSM+GS·$ | [7] |
| $\left( \mathrm{MSSG} \right)_{2}\underset{\to}{+2h\nu}{(MS·)}_{2}+2 GS·$ | [8] |
| ${(MS·)}_{2}\underset{\to}{-\Delta}MSSM$ | [9] |

**Exchange *via* autocatalytic templating (proximity-control)**

| $MSSG+MSSM\rightleftharpoons{MSSM­\left( MSSG \right)}_{2}$ | [10] |
| --- | --- |
| ${MSSM­\left( MSSG \right)}_{2}\underset{\to}{+h\nu}MSSM­MSSG­MS·+GS·$ | [11] |
| $MSSM­MSSG­MS·\longrightarrow2 MSSM+GS·$ | [12] |
| ${MSSM­\left( \mathrm{MSSG} \right)}_{2}\underset{\to}{+2h\nu}MSSM{­(MS·)}_{2}+2 GS·$ | [13] |
| ${MSSM­(MS·)}_{2}\underset{\to}{-\Delta}2 MSSM$ | [14] |

## Equation (S4): Rate of spontaneous dimer synthesis through diffusion-controlled radical substitution

In this mechanism, dimers (MSSM) are produced by the reaction of freely diffusing monomer radicals (MS**·**) with glutathione-protected monomers (MSSG) as shown in reaction [3a] (Scheme S1). Here, "MS" corresponds to a single foldameric sequence. The reaction rate for [3a] (*v_s,ch_*) satisfies eq. (S1),

| $v_{s,ch}=k_{ch}\left[ MS· \right][\mathrm{MSSG}]$ | (S1) |
| --- | --- |

where *k_ch_* is the rate constant for the radical substitution step. In radical chain reactions, [MS**·**] can be approximated with a quasi-steady state approach. At low conversions, MS**·** is produced preferentially by homolytic cleavage of the starting material MSSG. Therefore, the rate of formation is proportional to the light intensity (*k_I_I*) and [MSSG]. The chain-termination steps consuming MS**·** involve collisions with MS**·** and GS**·**. Again, the large excess of MSSG at low conversions affords the approximation that reaction [1a] is dominant. Thus, there is a 12 times molar excess for [GS**·**], that is, [GS**·**] ≈ 12 x [MS**·**]. Consequently, the chain-termination rate can be expressed as a second order function in [MS**·**] (eq. (S2)).

| $\frac{d[MS·]}{dt}=0=k_{I}I\left[ \mathrm{MSSG} \right]-k_{t}\left[ MS· \right]^{2}-k_{t}12\left[ MS· \right]^{2}$ | | (S2) |
| --- | --- | --- |
| $\left[ MS· \right]=\sqrt{\frac{k_{I}I}{13k_{t}}[\mathrm{MSSG}]}$ | (S3) | |

Here, *k_I_* is the rate constant of the light-induced homolytic cleavage, and *k_t_* is the rate constant for the chain-termination reaction. Substituting eq. (S3) into eq. (S1) and collecting the concentration- and light intensity-independent terms into the constant *s_ch_*, the rate is given by eq. (S4).

| $v_{s,ch}=s_{ch}\sqrt{I}{[\mathrm{MSSG}]}^{1.5}$ | (S4) |
| --- | --- |

## Equation (S8): Rate of dimer breakdown through diffusion-controlled radical substitution

The decomposition of MSSM proceeds in this mechanism through the reaction between the freely diffusing glutathione radical (GS**·**) and MSSM (reaction [3b]). The rate equation can be expressed as follows (eq. (S5)).

| $v_{b}=k_{b}\left[ GS· \right][\mathrm{MSSM}]$ | (S5) |
| --- | --- |

For [GS**·**], we apply the quasi-steady state approximation again. The source of [GS**·**] is the light-induced homolytic cleavage of MSSG and GSSG. The chain-termination steps consuming GS**·** involve collisions with GS**·** and MS**·**. Due to the 12 times excess of GS**·** over MS**·** in the system studied, the latter reaction can be neglected. Thus, we obtain eq. (S6).

| $\frac{d[GS·]}{dt}=0=k_{I}I(\left[ \mathrm{MSSG} \right]+2\left[ \mathrm{GSSG} \right])-k_{t}\left[ GS· \right]^{2}$ | (S6) |
| --- | --- |

Due to the mass balance for the ‘GS’ moiety, the term [MSSG] + 2[GSSG] is constant and equals to the initial concentration of MSSG ([MSSG]_0_), which yields eq. (S7).

| $\left[ \mathrm{GS}^{.} \right]=\sqrt{\frac{k_{I}I}{k_{t}}{[\mathrm{MSSG}]}_{0}}$ | (S7) |
| --- | --- |

Thus, [GS**·**] is proportional to the square root of the light intensity, and eq. (S7) can be substituted into (S5). Collecting the constant term into the overall rate constant of *b*, we obtain eq. (S8).

| $v_{b}=b\sqrt{I}[\mathrm{MSSM}]$ | (S8) |
| --- | --- |

## Equations (S11) and (S12): Rates of dimer synthesis through proximity-controlled mechanisms

This mechanism begins with the association preequilibrium producing the complex (MSSG)_2_.

| $[{(\mathrm{MSSG})}_{2}]=\frac{1}{K_{D,p}}{[\mathrm{MSSG}]}^{2}$ | (S9) |
| --- | --- |

*K_D,p_* stands for the dissociation constant. The rate-determining step is the photochemical cleavage of a monomer within the complex (reaction [6]). If the geometry of the complex is advantageous, the resulting high-energy radical can rapidly relax through the intracomplex radical substitution [7]. The rate of formation for MSSG-MS**^.^** is proportional to the light intensity (*k_I_I*) and [(MSSG)_2_]. Using eq. (S9), the rate equation can be obtained (eq. (S10)).

| $v_{s,p1}=\frac{k_{I}I}{K_{D,p}}{[\mathrm{MSSG}]}^{2}$ | (S10) |
| --- | --- |

Substituting the constant term with the overall rate constant *s_p1_* yields eq. (S11).

| $v_{s,p1}=s_{p1}{I[\mathrm{MSSG}]}^{2}$ | (S11) |
| --- | --- |

Literature results showed that recombination of two thiyl radicals is a possible photochemical exchange mechanism, when radical substitution is sterically hindered^[2]^. Therefore, the intracomplex version of this mechanism cannot be ruled out *a priori*. If the absorption of the two photons is consecutive, the reaction rate remains first order in light-intensity; that is, the functional form of the rate equation is the same as eq. (S11). If coincident absorption of two photons produces the diradical intermediate (MS**·**)_2_ and MSSM is formed in a concerted manner (steps [8] and [9]), the probability of the interaction with two photons is proportional to the square of the light intensity.

The cross-section of the interaction is not decreased by the non-linear “two-photon absorption” effect, because disulfides in the complex are not coupled quantum mechanically. Moreover, the recombination of the diradical [9] does not involve any activation energy, leading to a very fast reaction. On this ground, we incorporated this mechanism into the model with the rate equation (S12).

| $v_{s,p2}=s_{p2}{I^{2}[\mathrm{MSSG}]}^{2}$ | (S12) |
| --- | --- |

## Equations (S14) and (S15): Rates of autocatalytic dimer synthesis

The autocatalytic mechanism starts with a binding preequilibrium between the dimers and the monomers [10]. The dissociation constant (*K_D,a_*) determines the concentration of the complex available for the further, rate determining step.

| $[\mathrm{MSSM}\text{-}{(\mathrm{MSSG})}_{2}]=\frac{1}{K_{D,a}}{[\mathrm{MSSM}][\mathrm{MSSG}]}^{2}$ | (S13) |
| --- | --- |

The calculation of the rates for the autocatalytic routes is closely analogous to the non-autocatalytic synthesis pathways described above. For the intracomplex radical-substitution pathway, the rate-determining step is the absorption of a photon, which leads to a linear light-intensity dependence of the reaction rate. Using (S13), eq. (S14) is obtained.

| $v_{s,a1}=s_{a1}I{[\mathrm{MSSM}][\mathrm{MSSG}]}^{2}$ | (S14) |
| --- | --- |

Coincident absorption and the concerted conversion to MSSM is also possible for the autocatalytic complex, which yields a rate equation quadratic in light intensity (eq (S15)).

| $v_{s,a2}=s_{a2}I^{2}{[\mathrm{MSSM}][\mathrm{MSSG}]}^{2}$ | (S15) |
| --- | --- |

## Dynamic model for the foldamer-based photochemical disulfide exchange system

The differential equation describing the time- and light intensity-dependent concentration of the dimers ([MSSM]) (eq. S16) contained the following rate terms: non-autocatalytic (spontaneous proximity-controlled) synthesis (*v_s,p1_* and *v_s,p2_*), autocatalytic synthesis (*v_s_,_a1_* and *v_s,a2_*) and breakdown (*v_b_*).

| $\frac{d[\mathrm{MSSM}]}{dt}=v_{s,p1}+v_{s,p2}+v_{s,a1}+v_{s,a2}-v_{b}$ | (S16) |
| --- | --- |

## Fitting the dynamic model to the experimental time- and energy-dependent data arrays

Differential equation (S16) was numerically integrated using the method LSODA (Livermore Solver for Ordinary Differential Equations with Automatic method switching for stiff and nonstiff problems)^[5]^ to simulate the light intensity-dependent time evolution of a dimer. The synthesis rates are dependent on the actual monomer concentration, which was expressed with [MSSM] using the mass balance (S17).

| $\left[ \mathrm{MSSG} \right]=\left[ \mathrm{MSSG} \right]_{0}-2\left[ \mathrm{MSSM} \right]$ | (S17) |
| --- | --- |

The numeric integrations were carried out with the parameters of [MSSM]_0_ = 0, [MSSG]_0_ = 2x10^-5^ M. The light intensities were set to the calibrated values. The rate constants *s_p1_*, *s_p2_*_,_ *s_a1_*, *s_a2_* and *b* were determined by weighted least-squares fitting to the experimental concentration data using least_squares_fitting procedure from the scipy^[3]^ Python package with a trust-region reflective algorithm^[4]^.

## ITC characterization of the binding of foldameric dimers to CaM

To quantitatively characterize the interaction between the foldameric dimers and the seeding protein, two of the best ligands (**WF-RW** and **WW-RF**) were selected as representatives from the dissipative selection experiment. Fragments of the dimers were coupled together through a chemically stable thioether linkage (hereinafter referred to as **WF-S-RW** and **WW-S-RF**). Both dimers displayed two-step binding to CaM (Figure S10). First, a high-affinity step was found with a K_D_ of 1.76 ± 0.65 nM (n = 1.02 ± 0.03) for **WF-S-RW** and 8.74 ± 4.03 nM (n = 1.01 ± 0.05) for **WW-S-RF**. The 1:1 stoichiometry in both cases strongly suggested that the two lobes of the protein with separate binding sites were involved in the binding of a single foldameric dimer. Second, a lower affinity step with a fractional stoichiometry was also detected for both dimers (Figure S10a and S10c). This pointed towards, that the different helix segments of the dimers could separately interact with both lobes of CaM, which led to crosslinking of the protein by the ligand at micromolar concentrations^[8]^. In order to confirm that the foldameric ligands recognize the orthosteric interface of the template, competitive ITC experiments were carried out with TRPV1-CT_15_, the native ligand of CaM, as described previously^[8]^. CaM: foldameric dimer 1:2 sample was titrated with TRPV1-CT_15_ during which no binding events were found.

# Supplementary Figures

**
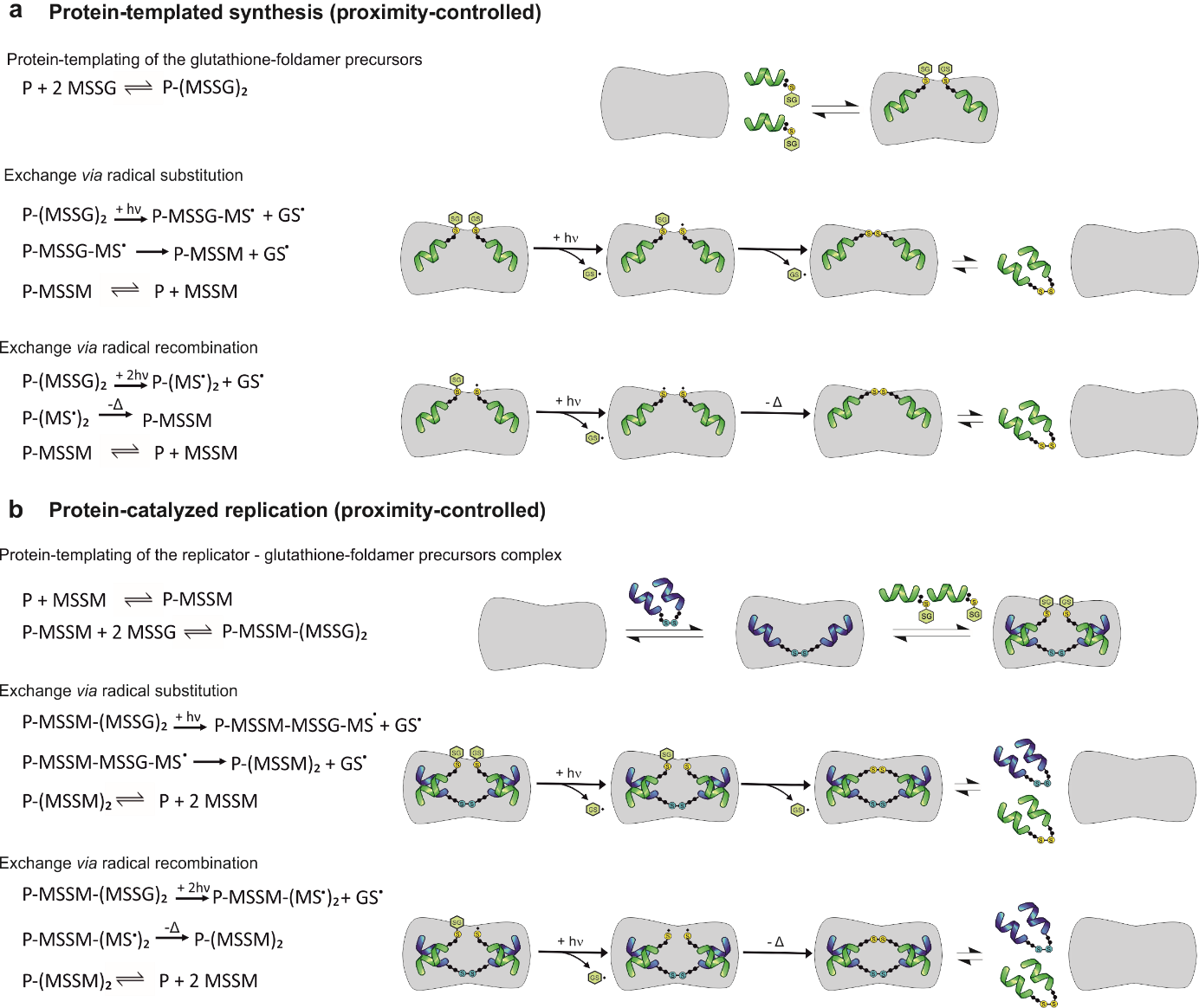
**

Figure S1. Schematic representation of protein-induced photochemical disulfide exchange reactions. (**a**) Complex formation between the protein and the helical monomers and subsequent proximity-controlled radical substitution or concerted metathesis. (**b**) Proposed mechanism for templated dimer formation within the protein’s environment. Protein templates dimer and precursor monomers simultaneously facilitating the proximity-controlled radical substitution or concerted metathesis.

**
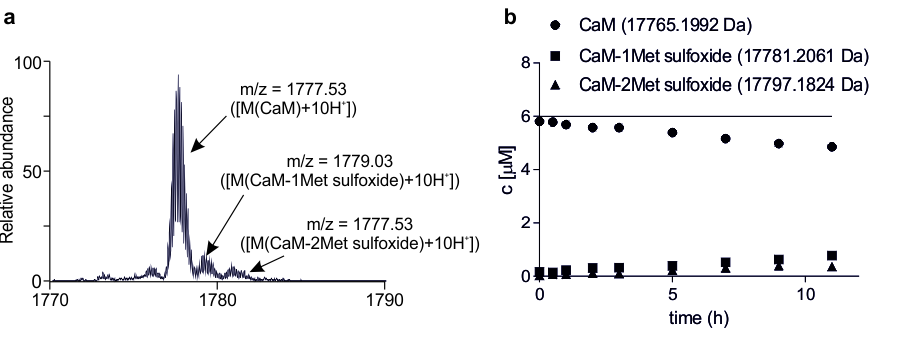
**

Figure S2. Monitoring the UV-induced degradation of CaM**.** CaM in a concentration of 6 μM was irradiated with UV light (365 nm, 5.1 mW cm^-2^) for 11 h (**a**) extracted MS spectrum of CaM after 5 h irradiation showed appearance of oxidized protein, (**b**) time dependence of the concentration of the intact CaM (circle) and the oxidized species (square and triangle).


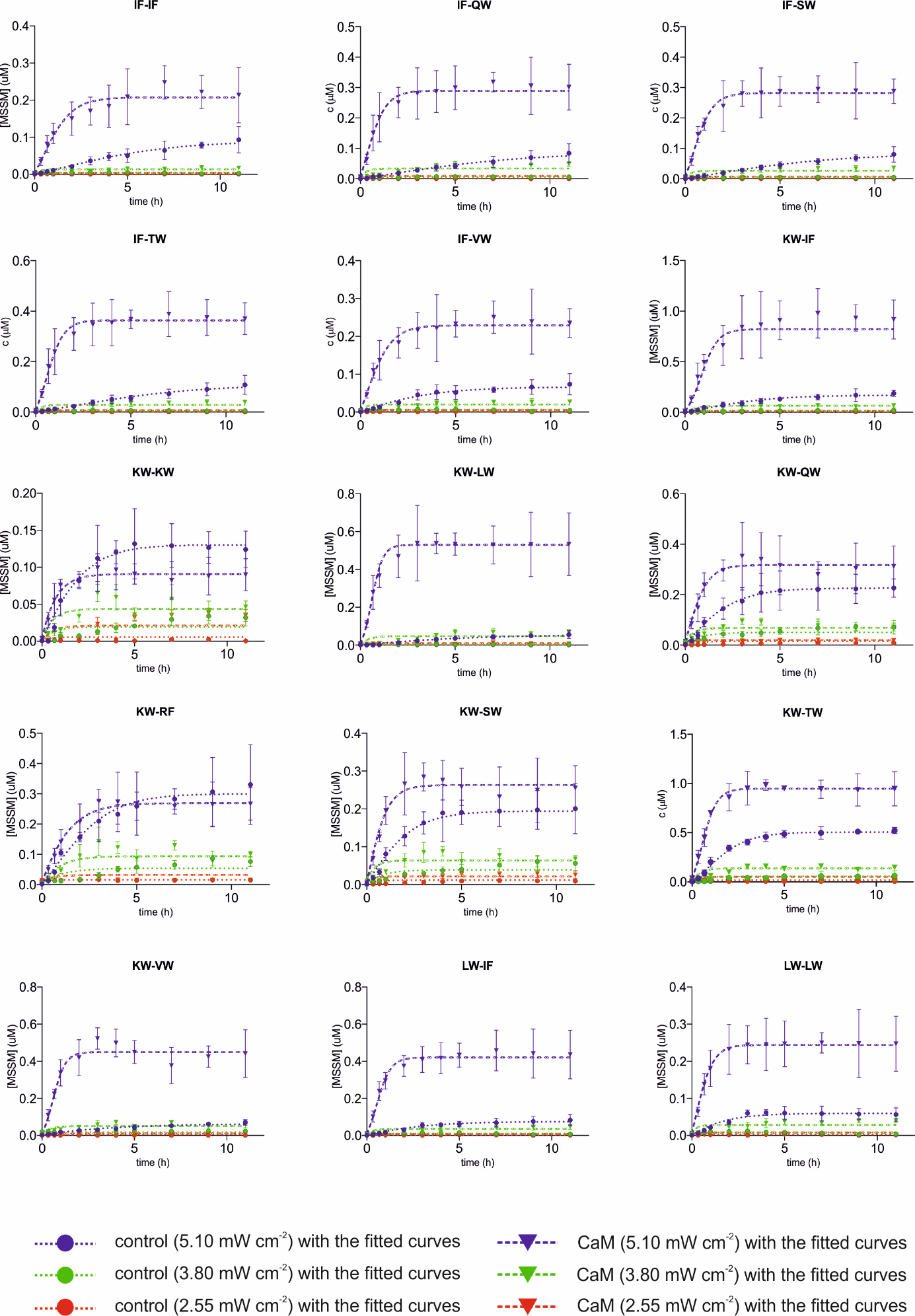

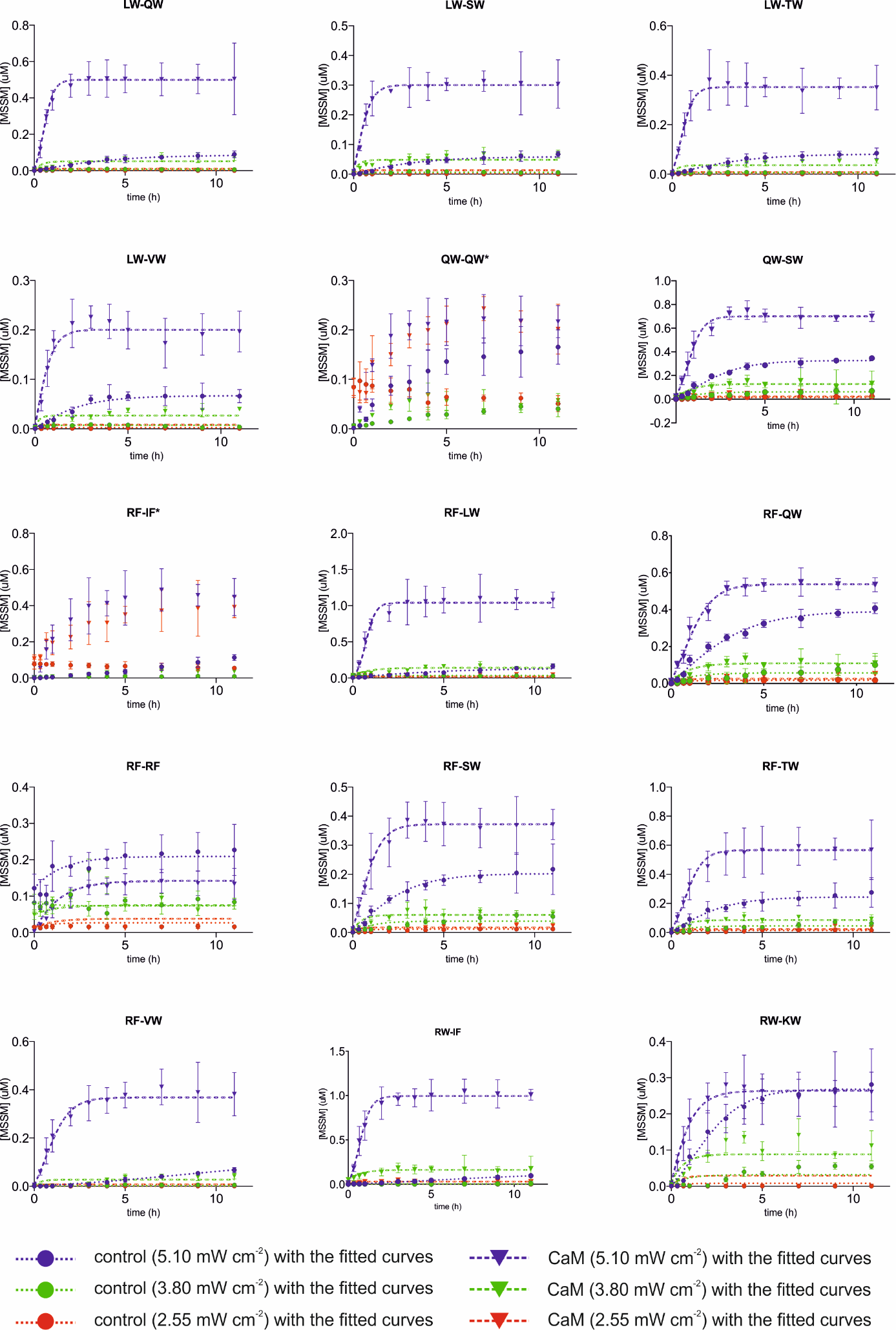


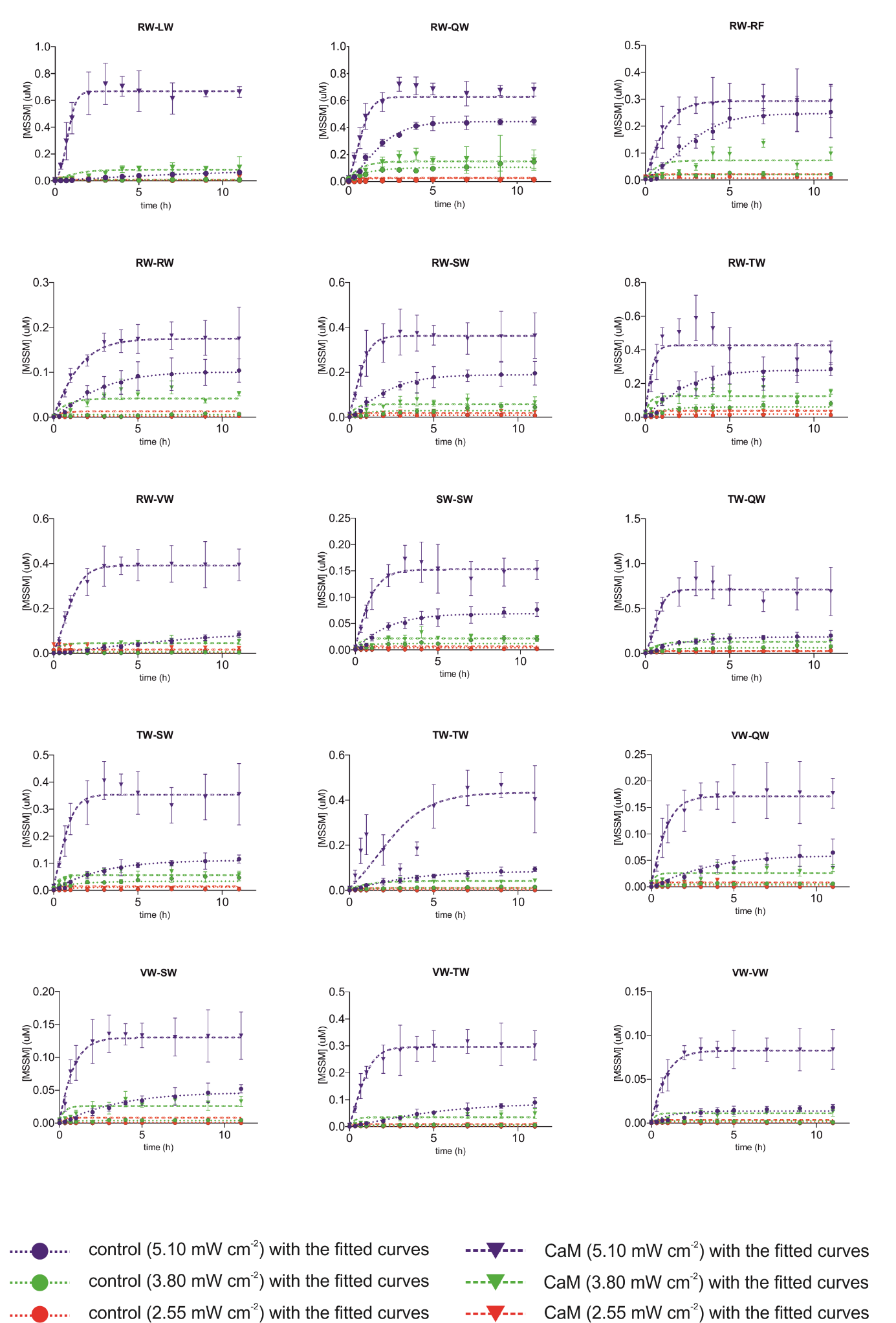


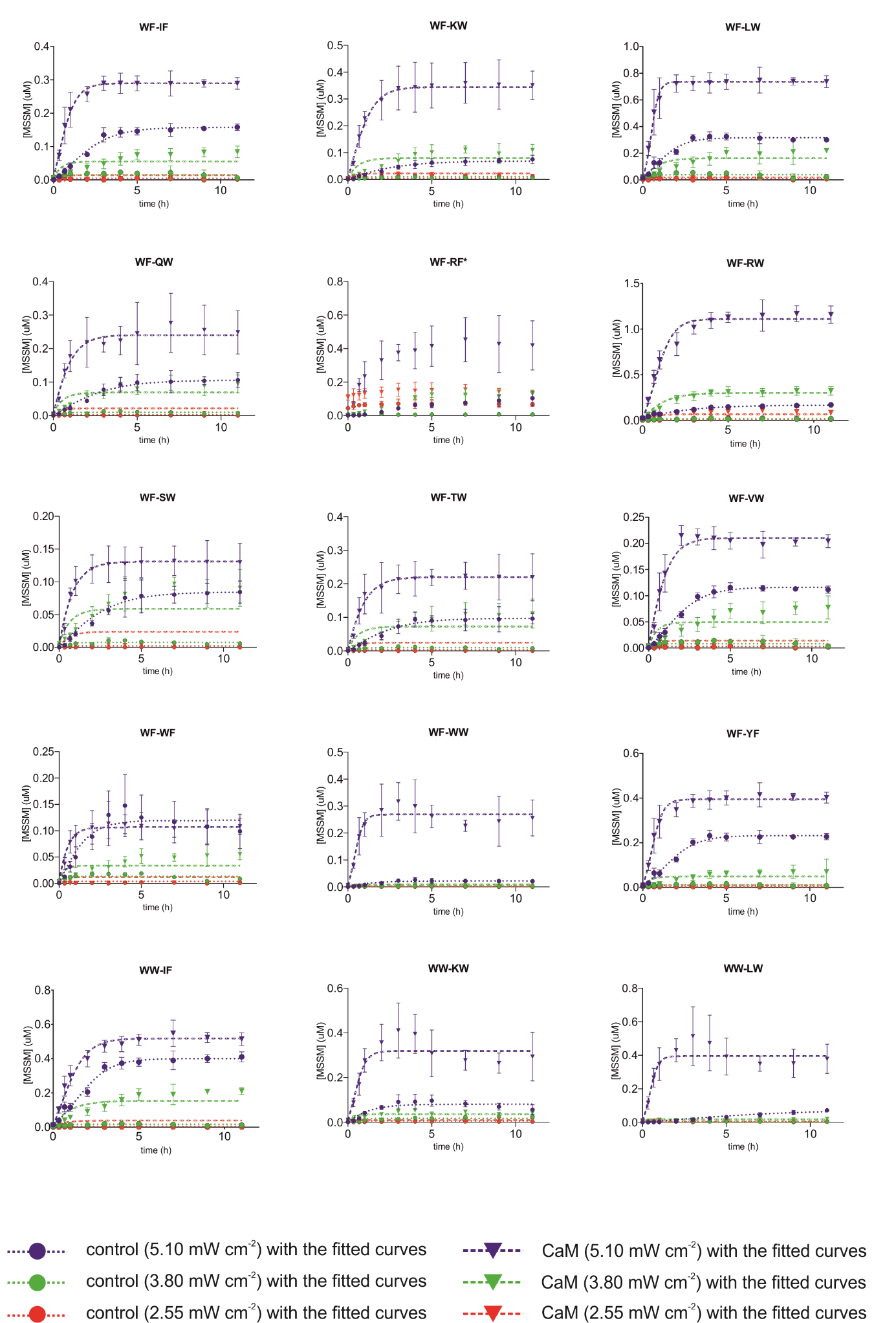


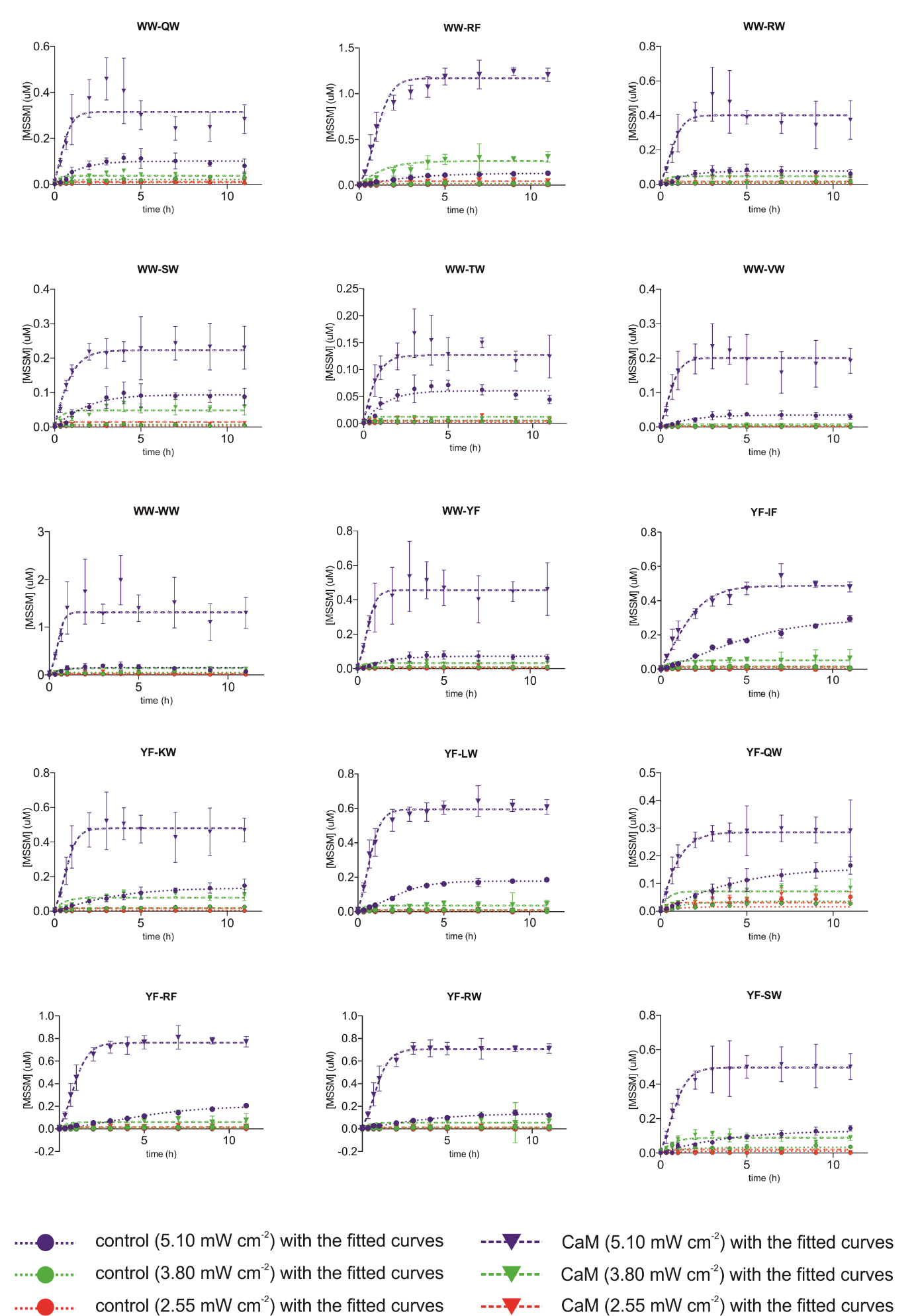

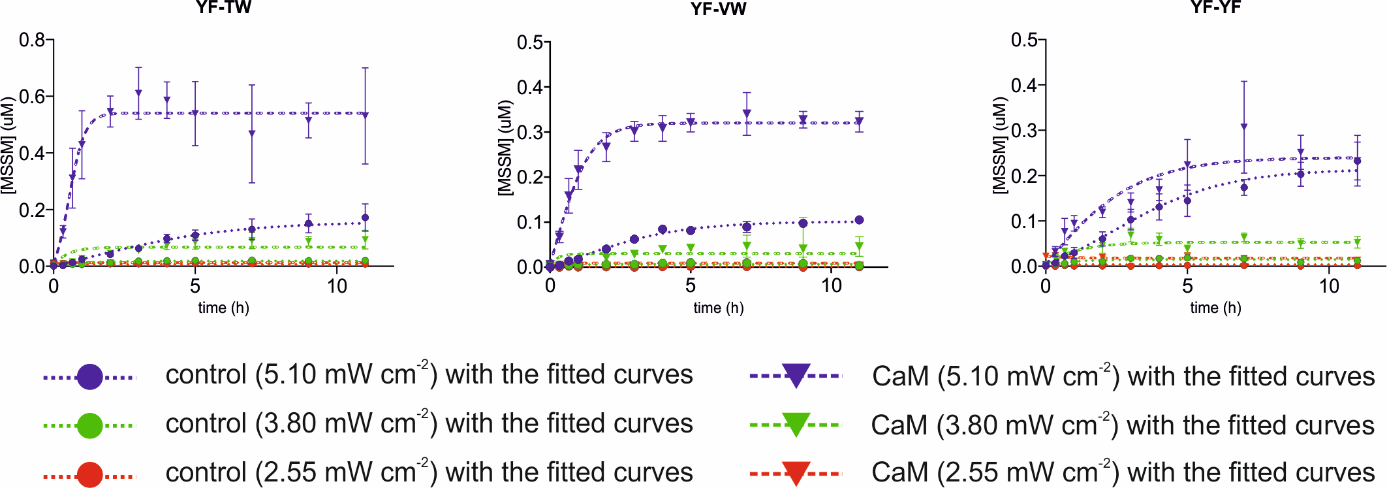


Figure S3 Fitting of the dynamic model of chemical evolution to experimental data for representative replicators**.** Replicator concentration vs. time measured for the pure foldamer system (circle) and for the protein-catalyzed system (triangle). Data at different light intensities are represented with different color as follows: 2.55 mW cm^-2^ (red), 3.80 mW cm^-2^ (green), 5.10 mW cm^-2^ (blue); fitted curves are depicted with matching color. Non-linear least square analysis was used for fitting. Experimental data were obtained from three parallel measurements carried out on three different samples. See Supplementary Tables 3 and 4 for fitted parameters. Dimers marked with an asterisk (**QW-QW**, **RF-IF**, **WF-RF**) were co-eluted with CaM, which has isotopic peaks matching to [M+H]^+^ or [M+2H]^2+^ peaks of the compounds. Since the protein had significant influence on the AUC especially when the concentration of the dimer was low, these dimers were omitted from the fitting.

Figure S4. Effect of autocatalysis on protein-induced dimer formation. Nonlinear regression of the dynamic model to the time- and light intensity-dependent data. Best-fitting dynamic models with (solid) and without (dashed curves) autocatalysis. Representative experimental time- and light intensity-dependent data array is shown for the dimer **WF-YF** measured at power densities of 2.55 mW cm^−2^ (gray), 3.80 mW cm^−2^ (green), and 5.10 mW cm^−2^ (blue), The concentration of CaM was constant at 6 µM.


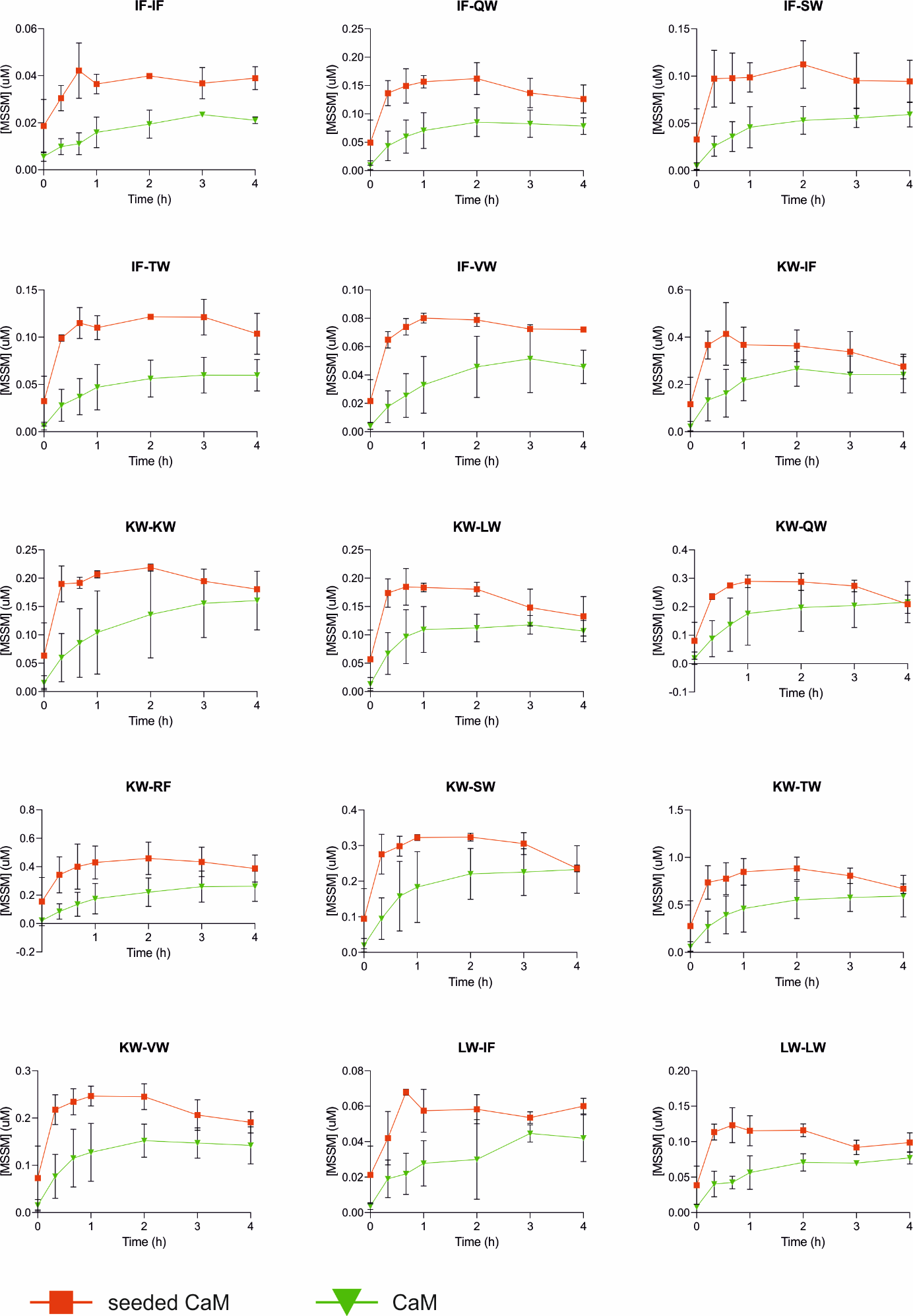


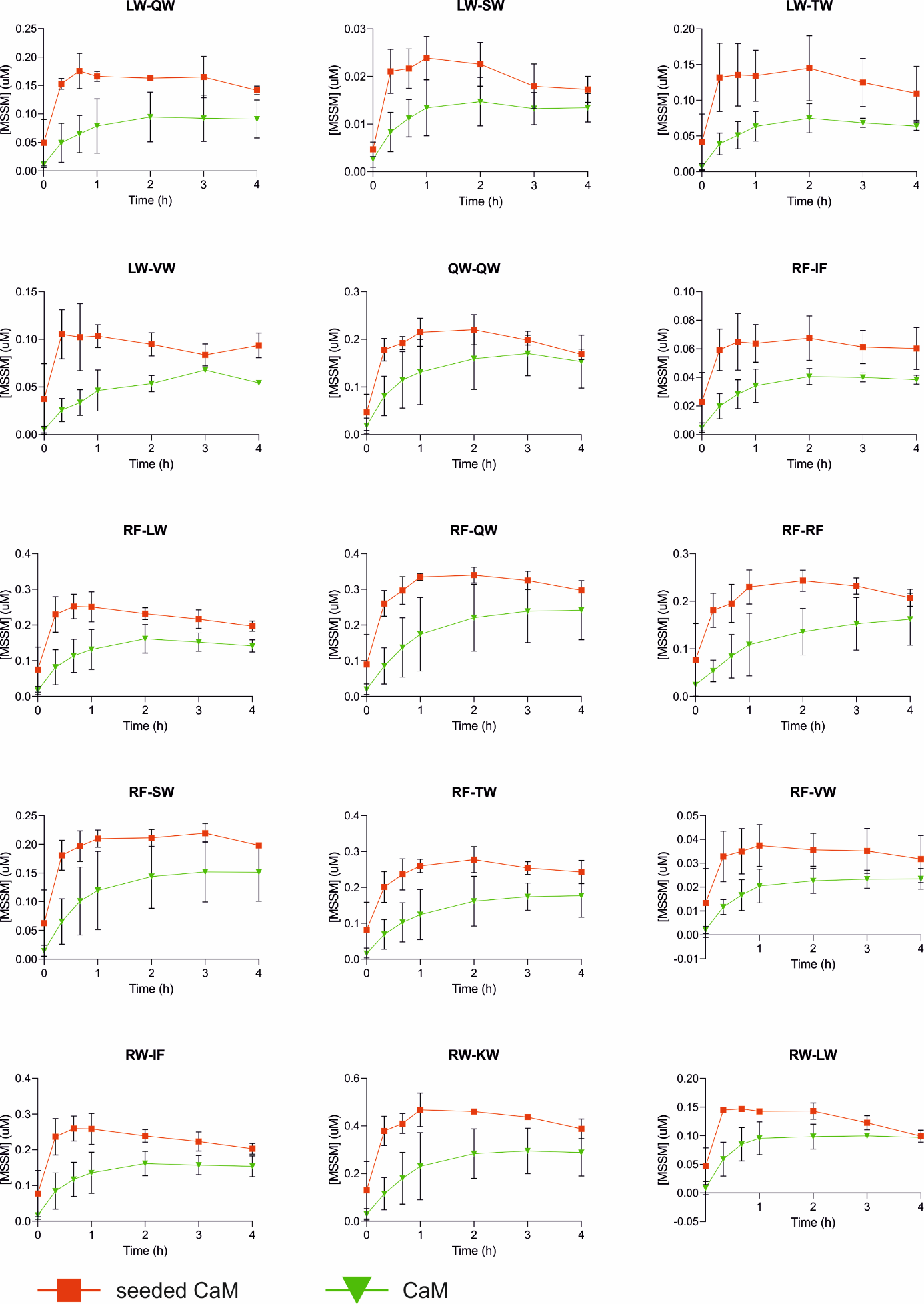


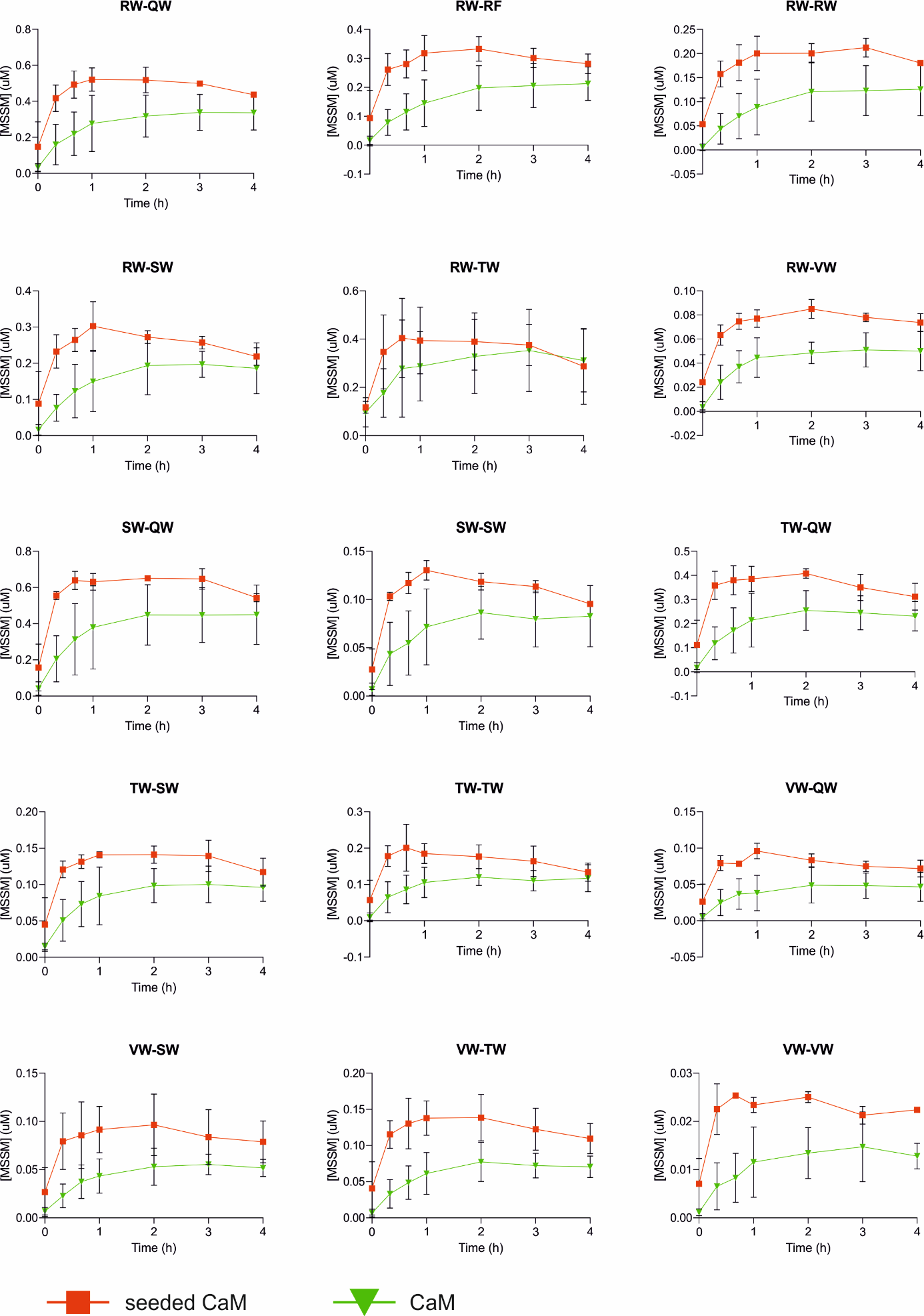


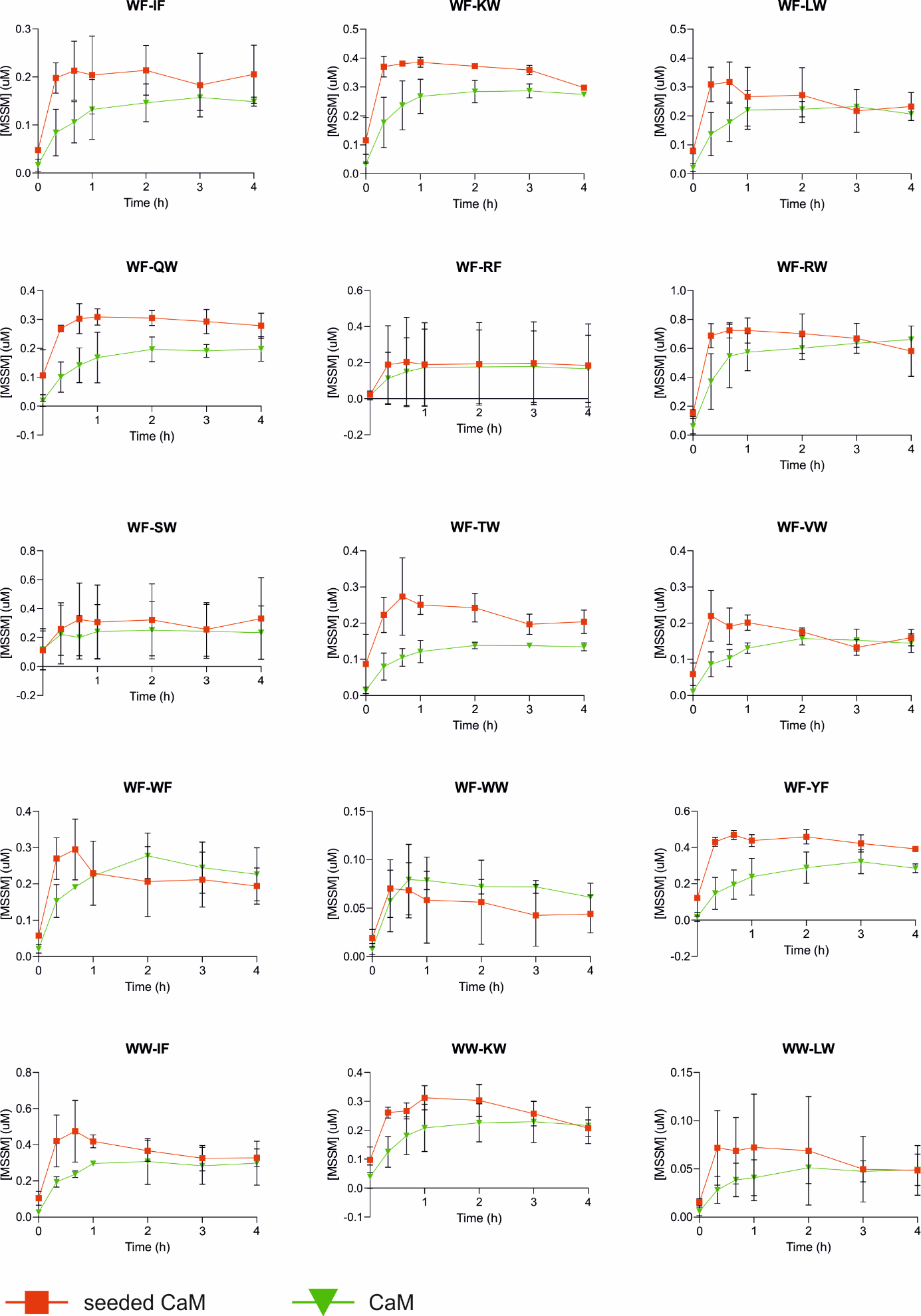


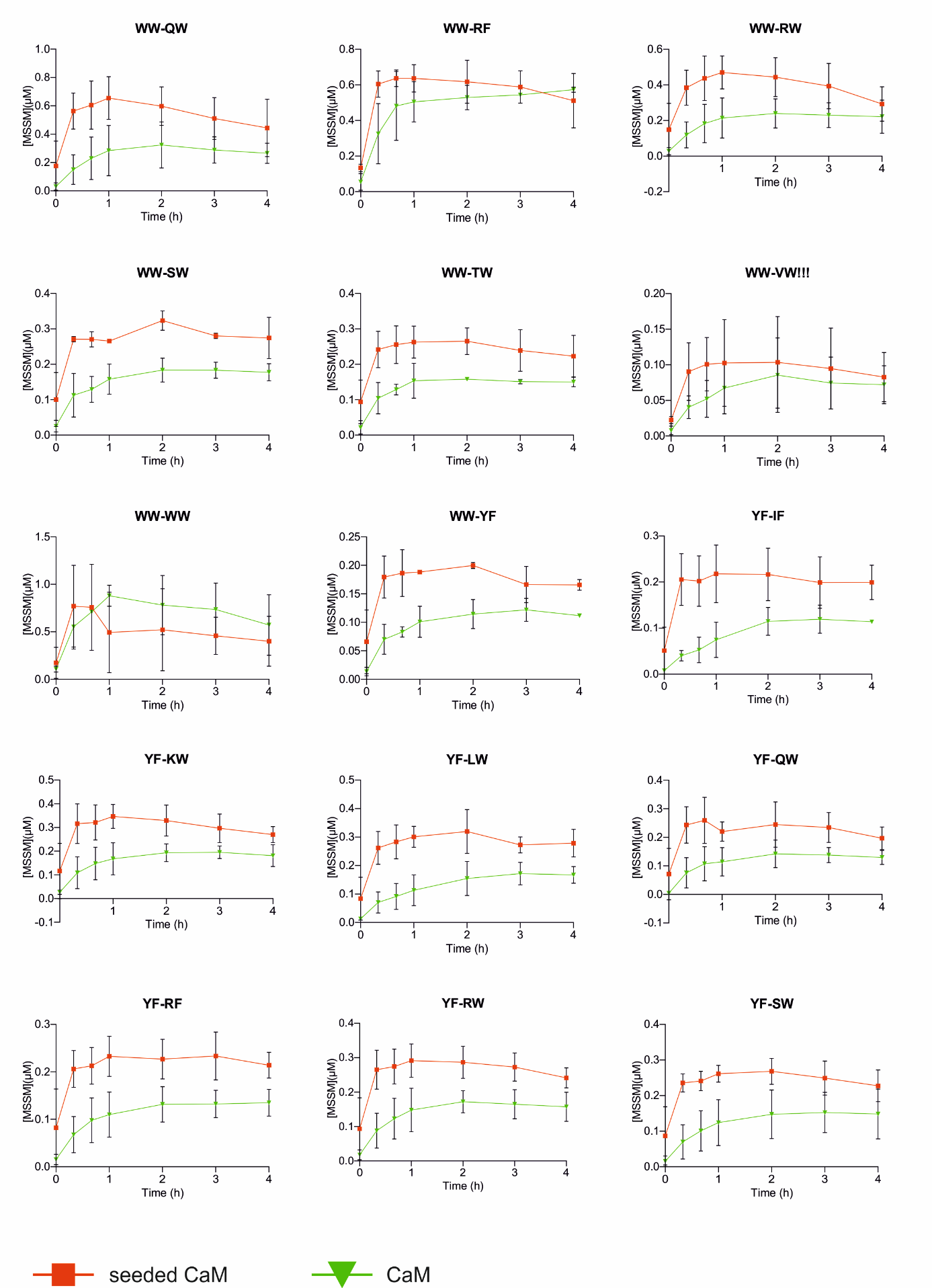


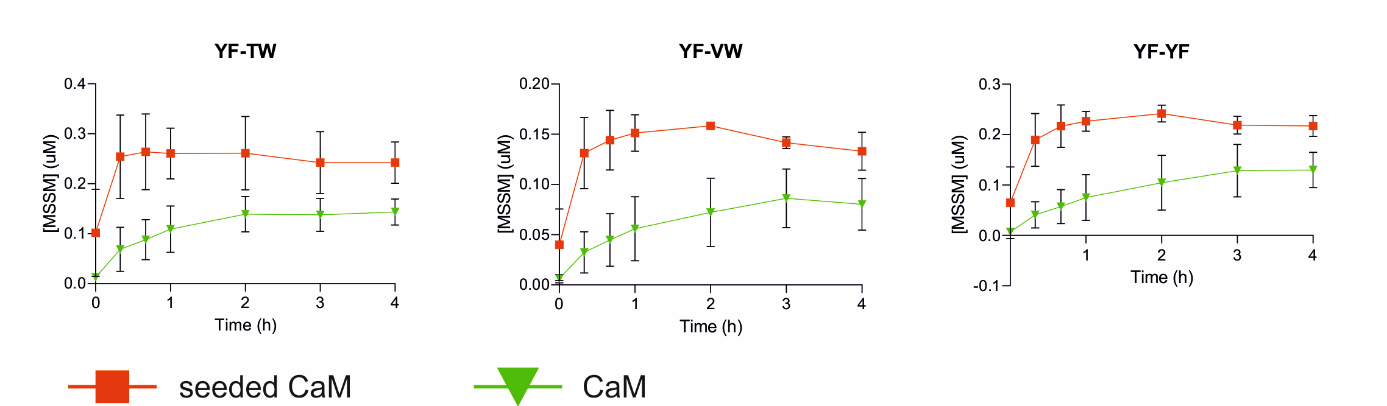


Figure S5. Results of the seeding experiments. Time-dependent data array for representative replicators in the protein template sample with seeding (red square) and without seeding (green triangle). Seeding was performed with a pre-irradiated mixture of glutathione protected monomers (initial mixture) with CaM.


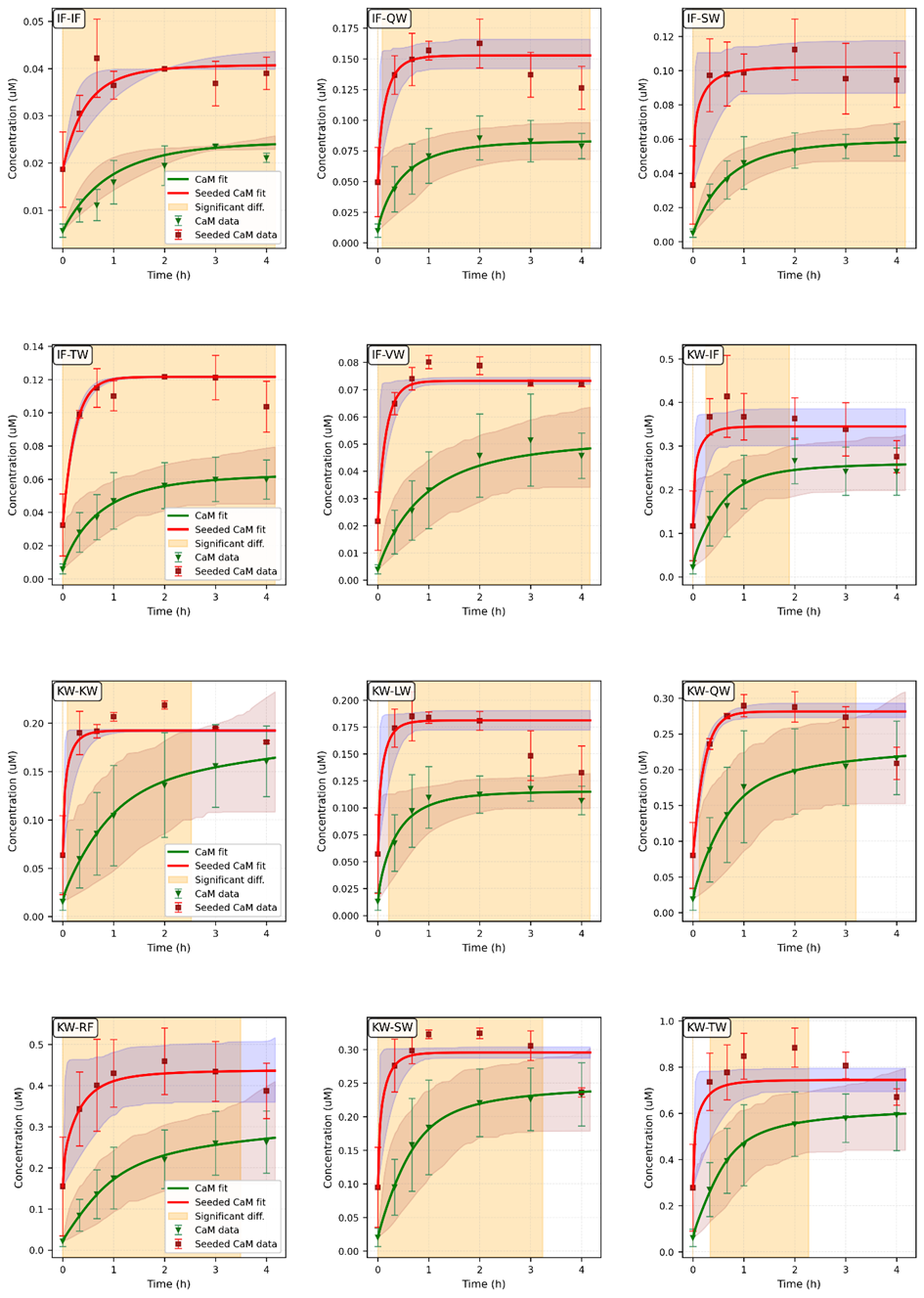


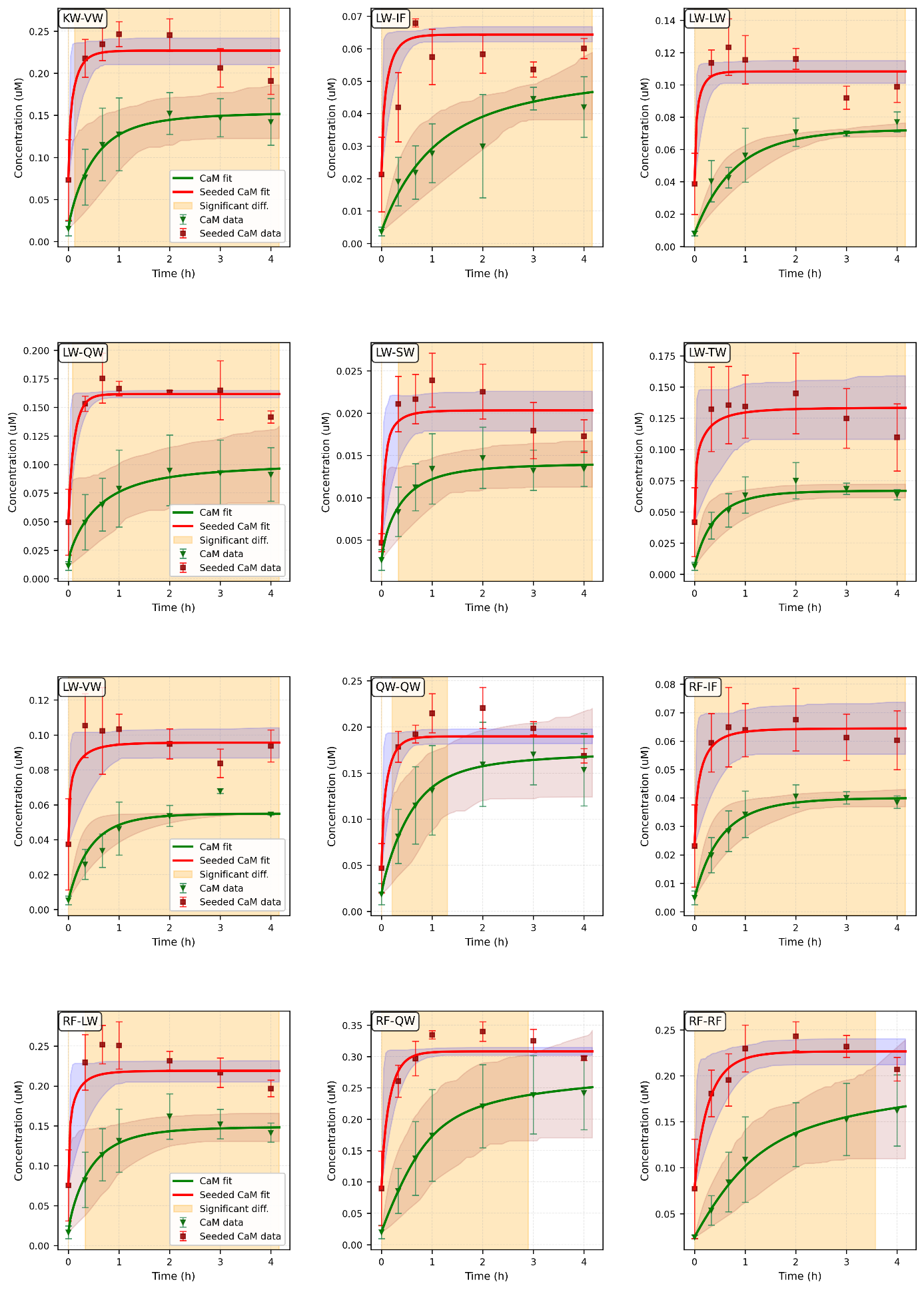


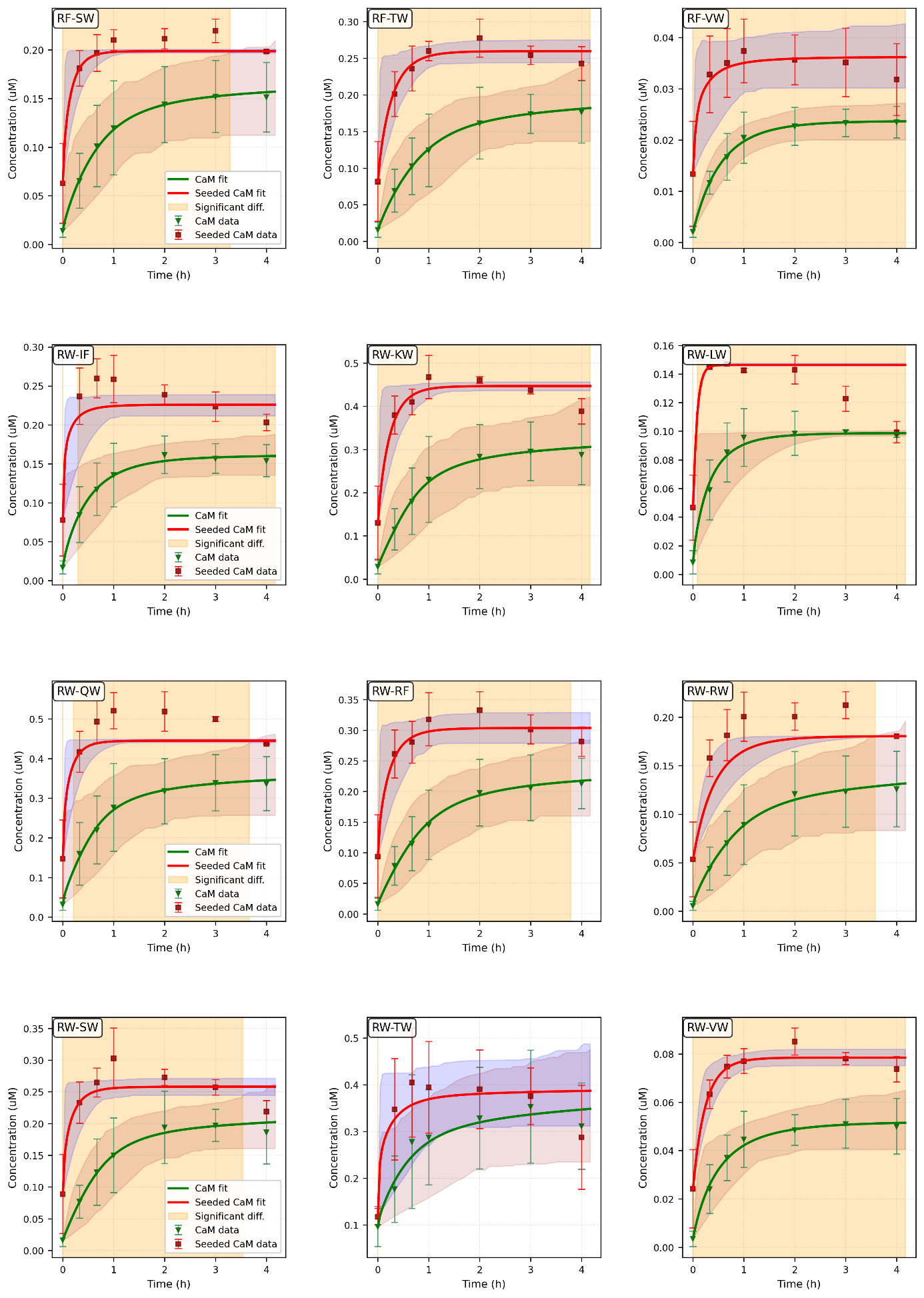


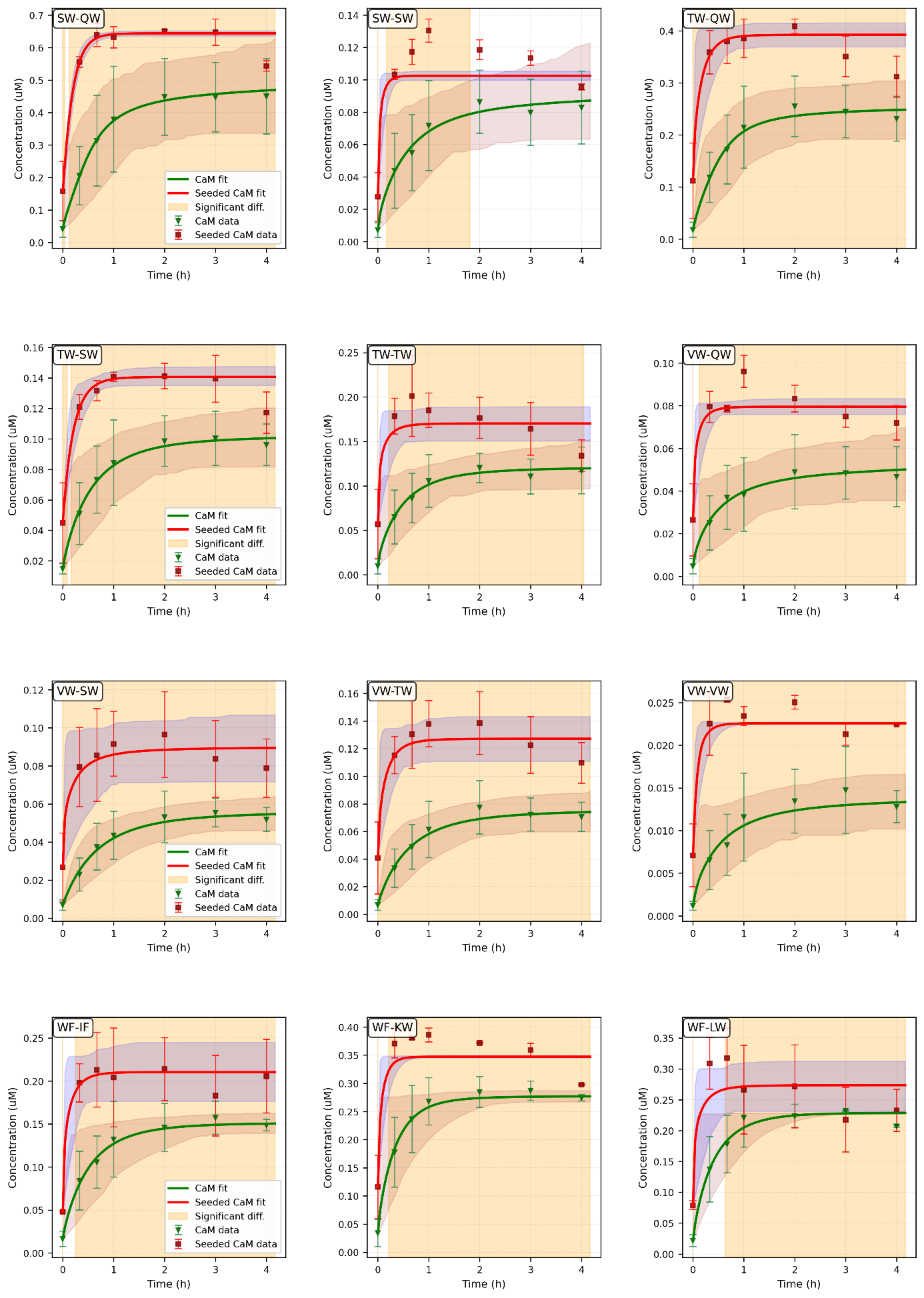


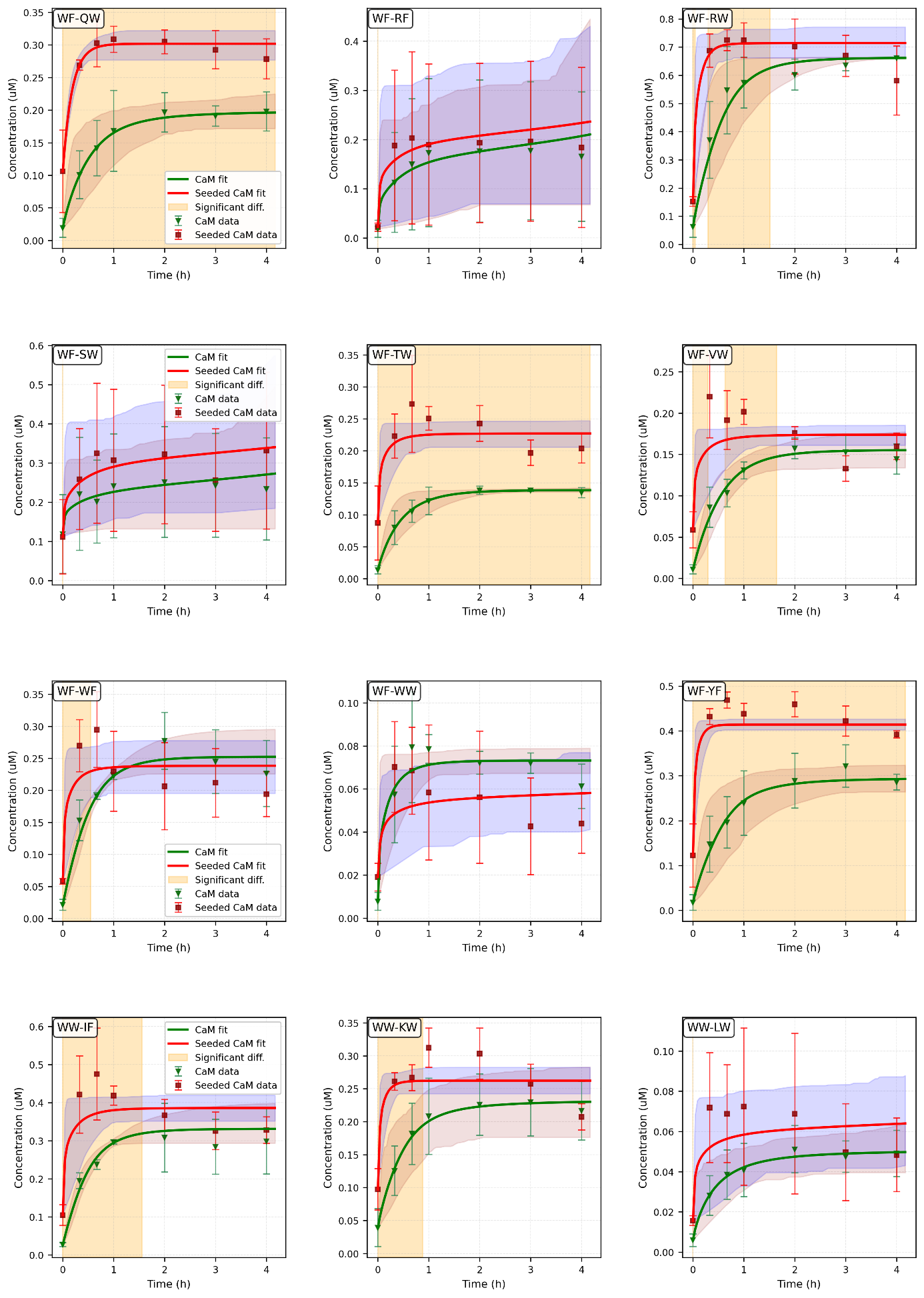


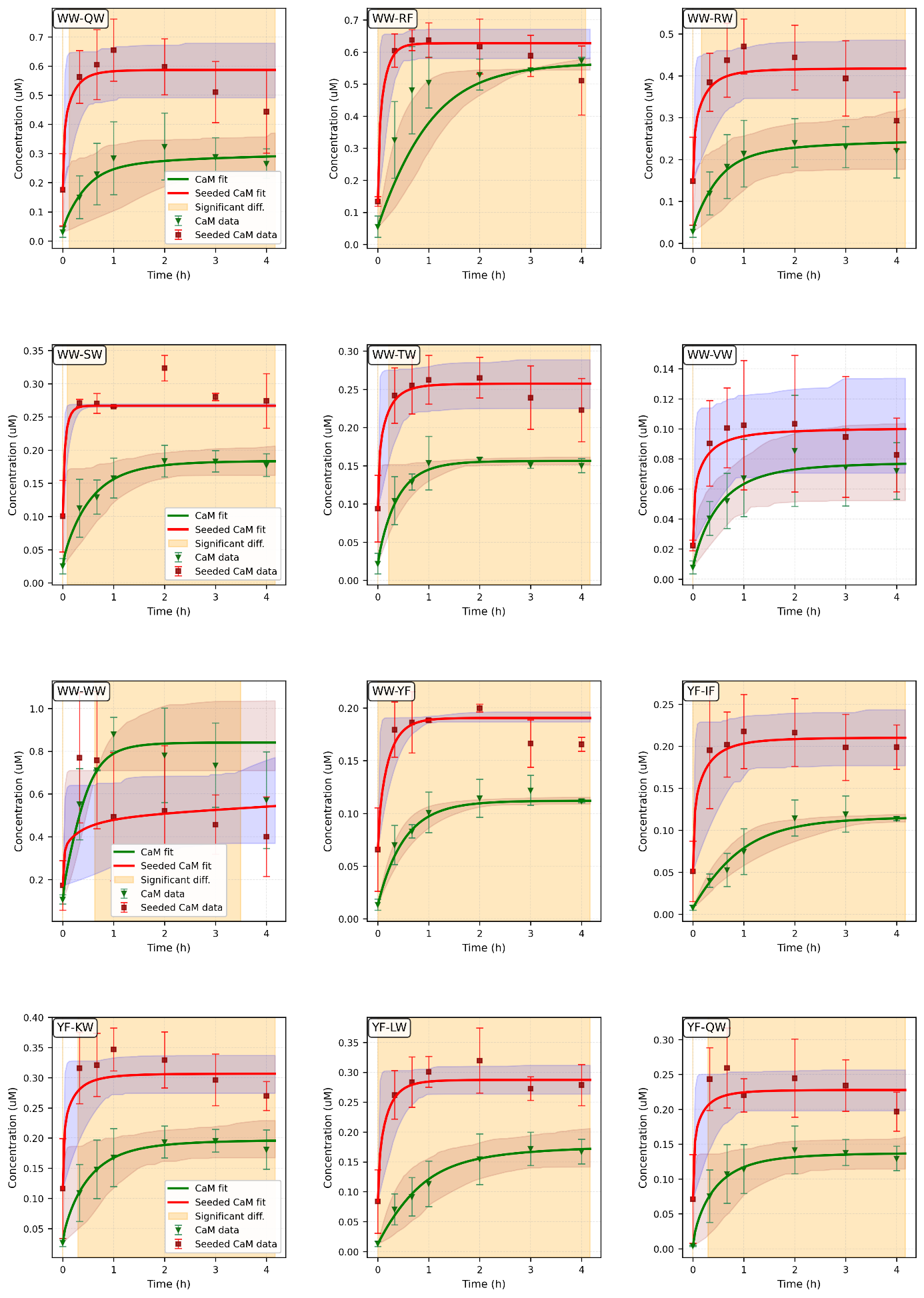


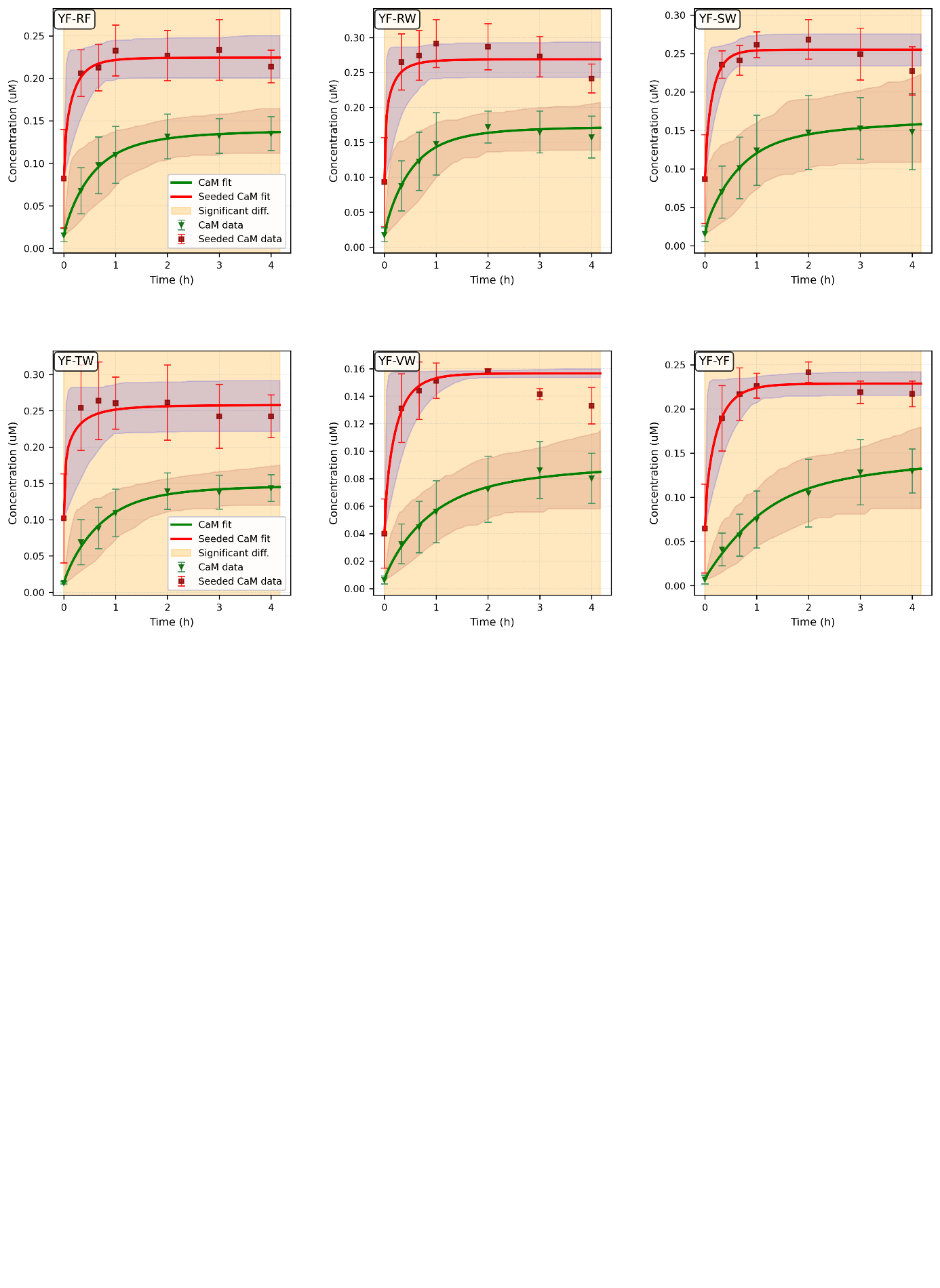


**Figure S6.**  **Comparison of kinetic model fits to the time-dependent concentration data for representative replicators in the seeding experiments.** Protein-templated samples with seeding (red squares) and without seeding (green triangles) are shown together with their corresponding 95% confidence intervals (blue for seeded, brown for non-seeded). Regions in which the confidence intervals do not overlap—indicating statistically significant differences between the two conditions—are highlighted with an orange background.


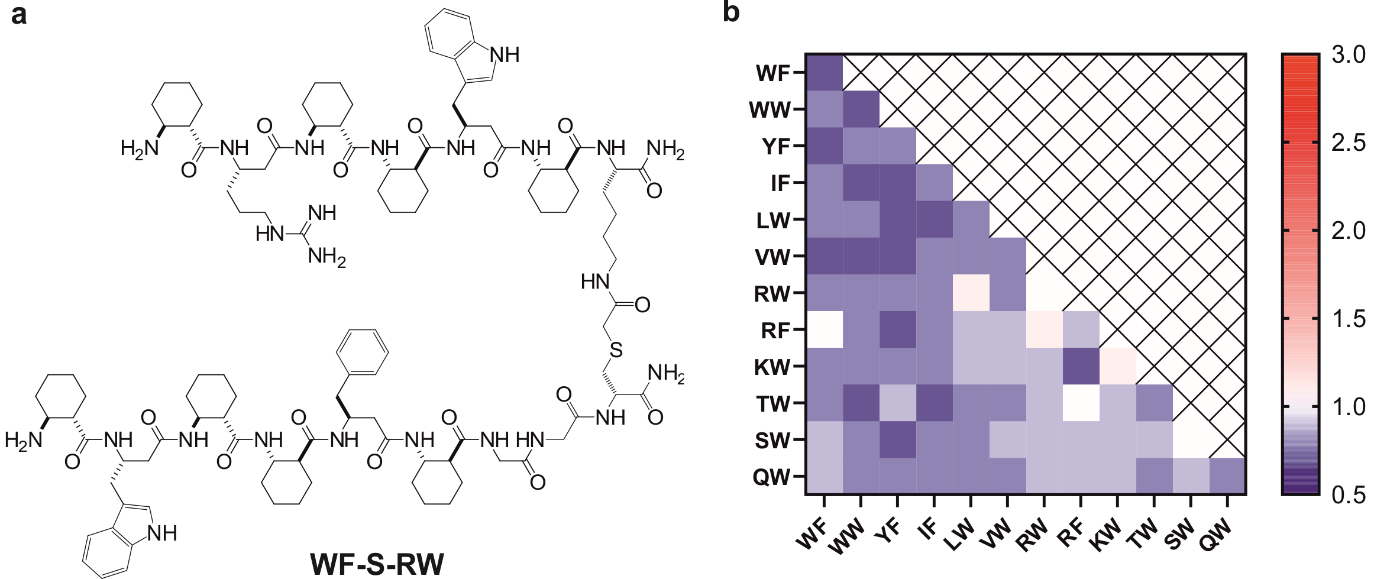


Figure S7. Seeding effect of WF-S-RF on protein-free foldamer system. (**a**) Structure of the **WF-S-RW** dimer with the light-insensitive thioether linkage. (**b**) Heat map representation of the sequence-dependent initial rate increase (ν_seeded_/v_control_) upon seeding the protein-free foldameric mixture with **WF-S-RW.** The concentration of the seed was 10 μM and each precursor monomer was applied at 10 μM concentration. Light intensity was 5.10 mW cm^−2^ during the experiment^[1]^.


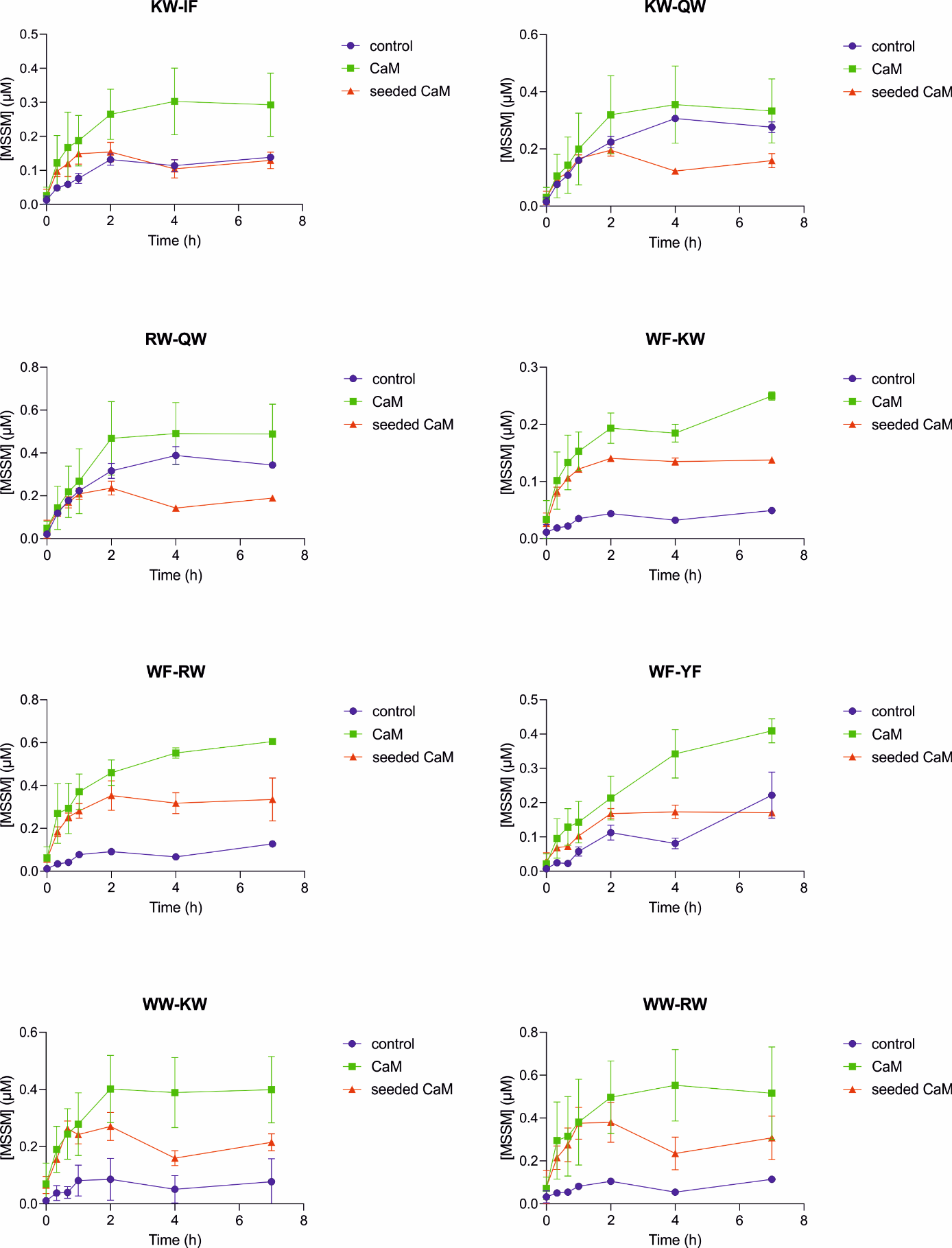


Figure S8. Time-dependent data array for representative replicators in a seeding experiment. Light insensitive dimer (**WF-S-RW**) was applied in a concentration of 6 μM (equimolar to CaM) as a seeding reagent. Concentration of dimer vs. time measured for the pure foldamer system: control (blue circle), for the protein-catalyzed system (green square) and for the seeded sample in the presence of CaM (red triangle).


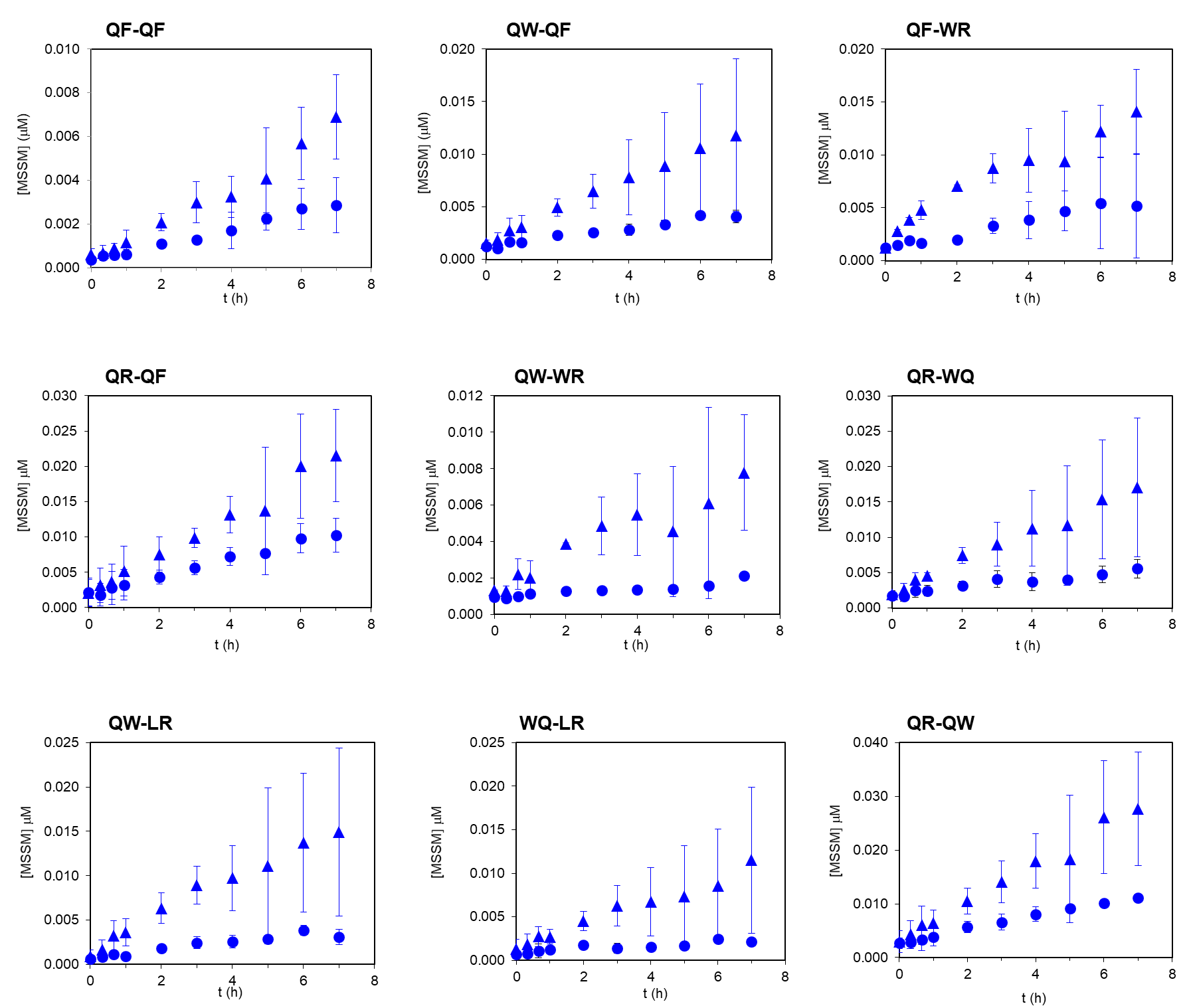


Figure S9. Results of the UV-light-driven disulfide exchange reaction in the presence of K-Ras G12D. Time-dependent concentration of representative foldamer dimers in the control sample (circle) and in the K-Ras G12D templated system (triangle) irradiated at a light intensity of 5.10 mW cm^-2^.Concentration of the other dimers were below the detection limit.

**
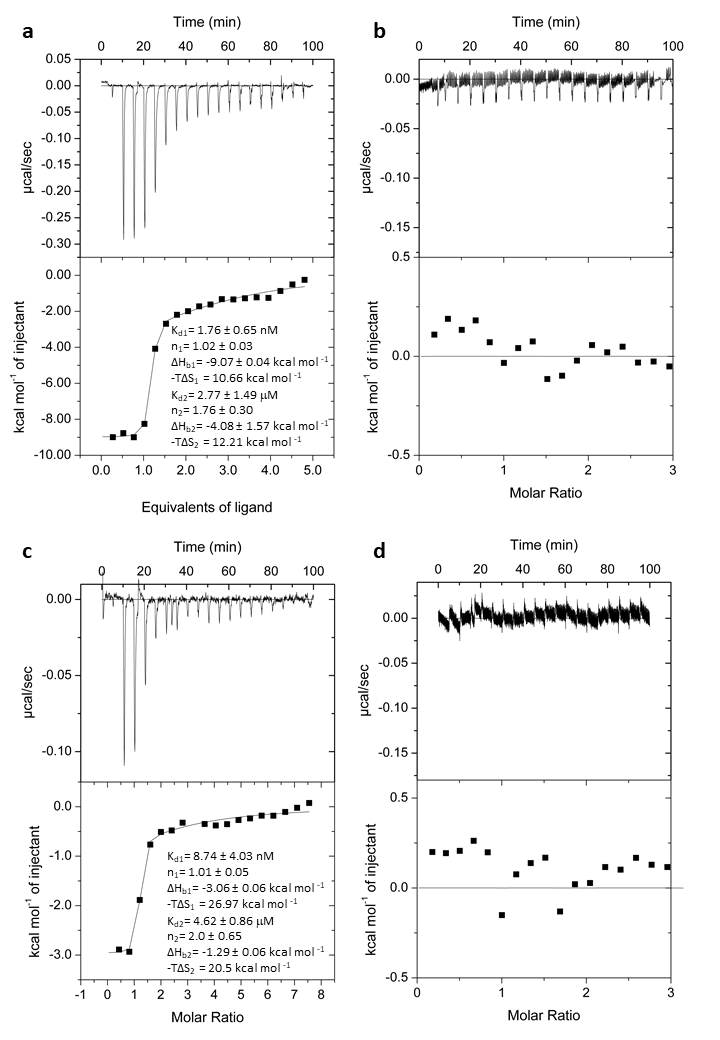
**

Figure S10. ITC competitive and control titrations for WF-S-RW and WW-S-RW ligands. Raw data (upper) and integrated peaks with fitted values (lower) with Kd, stoichiometry and thermodynamic parameters of the binding. TRPV1-CT_15_ was used as a competitor^[6]^ (**a**) **WF-S-RW** titrated to 3 μM CaM in the cell; (**b**) TRPV1-CT_15_ titrated to CaM:**WF-S-RW** 1:2 sample; (**c**) **WW-S-RF** titrated to 3 μM CaM in the cell; (**d**) TRPV1-CT_15_ titrated to CaM:**WW-S-RF** 1:2 sample. All titrations were performed in pH 7.4 20 mM HEPES buffer, 150 mM NaCl with 5 mM CaCl_2_, at 303 K.


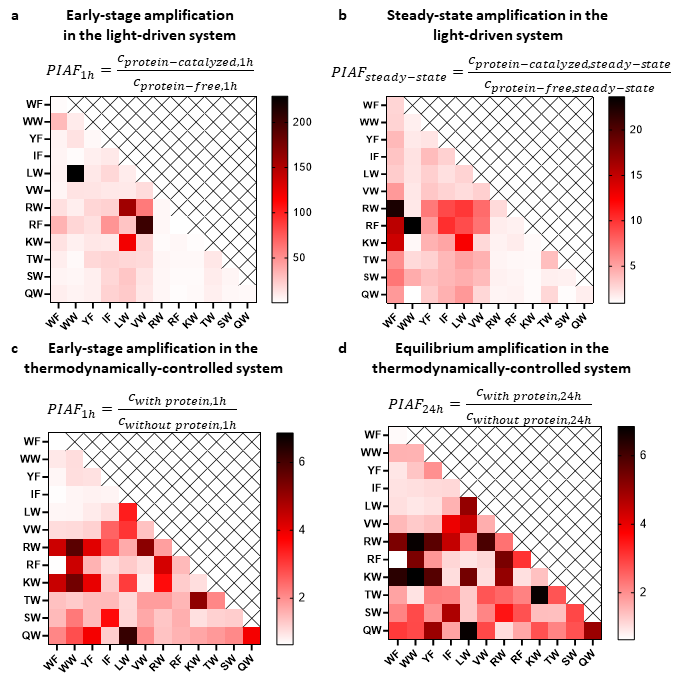


Figure S11. Evolution of replicator population in time. Comparison of the PIAF values obtained at an early stage of the reaction and at steady-state. Calculation and heatmap representation of PIAF values at 1 h (a) and the steady-state (b) in the light-driven samples**.** Calculation and heatmap representation of PIAF values at 1 h (c) and in equilibrium (d) in the thermodynamically-controlled system**.**


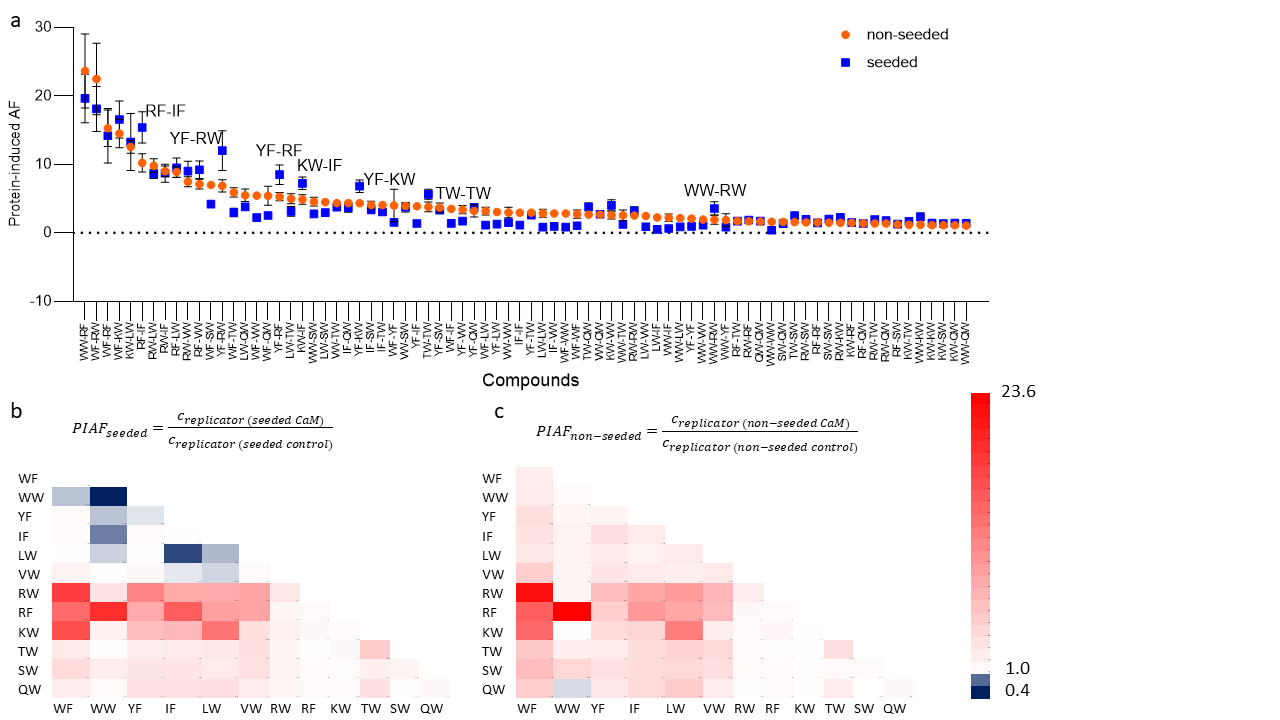


Figure S12. Effect of seeding (initial replicator population) on competitive selection. a) Protein-induced amplification factor (PIAF) profiles obtained for seeded (blue square) and non-seeded (orange circles) samples. Compounds are ordered in descending PIAF based on the non-seeded condition. b) Heatmap representation of PIAF values for the seeded sample. c) corresponding heatmap for the non-seeded sample.


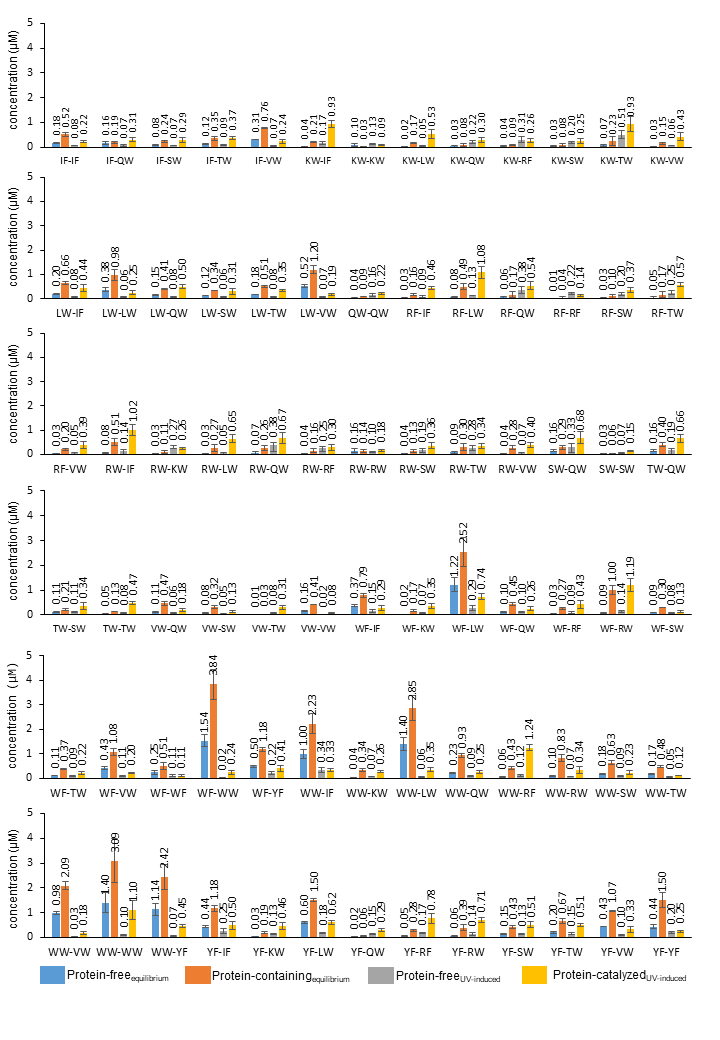


Figure S13. Comparison of product distributions in the thermodynamically controlled and light-driven systems. Concentrations of replicators at equilibrium in the thermodynamically controlled system for protein-free (blue) and protein-containing (orange) samples. Concentrations of replicators at steady state in the light-driven system for protein-free (gray) and protein-catalyzed (yellow) samples.


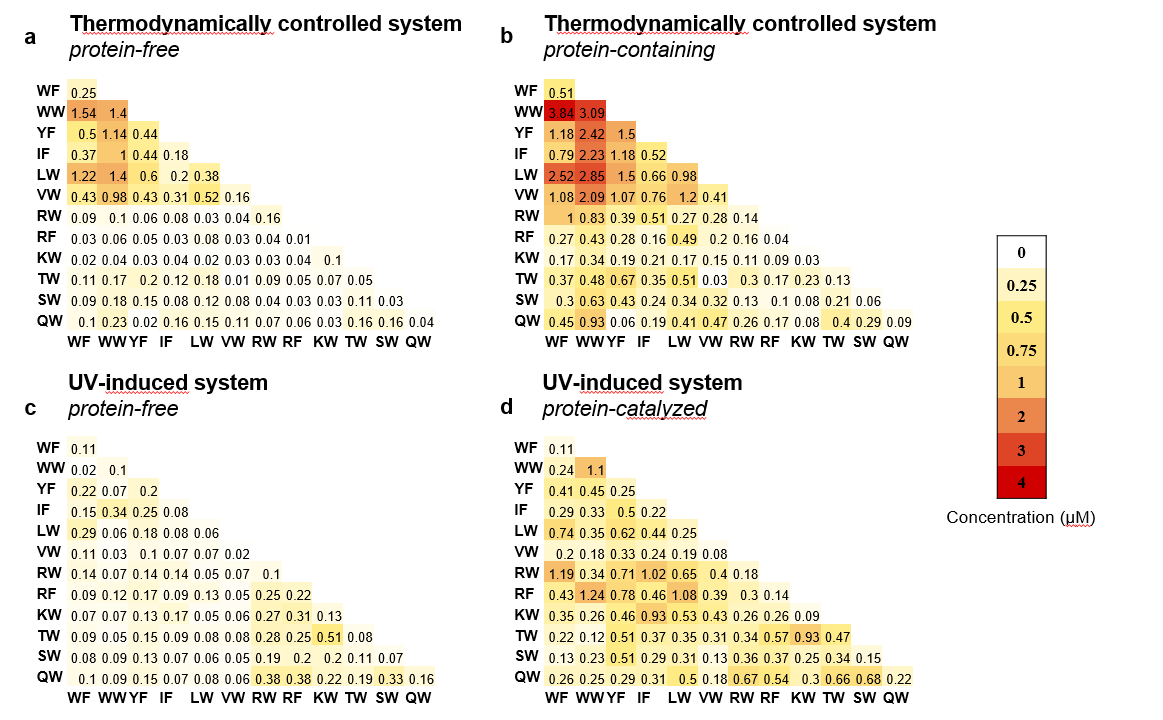


Figure S14. Comparison of product distribution shown in heatmap representation. Concentration of replicators at equilibrium in the thermodynamically controlled system for protein free (a) and protein-containing system (b). Concentration of the replicators at steady-state in the light-driven system for protein-free (c) and protein-catalyzed system (d).

# Supplementary Tables

## Table S1. Identification of the oxidized Met residues in the CaM sequence.

| **m/z** | **m/z** | **Type of modification** | **Start** | **End** | **Sequence** | **Relative %** |
| --- | --- | --- | --- | --- | --- | --- |
| **monoisotopic** | **average** |  |  |  |  |  |
| 805.4236 | 805.9773 |  | 41 | 47 | (K)ELGTVMR(S) | 98.1 |
| **821.4186** | **821.9766** | 1Oxidation | 41 | 47 | (K)ELGTVMR(S) | 1.9 |
| 907.4367 | 907.9584 |  | 32 | 40 | (K)DGDGTITTK(E) |  |
| 956.4724 | 957.0774 |  | 24 | 31 | (K)EAFSLFDK(D) |  |
| 1011.526 | 1012.16 |  | 97 | 104 | (R)EAFRVFDK(D) |  |
| 1028.519 | 1029.21 |  | 117 | 125 | (R)HVMTNLGEK(L) | 95.2 |
| **1044.514** | **1045.21** | 1Oxidation | 117 | 125 | (R)HVMTNLGEK(L) | 4.8 |
| 1093.464 | 1094.083 |  | 88 | 96 | (K)DTDSEEEIR(E) |  |
| 1265.612 | 1266.361 |  | 105 | 116 | (K)DGNGYISAAELR(H) |  |
| 1349.625 | 1350.496 |  | 126 | 136 | (K)LTDEEVDEMIR(E) | 98.6 |
| 1352.6 | 1353.456 |  | 86 | 96 | (K)MKDTDSEEEIR(E) | 97.9 |
| **1365.62** | **1366.496** | 1Oxidation | 126 | 136 | (K)LTDEEVDEMIR(E) | 1.4 |
| **1368.595** | **1369.456** | 1Oxidation | 86 | 96 | (K)MKDTDSEEEIR(E) | 2.1 |
| 1596.714 | 1597.645 |  | 88 | 100 | (K)DTDSEEEIREAFR(V) |  |
| 1693.843 | 1694.913 |  | 32 | 47 | (K)DGDGTITTKELGTVMR(S) |  |
| **1709.837** | **1710.912** | 1Oxidation | 32 | 47 | (K)DGDGTITTKELGTVMR(S) | NF |
| 1754.871 | 1755.937 |  | 101 | 116 | (R)VFDKDGNGYISAAELR(H) |  |
| 1844.891 | 1846.013 |  | 24 | 40 | (K)EAFSLFDKDGDGTITTK(E) |  |
| 1940.891 | 1942.122 |  | 7 | 23 | (R)SNSMADQLTEEQIAEFK(E) | 98.6 |
| **1956.886** | **1958.121** | 1Oxidation | 7 | 23 | (R)SNSMADQLTEEQIAEFK(E) | 1.4 |
| 2275.114 | 2276.549 |  | 105 | 125 | (K)DGNGYISAAELRHVMTNLGEK(L) |  |
| **2291.109** | **2292.548** | 1Oxidation | 105 | 125 | (K)DGNGYISAAELRHVMTNLGEK(L) | NF |
| 2359.127 | 2360.684 |  | 117 | 136 | (R)HVMTNLGEKLTDEEVDEMIR(E) | 96 |
| **2375.122** | **2376.683** | 1Oxidation | 117 | 136 | (R)HVMTNLGEKLTDEEVDEMIR(E) | 4.0 |
| **2391.117** | **2392.683** | 2Oxidation | 117 | 136 | (R)HVMTNLGEKLTDEEVDEMIR(E) | NF |
| 2490.08 | 2491.728 |  | 137 | 158 | (R)EADIDGDGQVNYEEFVQMMTAK(-) |  |
| **2506.075** | **2507.728** | 1Oxidation | 137 | 158 | (R)EADIDGDGQVNYEEFVQMMTAK(-) | NF |
| **2522.07** | **2523.727** | 2Oxidation | 137 | 158 | (R)EADIDGDGQVNYEEFVQMMTAK(-) | NF |
| 2580.182 | 2581.86 |  | 1 | 23 | (-)GSHMARSNSMADQLTEEQIAEFK(E) |  |
| **2596.177** | **2597.86** | 1Oxidation | 1 | 23 | (-)GSHMARSNSMADQLTEEQIAEFK(E) | NF |
| **2612.172** | **2613.859** | 2Oxidation | 1 | 23 | (-)GSHMARSNSMADQLTEEQIAEFK(E) | NF |
| 2622.192 | 2623.898 | 1Acetyl | 1 | 23 | (-)GSHMARSNSMADQLTEEQIAEFK(E) | NF |
| **2638.187** | **2639.897** | 1Acetyl 1Oxidation | 1 | 23 | (-)GSHMARSNSMADQLTEEQIAEFK(E) | NF |
| **2654.182** | **2655.897** | 1Acetyl 2Oxidation | 1 | 23 | (-)GSHMARSNSMADQLTEEQIAEFK(E) | NF |
| 2878.345 | 2880.176 |  | 7 | 31 | (R)SNSMADQLTEEQIAEFKEAFSLFDK(D) |  |
| **2894.34** | **2896.176** | 1Oxidation | 7 | 31 | (R)SNSMADQLTEEQIAEFKEAFSLFDK(D) | NF |
| 3820.687 | 3823.202 |  | 126 | 158 | (K)LTDEEVDEMIREADIDGDGQVNYEEFVQMMTAK(-) |  |
| **3836.682** | **3839.201** | 1Oxidation | 126 | 158 | (K)LTDEEVDEMIREADIDGDGQVNYEEFVQMMTAK(-) | NF |
| **3852.677** | **3855.201** | 2Oxidation | 126 | 158 | (K)LTDEEVDEMIREADIDGDGQVNYEEFVQMMTAK(-) | NF |
| **3868.672** | **3871.2** | 3Oxidation | 126 | 158 | (K)LTDEEVDEMIREADIDGDGQVNYEEFVQMMTAK(-) | NF |

Product ions were searched based on their expected average m/z and relative ratio of the oxidized and intact protein was estimated with the area under the curve (AUC) of the appropriate fragment using the following formula: Relative % = AUC_modified_/(AUC_modified_+AUC_intact_) x 100. Product ions labelled with NF (not found) were not detectable during the measurement.

## Table S2. Light intensity-dependent amplifications (LIAF) obtained upon increasing power density from 2.55 to 5.10 mW cm^-2^ for control and CaM containing sample

| **Compounds** | LIAF_i_ (control) | SD _CAi, control_ | LIAF_i_ (CaM) | SD _CAi, CaM_ |
| --- | --- | --- | --- | --- |
| **IF-IF** | 51.92 | 10.46 | 153.15 | 32.71 |
| **IF-QW** | 24.82 | 7.73 | 100.52 | 18.77 |
| **IF-SW** | 49.73 | 0.23 | 58.68 | 10.37 |
| **IF-TW** | 48.69 | 1.81 | 211.31 | 7.84 |
| **IF-VW** | 4.15 | 3.17 | 50.35 | 27.05 |
| **KW-IF** | 32.06 | 5.35 | 55.15 | 19.94 |
| **KW-KW** | 34.33 | 0.41 | 350.54 | 11.67 |
| **KW-LW** | 42.93 | 0.46 | 385.90 | 10.30 |
| **KW-QW** | 47.55 | 7.51 | 193.51 | 9.58 |
| **KW-RF** | 5.21 | 13.09 | 15.30 | 4.19 |
| **KW-SW** | 15.87 | 11.64 | 8.97 | 5.17 |
| **KW-TW** | 15.79 | 0.52 | 197.88 | 53.35 |
| **KW-VW** | 16.08 | 6.63 | 6.26 | 2.71 |
| **LW-IF** | 107.98 | 22.82 | 157.65 | 21.53 |
| **LW-LW** | 2.34 | 2.19 | 11.39 | 14.39 |
| **LW-QW** | 31.80 | 1.57 | 82.36 | 3.70 |
| **LW-SW** | 30.12 | 4.90 | 67.46 | 29.09 |
| **LW-TW** | 24.56 | 10.08 | 58.28 | 21.28 |
| **LW-VW** | 13.29 | 11.20 | 7.29 | 1.74 |
| **QW-QW** | 23.86 | 2.95 | 67.99 | 15.79 |
| **RF-IF** | 30.59 | 1.23 | 167.34 | 7.39 |
| **RF-LW** | 38.39 | 0.17 | 340.90 | 6.14 |
| **RF-QW** | 25.60 | 2.04 | 250.34 | 62.63 |
| **RF-RF** | 27.53 | 7.02 | 26.25 | 8.91 |
| **RF-SW** | 1.53 | 6.92 | 2.66 | 5.75 |
| **RF-TW** | 24.04 | 5.70 | 13.10 | 3.54 |
| **RF-VW** | 24.64 | 7.62 | 7.84 | 3.22 |
| **RW-IF** | 37.60 | 6.60 | 30.37 | 35.84 |
| **RW-KW** | 96.19 | 25.51 | 129.86 | 23.81 |
| **RW-LW** | 17.91 | 8.23 | 30.27 | 6.60 |
| **RW-QW** | 27.95 | 7.32 | 70.72 | 18.61 |
| **RW-RF** | 11.64 | 12.37 | 8.50 | 5.09 |
| **RW-RW** | 12.25 | 9.44 | 8.33 | 4.79 |
| **RW-SW** | 13.79 | 12.51 | 27.22 | 5.67 |
| **RW-TW** | 25.50 | 5.02 | 68.08 | 22.20 |
| **RW-VW** | 8.26 | 3.22 | 6.30 | 1.47 |
| **SW-QW** | 12.91 | 5.03 | 48.92 | 18.68 |
| **SW-SW** | 25.10 | 12.69 | 66.51 | 49.19 |
| **TW-QW** | 18.45 | 16.82 | 20.48 | 4.80 |
| **TW-SW** | 23.95 | 1.22 | 25.59 | 10.58 |
| **TW-TW** | 8.16 | 5.72 | 9.95 | 8.55 |
| **VW-QW** | 35.73 | 8.07 | 57.17 | 30.26 |
| **VW-SW** | 21.59 | 1.29 | 84.65 | 9.38 |
| **VW-TW** | 53.94 | 2.01 | 234.08 | 62.15 |
| **VW-VW** | 10.87 | 2.69 | 32.29 | 7.39 |
| **WF-IF** | 128.62 | 8.66 | 451.46 | 32.16 |
| **WF-KW** | 16.62 | 5.36 | 240.89 | 86.03 |
| **WF-LW** | 98.16 | 18.54 | 311.18 | 71.99 |
| **WF-QW** | 47.33 | 5.91 | 37.71 | 34.38 |
| **WF-RF** | 2.55 | 0.62 | 38.88 | 15.13 |
| **WF-RW** | 21.13 | 3.81 | 474.57 | 111.56 |
| **WF-SW** | 41.43 | 7.96 | 289.62 | 52.99 |
| **WF-TW** | 47.38 | 7.17 | 280.00 | 9.41 |
| **WF-VW** | 72.47 | 1.55 | 393.53 | 9.42 |
| **WF-WF** | 99.81 | 25.45 | 273.47 | 64.28 |
| **WF-WW** | 7.38 | 2.84 | 20.80 | 7.60 |
| **WF-YF** | 130.24 | 12.43 | 519.67 | 94.07 |
| **WW-IF** | 115.79 | 15.13 | 253.58 | 51.78 |
| **WW-KW** | 19.87 | 5.14 | 23.25 | 8.34 |
| **WW-LW** | 19.10 | 6.61 | 40.87 | 12.03 |
| **WW-QW** | 10.57 | 5.04 | 9.51 | 1.71 |
| **WW-RF** | 28.49 | 8.10 | 300.67 | 81.07 |
| **WW-RW** | 20.16 | 7.04 | 38.51 | 20.38 |
| **WW-SW** | 19.99 | 1.70 | 91.16 | 28.26 |
| **WW-TW** | 15.41 | 4.48 | 39.13 | 11.19 |
| **WW-VW** | 10.43 | 1.90 | 20.35 | 3.97 |
| **WW-WW** | 30.62 | 3.10 | 49.29 | 4.93 |
| **WW-YF** | 16.63 | 1.03 | 31.26 | 9.61 |
| **YF-IF** | 102.20 | 6.64 | 396.52 | 28.61 |
| **YF-KW** | 52.89 | 14.35 | 101.10 | 39.91 |
| **YF-LW** | 78.96 | 5.39 | 239.25 | 16.24 |
| **YF-QW** | 43.84 | 11.42 | 139.85 | 23.26 |
| **YF-RF** | 58.68 | 9.94 | 309.83 | 63.33 |
| **YF-RW** | 52.46 | 4.14 | 359.87 | 62.90 |
| **YF-SW** | 15.85 | 11.01 | 58.31 | 38.23 |
| **YF-TW** | 49.15 | 11.20 | 144.01 | 54.23 |
| **YF-VW** | 48.95 | 3.52 | 165.46 | 11.82 |
| **YF-YF** | 48.04 | 10.74 | 21.64 | 8.81 |

## Table S3. Fitted rate constants for representative dimers without the protein template.

| **Compounds** | **s_p1_**  10^-3^ mM^-1^ (cm^2^) W^-1^ s^-1^ | | **s_p2_**  x mM^-1^ (cm^2^)^2^ W^-2^ s^-1^ | | **s_a1_**  x 10^3^ mM^-2^ (cm^2^) W^-1^ s^-1^ | | **s_a2_**  x 10^4^ mM^-2^ (cm^2^)^2^ W^-2^ s^-1^ | | **b**  x 10^-2^ (cm^2^)^0.5^ W^-0.5^ s^-1^ | |
| --- | --- | --- | --- | --- | --- | --- | --- | --- | --- | --- |
|  | mean | SEM | mean | SEM | mean | SEM | mean | SEM | mean | SEM |
| **IF-IF** | 0.083 | 0.012 | 0.291 | 0.003 | 1.414 | 0.008 | 1.511 | 0.089 | 4.232 | 0.021 |
| **IF-QW** | 0.077 | 0.007 | 0.244 | 0.002 | 1.012 | 0.006 | 0.202 | 0.037 | 2.910 | 0.015 |
| **IF-SW** | 0.087 | 0.007 | 0.252 | 0.002 | 1.257 | 0.007 | 0.345 | 0.078 | 3.627 | 0.019 |
| **IF-TW** | 0.058 | 0.011 | 0.294 | 0.002 | 1.262 | 0.007 | 0.431 | 0.066 | 3.628 | 0.020 |
| **IF-VW** | 0.136 | 0.010 | 0.466 | 0.003 | 1.458 | 0.008 | 1.920 | 0.112 | 4.494 | 0.024 |
| **KW-IF** | 0.255 | 0.035 | 0.850 | 0.008 | 1.117 | 0.022 | 2.895 | 0.183 | 3.566 | 0.061 |
| **KW-KW** | 0.314 | 0.054 | 1.427 | 0.009 | 1.382 | 0.019 | 2.470 | 0.167 | 4.358 | 0.048 |
| **KW-LW** | 0.760 | 0.008 | 0.084 | 0.005 | 1.318 | 0.009 | 5.281 | 0.179 | 4.547 | 0.022 |
| **KW-QW** | 1.032 | 0.127 | 2.182 | 0.017 | 0.923 | 0.014 | 0.846 | 0.221 | 2.783 | 0.035 |
| **KW-RF** | 1.801 | 0.100 | 1.730 | 0.019 | 0.863 | 0.016 | 1.573 | 0.135 | 2.632 | 0.043 |
| **KW-SW** | 1.365 | 0.071 | 1.911 | 0.013 | 1.121 | 0.013 | 1.086 | 0.120 | 3.388 | 0.032 |
| **KW-TW** | 3.517 | 0.113 | 2.681 | 0.024 | 0.826 | 0.011 | 8.000 | 0.102 | 3.271 | 0.022 |
| **KW-VW** | 0.299 | 0.017 | 0.375 | 0.004 | 0.383 | 0.002 | 0.070 | 0.051 | 1.190 | 0.005 |
| **LW-IF** | 0.381 | 0.016 | 0.547 | 0.005 | 1.286 | 0.007 | 0.319 | 0.088 | 3.784 | 0.018 |
| **LW-LW** | 0.465 | 0.029 | 0.788 | 0.008 | 1.186 | 0.011 | 7.859 | 0.216 | 4.692 | 0.027 |
| **LW-QW** | 0.105 | 0.013 | 0.453 | 0.003 | 1.336 | 0.010 | 0.866 | 0.098 | 3.954 | 0.027 |
| **LW-SW** | 0.180 | 0.008 | 0.410 | 0.003 | 1.350 | 0.006 | 1.884 | 0.046 | 4.192 | 0.020 |
| **LW-TW** | 0.158 | 0.009 | 0.473 | 0.003 | 1.548 | 0.012 | 3.454 | 0.121 | 4.935 | 0.038 |
| **LW-VW** | 0.183 | 0.012 | 0.689 | 0.004 | 1.345 | 0.006 | 6.919 | 0.102 | 4.941 | 0.019 |
| ***QW-QW**** | *NF* | *NF* | *NF* | *NF* | *NF* | *NF* | *NF* | *NF* | *NF* | *NF* |
| ***RF-IF**** | *NF* | *NF* | *NF* | *NF* | *NF* | *NF* | *NF* | *NF* | *NF* | *NF* |
| **RF-LW** | 3.189 | 0.089 | 0.040 | 0.006 | 0.478 | 0.005 | 0.388 | 0.034 | 1.453 | 0.015 |
| **RF-QW** | 0.288 | 0.070 | 2.095 | 0.011 | 0.659 | 0.004 | 1.821 | 0.051 | 2.064 | 0.007 |
| **RF-RF** | 1.126 | 0.060 | 2.857 | 0.101 | 0.467 | 0.013 | 1.026 | 0.145 | 1.628 | 0.042 |
| **RF-SW** | 0.773 | 0.066 | 1.597 | 0.012 | 0.787 | 0.017 | 1.575 | 0.154 | 2.499 | 0.043 |
| **RF-TW** | 0.619 | 0.083 | 1.998 | 0.020 | 0.681 | 0.022 | 3.024 | 0.217 | 2.391 | 0.055 |
| **RF-VW** | 0.307 | 0.003 | 0.031 | 0.001 | 1.215 | 0.005 | 0.046 | 0.090 | 3.413 | 0.013 |
| **RW-IF** | 0.628 | 0.011 | 0.383 | 0.003 | 0.839 | 0.003 | 0.012 | 0.007 | 2.368 | 0.010 |
| **RW-KW** | 0.625 | 0.057 | 1.441 | 0.012 | 1.325 | 0.027 | 1.166 | 0.113 | 3.827 | 0.078 |
| **RW-LW** | 0.449 | 0.007 | 0.291 | 0.003 | 1.253 | 0.003 | 1.344 | 0.051 | 3.838 | 0.010 |
| **RW-QW** | 1.597 | 0.063 | 2.204 | 0.012 | 0.545 | 0.004 | 0.815 | 0.047 | 1.635 | 0.007 |
| **RW-RF** | 4.134 | 0.054 | 0.194 | 0.021 | 1.440 | 0.020 | 3.600 | 0.215 | 4.468 | 0.050 |
| **RW-RW** | 0.189 | 0.028 | 0.698 | 0.005 | 0.912 | 0.022 | 17.356 | 0.195 | 5.134 | 0.055 |
| **RW-SW** | 0.968 | 0.060 | 1.396 | 0.012 | 1.244 | 0.020 | 0.922 | 0.121 | 3.668 | 0.052 |
| **RW-TW** | 1.646 | 0.081 | 1.988 | 0.022 | 0.811 | 0.022 | 0.939 | 0.091 | 2.432 | 0.058 |
| **RW-VW** | 1.065 | 0.007 | 0.009 | 0.001 | 1.119 | 0.006 | 0.179 | 0.043 | 3.197 | 0.016 |
| **SW-QW** | 0.254 | 0.075 | 2.298 | 0.011 | 0.824 | 0.004 | 0.783 | 0.055 | 2.406 | 0.007 |
| **SW-SW** | 0.261 | 0.021 | 0.823 | 0.005 | 1.030 | 0.004 | 2.959 | 0.049 | 3.509 | 0.015 |
| **TW-QW** | 2.634 | 0.081 | 1.560 | 0.018 | 0.468 | 0.007 | 0.834 | 0.099 | 1.566 | 0.020 |
| **TW-SW** | 0.849 | 0.025 | 0.827 | 0.006 | 0.475 | 0.002 | 0.076 | 0.052 | 1.464 | 0.006 |
| **TW-TW** | 0.174 | 0.014 | 0.513 | 0.004 | 1.142 | 0.005 | 0.177 | 0.078 | 3.326 | 0.016 |
| **VW-QW** | 0.161 | 0.007 | 0.358 | 0.002 | 1.439 | 0.003 | 2.679 | 0.040 | 4.543 | 0.011 |
| **VW-SW** | 0.127 | 0.005 | 0.290 | 0.002 | 1.441 | 0.003 | 2.725 | 0.031 | 4.568 | 0.016 |
| **VW-TW** | 0.123 | 0.008 | 0.301 | 0.003 | 1.372 | 0.009 | 0.316 | 0.080 | 3.953 | 0.025 |
| **VW-VW** | 0.120 | 0.005 | 0.294 | 0.003 | 1.524 | 0.003 | 6.623 | 0.052 | 5.639 | 0.003 |
| **WF-IF** | 0.396 | 0.043 | 1.165 | 0.008 | 1.561 | 0.007 | 3.112 | 0.045 | 4.869 | 0.017 |
| **WF-KW** | 2.931 | 0.015 | 0.069 | 0.007 | 1.135 | 0.004 | 4.504 | 0.070 | 3.980 | 0.012 |
| **WF-LW** | 2.737 | 0.060 | 2.073 | 0.017 | 2.073 | 0.012 | 1.324 | 0.160 | 5.870 | 0.015 |
| **WF-QW** | 0.223 | 0.026 | 0.796 | 0.006 | 1.370 | 0.019 | 4.755 | 0.122 | 4.624 | 0.047 |
| ***WF-RF**** | *NF* | *NF* | *NF* | *NF* | *NF* | *NF* | *NF* | *NF* | *NF* | *NF* |
| **WF-RW** | 5.481 | 0.016 | 0.038 | 0.007 | 0.904 | 0.005 | 5.205 | 0.063 | 3.358 | 0.007 |
| **WF-SW** | 0.200 | 0.021 | 0.674 | 0.004 | 1.847 | 0.017 | 2.556 | 0.155 | 5.674 | 0.044 |
| **WF-TW** | 0.251 | 0.022 | 0.819 | 0.006 | 1.580 | 0.015 | 4.566 | 0.115 | 5.206 | 0.042 |
| **WF-VW** | 0.132 | 0.031 | 1.060 | 0.006 | 1.229 | 0.011 | 10.146 | 0.078 | 5.003 | 0.020 |
| **WF-WF** | 0.760 | 0.064 | 1.684 | 0.014 | 2.049 | 0.029 | 8.483 | 0.219 | 7.137 | 0.075 |
| **WF-WW** | 0.108 | 0.007 | 0.405 | 0.002 | 1.143 | 0.008 | 11.599 | 0.154 | 5.214 | 0.002 |
| **WF-YF** | 0.708 | 0.048 | 1.010 | 0.010 | 2.530 | 0.012 | 4.788 | 0.118 | 7.642 | 0.026 |
| **WW-IF** | 0.356 | 0.063 | 2.406 | 0.013 | 2.142 | 0.006 | 1.755 | 0.069 | 6.009 | 0.010 |
| **WW-KW** | 0.299 | 0.021 | 1.562 | 0.008 | 1.062 | 0.007 | 4.179 | 0.100 | 3.870 | 0.018 |
| **WW-LW** | 0.718 | 0.006 | 0.057 | 0.006 | 1.397 | 0.007 | 0.605 | 0.072 | 4.057 | 0.019 |
| **WW-QW** | 0.789 | 0.048 | 1.642 | 0.010 | 1.230 | 0.012 | 3.147 | 0.170 | 4.148 | 0.031 |
| **WW-RF** | 0.218 | 0.019 | 0.872 | 0.003 | 0.836 | 0.004 | 2.633 | 0.074 | 2.801 | 0.006 |
| **WW-RW** | 4.390 | 0.048 | 0.478 | 0.017 | 0.921 | 0.011 | 14.952 | 0.214 | 4.984 | 0.019 |
| **WW-SW** | 2.937 | 0.027 | 0.557 | 0.010 | 0.478 | 0.013 | 29.546 | 0.242 | 5.737 | 0.024 |
| **WW-TW** | 1.559 | 0.024 | 0.855 | 0.009 | 0.240 | 0.022 | 33.720 | 0.249 | 5.805 | 0.096 |
| **WW-VW** | 0.110 | 0.006 | 0.476 | 0.003 | 0.557 | 0.005 | 24.279 | 0.111 | 5.301 | 0.008 |
| **WW-WW** | 1.540 | 0.099 | 5.424 | 0.038 | 1.177 | 0.010 | 3.207 | 0.225 | 4.246 | 0.027 |
| **WW-YF** | 0.301 | 0.021 | 0.993 | 0.005 | 1.496 | 0.013 | 6.400 | 0.081 | 5.342 | 0.036 |
| **YF-IF** | 0.061 | 0.038 | 0.747 | 0.003 | 0.621 | 0.003 | 6.195 | 0.055 | 2.572 | 0.006 |
| **YF-KW** | 0.268 | 0.029 | 0.775 | 0.006 | 0.992 | 0.008 | 0.485 | 0.115 | 2.916 | 0.021 |
| **YF-LW** | 0.236 | 0.030 | 0.969 | 0.005 | 1.081 | 0.013 | 14.872 | 0.110 | 5.144 | 0.024 |
| **YF-QW** | 4.242 | 0.026 | 0.033 | 0.006 | 0.392 | 0.005 | 1.966 | 0.033 | 1.442 | 0.013 |
| **YF-RF** | 0.067 | 0.017 | 0.496 | 0.002 | 0.815 | 0.004 | 1.341 | 0.061 | 2.453 | 0.006 |
| **YF-RW** | 0.097 | 0.020 | 0.515 | 0.002 | 1.018 | 0.003 | 0.949 | 0.059 | 3.000 | 0.006 |
| **YF-SW** | 0.183 | 0.028 | 0.789 | 0.009 | 0.399 | 0.003 | 0.287 | 0.052 | 1.242 | 0.009 |
| **YF-TW** | 2.472 | 0.037 | 0.151 | 0.015 | 0.968 | 0.011 | 0.985 | 0.183 | 2.875 | 0.030 |
| **YF-VW** | 0.121 | 0.013 | 0.603 | 0.002 | 1.350 | 0.003 | 5.283 | 0.048 | 4.621 | 0.009 |
| **YF-YF** | 0.257 | 0.032 | 0.636 | 0.007 | 1.115 | 0.023 | 1.971 | 0.108 | 3.368 | 0.061 |

s_p1_: rate constant of spontaneous synthesis by proximity-controlled radical substitution

s_p2_: rate constant of spontaneous synthesis by proximity-controlled concerted metathesis

s_a1_: rate constant of autocatalytic synthesis by proximity-controlled radical substitution

s_a2_: rate constant of autocatalytic synthesis by proximity-controlled concerted metathesis

b: rate constant of dimers break down via diffusion-controlled radical substitution

Dimers marked with asterisk (*) were skipped from the evaluation, due to the uncertainty of the experimental data.

NF: not fitted

## Table S4. Fitted rate constants for representative dimers with the protein template.

| **Compounds** | **s_p1_**  10^-3^ mM^-1^ (cm^2^) W^-1^ s^-1^ | | **s_p2_**  x mM^-1^ (cm^2^)^2^ W^-2^ s^-1^ | | **s_a1_**  x 10^3^ mM^-2^ (cm^2^) W^-1^ s^-1^ | | **s_a2_**  x 10^4^ mM^-2^ (cm^2^)^2^ W^-2^ s^-1^ | | **b**  x 10^-2^ (cm^2^)^0.5^ W^-0.5^ s^-1^ | |
| --- | --- | --- | --- | --- | --- | --- | --- | --- | --- | --- |
|  | mean | SEM | mean | SEM | mean | SEM | mean | SEM | mean | SEM |
| **IF-IF** | 0.382 | 0.077 | 2.458 | 0.026 | 1.341 | 0.052 | 26.782 | 0.353 | 7.584 | 0.116 |
| **IF-QW** | 1.056 | 0.412 | 5.104 | 0.069 | 2.121 | 0.041 | 16.068 | 0.637 | 8.172 | 0.099 |
| **IF-SW** | 1.006 | 0.113 | 4.267 | 0.045 | 2.317 | 0.034 | 18.102 | 0.435 | 8.958 | 0.082 |
| **IF-TW** | 1.021 | 0.171 | 4.267 | 0.055 | 2.242 | 0.048 | 21.195 | 0.331 | 8.982 | 0.111 |
| **IF-VW** | 0.453 | 0.072 | 3.534 | 0.045 | 1.437 | 0.041 | 23.887 | 0.271 | 7.462 | 0.086 |
| **KW-IF** | 0.922 | 0.109 | 5.157 | 0.081 | 0.633 | 0.060 | 23.104 | 0.245 | 4.439 | 0.101 |
| **KW-KW** | 5.084 | 0.073 | 2.148 | 0.023 | 0.473 | 0.003 | 1.490 | 0.114 | 2.035 | 0.011 |
| **KW-LW** | 2.703 | 0.290 | 4.478 | 0.206 | 3.383 | 0.080 | 13.001 | 0.917 | 10.487 | 0.192 |
| **KW-QW** | 4.331 | 0.268 | 6.018 | 0.130 | 2.006 | 0.029 | 4.034 | 0.738 | 6.218 | 0.077 |
| **KW-RF** | 0.843 | 0.094 | 4.458 | 0.025 | 0.718 | 0.006 | 0.375 | 0.075 | 2.230 | 0.016 |
| **KW-SW** | 8.607 | 0.193 | 5.033 | 0.051 | 1.836 | 0.021 | 4.500 | 0.293 | 5.947 | 0.055 |
| **KW-TW** | 49.146 | 0.298 | 1.037 | 0.112 | 0.264 | 0.014 | 27.696 | 0.098 | 4.064 | 0.018 |
| **KW-VW** | 3.367 | 0.164 | 4.079 | 0.058 | 3.798 | 0.031 | 3.812 | 0.336 | 10.544 | 0.076 |
| **LW-IF** | 1.328 | 0.140 | 5.155 | 0.058 | 2.513 | 0.043 | 20.912 | 0.305 | 9.556 | 0.096 |
| **LW-LW** | 0.776 | 0.138 | 5.447 | 0.049 | 2.449 | 0.041 | 20.034 | 0.424 | 9.754 | 0.111 |
| **LW-QW** | 3.025 | 0.279 | 5.075 | 0.148 | 4.000 | 0.057 | 9.234 | 0.715 | 11.676 | 0.139 |
| **LW-SW** | 1.442 | 0.253 | 7.947 | 0.146 | 2.946 | 0.043 | 11.566 | 0.709 | 9.878 | 0.111 |
| **LW-TW** | 2.286 | 0.212 | 4.879 | 0.097 | 4.478 | 0.080 | 6.389 | 0.832 | 12.978 | 0.204 |
| **LW-VW** | 0.881 | 0.263 | 5.740 | 0.065 | 2.261 | 0.048 | 21.744 | 0.575 | 9.657 | 0.103 |
| ***QW-QW**** | *NF* | *NF* | *NF* | *NF* | *NF* | *NF* | *NF* | *NF* | *NF* | *NF* |
| ***RF-IF**** | *NF* | *NF* | *NF* | *NF* | *NF* | *NF* | *NF* | *NF* | *NF* | *NF* |
| **RF-LW** | 12.005 | 0.485 | 2.452 | 0.194 | 1.619 | 0.048 | 18.349 | 0.269 | 5.916 | 0.094 |
| **RF-QW** | 0.456 | 0.187 | 4.991 | 0.032 | 1.141 | 0.012 | 3.817 | 0.114 | 3.542 | 0.026 |
| **RF-RF** | 5.725 | 0.077 | 1.649 | 0.020 | 0.316 | 0.003 | 0.079 | 0.035 | 1.165 | 0.008 |
| **RF-SW** | 5.186 | 0.255 | 4.355 | 0.097 | 1.845 | 0.037 | 6.859 | 0.361 | 6.007 | 0.096 |
| **RF-TW** | 9.973 | 0.510 | 2.919 | 0.241 | 1.716 | 0.049 | 7.548 | 0.492 | 5.453 | 0.126 |
| **RF-VW** | 0.752 | 0.144 | 3.848 | 0.041 | 0.806 | 0.053 | 25.778 | 0.367 | 5.767 | 0.102 |
| **RW-IF** | 1.718 | 0.237 | 6.617 | 0.084 | 1.544 | 0.040 | 15.160 | 0.184 | 5.456 | 0.090 |
| **RW-KW** | 0.474 | 0.101 | 6.147 | 0.043 | 1.032 | 0.010 | 0.702 | 0.176 | 3.219 | 0.026 |
| **RW-LW** | 2.037 | 0.102 | 2.918 | 0.043 | 3.655 | 0.027 | 3.539 | 0.262 | 9.600 | 0.050 |
| **RW-QW** | 9.735 | 0.253 | 6.466 | 0.065 | 1.558 | 0.019 | 5.481 | 0.164 | 4.712 | 0.043 |
| **RW-RF** | 1.667 | 0.156 | 4.785 | 0.046 | 1.318 | 0.020 | 1.784 | 0.330 | 4.032 | 0.051 |
| **RW-RW** | 0.589 | 0.051 | 2.550 | 0.017 | 1.027 | 0.011 | 1.531 | 0.208 | 3.261 | 0.028 |
| **RW-SW** | 12.473 | 0.378 | 4.258 | 0.128 | 2.500 | 0.046 | 10.283 | 0.630 | 8.275 | 0.118 |
| **RW-TW** | 7.731 | 0.364 | 19.777 | 0.812 | 2.902 | 0.111 | 7.322 | 0.960 | 9.242 | 0.301 |
| **RW-VW** | 18.641 | 0.269 | 0.913 | 0.142 | 1.481 | 0.065 | 18.225 | 0.472 | 6.516 | 0.142 |
| **SW-QW** | 3.105 | 0.158 | 5.024 | 0.049 | 1.974 | 0.019 | 5.206 | 0.177 | 5.634 | 0.042 |
| **SW-SW** | 0.622 | 0.074 | 3.508 | 0.036 | 1.735 | 0.019 | 14.557 | 0.321 | 7.197 | 0.047 |
| **TW-QW** | 15.189 | 0.337 | 4.046 | 0.228 | 2.377 | 0.028 | 8.493 | 0.387 | 7.054 | 0.065 |
| **TW-SW** | 1.548 | 0.314 | 6.448 | 0.154 | 2.283 | 0.051 | 8.522 | 0.805 | 7.483 | 0.125 |
| **TW-TW** | 0.324 | 0.018 | 1.703 | 0.072 | 0.550 | 0.031 | 6.416 | 0.399 | 2.347 | 0.093 |
| **VW-QW** | 1.859 | 0.095 | 3.792 | 0.055 | 1.669 | 0.040 | 14.130 | 0.412 | 6.938 | 0.110 |
| **VW-SW** | 0.544 | 0.042 | 3.578 | 0.027 | 1.612 | 0.013 | 8.054 | 0.182 | 6.034 | 0.040 |
| **VW-TW** | 1.225 | 0.429 | 4.814 | 0.065 | 2.082 | 0.033 | 14.392 | 0.470 | 7.808 | 0.076 |
| **VW-VW** | 0.425 | 0.026 | 2.252 | 0.022 | 1.638 | 0.013 | 19.496 | 0.268 | 7.799 | 0.050 |
| **WF-IF** | 0.127 | 0.014 | 6.100 | 0.025 | 2.559 | 0.014 | 2.021 | 0.132 | 7.463 | 0.037 |
| **WF-KW** | 1.489 | 0.199 | 5.751 | 0.054 | 1.427 | 0.022 | 3.173 | 0.311 | 4.470 | 0.056 |
| **WF-LW** | 2.138 | 0.229 | 5.864 | 0.049 | 4.030 | 0.022 | 0.392 | 0.144 | 10.023 | 0.052 |
| **WF-QW** | 0.519 | 0.061 | 5.659 | 0.053 | 1.252 | 0.013 | 1.755 | 0.245 | 3.982 | 0.034 |
| ***WF-RF**** | *NF* | *NF* | *NF* | *NF* | *NF* | *NF* | *NF* | *NF* | *NF* | *NF* |
| **WF-RW** | 0.520 | 0.191 | 8.249 | 0.064 | 0.625 | 0.022 | 8.695 | 0.040 | 2.486 | 0.046 |
| **WF-SW** | 0.458 | 0.060 | 4.041 | 0.017 | 0.514 | 0.004 | 0.632 | 0.046 | 1.968 | 0.011 |
| **WF-TW** | 0.420 | 0.045 | 5.497 | 0.031 | 1.003 | 0.011 | 1.200 | 0.157 | 3.261 | 0.029 |
| **WF-VW** | 0.104 | 0.008 | 4.909 | 0.014 | 1.749 | 0.008 | 1.825 | 0.060 | 5.372 | 0.024 |
| **WF-WF** | 0.729 | 0.016 | 5.132 | 0.036 | 1.424 | 0.015 | 5.344 | 0.107 | 5.450 | 0.038 |
| **WF-WW** | 4.781 | 0.313 | 3.628 | 0.098 | 3.662 | 0.144 | 88.915 | 2.126 | 22.408 | 0.319 |
| **WF-YF** | 0.382 | 0.261 | 5.894 | 0.031 | 3.349 | 0.030 | 6.868 | 0.241 | 9.951 | 0.071 |
| **WW-IF** | 0.075 | 0.011 | 8.245 | 0.023 | 0.852 | 0.004 | 0.027 | 0.022 | 2.576 | 0.011 |
| **WW-KW** | 7.359 | 0.253 | 4.075 | 0.081 | 4.696 | 0.050 | 5.552 | 0.755 | 13.567 | 0.122 |
| **WW-LW** | 11.715 | 0.211 | 3.044 | 0.089 | 3.234 | 0.066 | 58.471 | 0.651 | 16.564 | 0.135 |
| **WW-QW** | 8.274 | 0.251 | 5.137 | 0.180 | 3.654 | 0.039 | 15.194 | 0.772 | 12.158 | 0.100 |
| **WW-RF** | 0.371 | 0.135 | 6.920 | 0.065 | 0.868 | 0.023 | 10.430 | 0.066 | 3.189 | 0.051 |
| **WW-RW** | 24.787 | 1.334 | 0.739 | 0.164 | 2.456 | 0.034 | 19.237 | 0.733 | 9.234 | 0.113 |
| **WW-SW** | 0.825 | 0.104 | 5.372 | 0.044 | 1.522 | 0.016 | 6.133 | 0.230 | 5.357 | 0.046 |
| **WW-TW** | 8.098 | 0.103 | 2.291 | 0.040 | 0.595 | 0.031 | 57.059 | 0.522 | 10.194 | 0.083 |
| **WW-VW** | 1.978 | 0.137 | 3.557 | 0.035 | 1.278 | 0.101 | 91.446 | 0.902 | 16.575 | 0.168 |
| **WW-WW** | 20.493 | 0.985 | 2.863 | 1.072 | 1.075 | 0.045 | 38.053 | 1.350 | 6.562 | 0.187 |
| **WW-YF** | 4.158 | 0.249 | 4.286 | 0.115 | 3.636 | 0.072 | 21.679 | 0.801 | 12.486 | 0.170 |
| **YF-IF** | 0.089 | 0.009 | 4.544 | 0.019 | 0.197 | 0.012 | 17.546 | 0.116 | 2.953 | 0.024 |
| **YF-KW** | 4.753 | 0.217 | 5.165 | 0.055 | 3.182 | 0.028 | 1.945 | 0.319 | 8.666 | 0.064 |
| **YF-LW** | 0.563 | 0.225 | 6.598 | 0.049 | 0.721 | 0.063 | 43.502 | 0.412 | 7.577 | 0.096 |
| **YF-QW** | 24.204 | 0.292 | 1.246 | 0.120 | 0.786 | 0.026 | 10.692 | 0.243 | 3.878 | 0.065 |
| **YF-RF** | 0.693 | 0.115 | 5.060 | 0.059 | 1.253 | 0.043 | 20.392 | 0.253 | 5.682 | 0.079 |
| **YF-RW** | 0.927 | 0.204 | 5.054 | 0.055 | 1.627 | 0.038 | 19.318 | 0.258 | 6.543 | 0.076 |
| **YF-SW** | 1.536 | 0.218 | 5.886 | 0.068 | 1.950 | 0.019 | 4.588 | 0.157 | 5.801 | 0.047 |
| **YF-TW** | 5.137 | 0.270 | 4.583 | 0.078 | 3.904 | 0.053 | 5.298 | 0.508 | 10.804 | 0.146 |
| **YF-VW** | 0.177 | 0.064 | 4.900 | 0.017 | 2.122 | 0.021 | 16.481 | 0.164 | 8.147 | 0.043 |
| **YF-YF** | 2.594 | 0.103 | 1.589 | 0.039 | 0.566 | 0.017 | 2.568 | 0.217 | 2.018 | 0.054 |

s_p1_: rate constant of spontaneous synthesis by proximity-controlled radical substitution

s_p2_: rate constant of spontaneous synthesis by proximity-controlled concerted metathesis

s_a1_: rate constant of autocatalytic synthesis by proximity-controlled radical substitution

s_a2_: rate constant of autocatalytic synthesis by proximity-controlled concerted metathesis

b: rate constant of dimers break down via diffusion-controlled radical substitution

Dimers marked with asterisk (*) were skipped from the evaluation, due to the uncertainty of the experimental data.

NF: not fitted

## Table S5. Product distribution of the seeding pre-irradiated mixture

| **Compounds** | Mean _conc_ (μM) | SD _conc_ (μM) |
| --- | --- | --- |
| **IF-IF** | 0.019 | 0.011 |
| **IF-QW** | 0.049 | 0.040 |
| **IF-SW** | 0.033 | 0.032 |
| **IF-TW** | 0.032 | 0.026 |
| **IF-VW** | 0.022 | 0.015 |
| **KW-IF** | 0.117 | 0.113 |
| **KW-KW** | 0.063 | 0.058 |
| **KW-LW** | 0.057 | 0.052 |
| **KW-QW** | 0.080 | 0.065 |
| **KW-RF** | 0.155 | 0.170 |
| **KW-SW** | 0.094 | 0.084 |
| **KW-TW** | 0.278 | 0.266 |
| **KW-VW** | 0.073 | 0.068 |
| **LW-IF** | 0.021 | 0.016 |
| **LW-LW** | 0.039 | 0.027 |
| **LW-QW** | 0.049 | 0.041 |
| **LW-SW** | 0.005 | 0.002 |
| **LW-TW** | 0.042 | 0.039 |
| **LW-VW** | 0.037 | 0.037 |
| **QW-QW** | 0.047 | 0.038 |
| **RF-IF** | 0.023 | 0.020 |
| **RF-LW** | 0.075 | 0.063 |
| **RF-QW** | 0.090 | 0.084 |
| **RF-RF** | 0.077 | 0.076 |
| **RF-SW** | 0.063 | 0.058 |
| **RF-TW** | 0.082 | 0.077 |
| **RF-VW** | 0.013 | 0.014 |
| **RW-IF** | 0.078 | 0.065 |
| **RW-KW** | 0.130 | 0.121 |
| **RW-LW** | 0.047 | 0.032 |
| **RW-QW** | 0.147 | 0.139 |
| **RW-RF** | 0.094 | 0.096 |
| **RW-RW** | 0.053 | 0.054 |
| **RW-SW** | 0.089 | 0.088 |
| **RW-TW** | 0.118 | 0.025 |
| **RW-VW** | 0.024 | 0.023 |
| **SW-QW** | 0.158 | 0.129 |
| **SW-SW** | 0.028 | 0.021 |
| **TW-QW** | 0.112 | 0.103 |
| **TW-SW** | 0.045 | 0.037 |
| **TW-TW** | 0.057 | 0.055 |
| **VW-QW** | 0.026 | 0.024 |
| **VW-SW** | 0.027 | 0.025 |
| **VW-TW** | 0.041 | 0.037 |
| **VW-VW** | 0.007 | 0.005 |
| **WF-IF** | 0.048 | 0.002 |
| **WF-KW** | 0.116 | 0.079 |
| **WF-LW** | 0.079 | 0.010 |
| **WF-QW** | 0.106 | 0.089 |
| **WF-RF** | 0.022 | 0.012 |
| **WF-RW** | 0.152 | 0.024 |
| **WF-SW** | 0.112 | 0.134 |
| **WF-TW** | 0.087 | 0.082 |
| **WF-VW** | 0.059 | 0.031 |
| **WF-WF** | 0.058 | 0.007 |
| **WF-WW** | 0.019 | 0.009 |
| **WF-YF** | 0.122 | 0.100 |
| **WW-IF** | 0.104 | 0.039 |
| **WW-KW** | 0.097 | 0.044 |
| **WW-LW** | 0.016 | 0.003 |
| **WW-QW** | 0.176 | 0.176 |
| **WW-RF** | 0.133 | 0.021 |
| **WW-RW** | 0.148 | 0.148 |
| **WW-SW** | 0.100 | 0.076 |
| **WW-TW** | 0.094 | 0.062 |
| **WW-VW** | 0.022 | 0.005 |
| **WW-WW** | 0.172 | 0.163 |
| **WW-YF** | 0.066 | 0.056 |
| **YF-IF** | 0.051 | 0.051 |
| **YF-KW** | 0.116 | 0.117 |
| **YF-LW** | 0.083 | 0.075 |
| **YF-QW** | 0.071 | 0.090 |
| **YF-RF** | 0.082 | 0.082 |
| **YF-RW** | 0.093 | 0.090 |
| **YF-SW** | 0.087 | 0.082 |
| **YF-TW** | 0.102 | 0.087 |
| **YF-VW** | 0.040 | 0.036 |
| **YF-YF** | 0.065 | 0.071 |

## Table S6. Concentration amplifications (CA) for the MSSM dimers upon seeding with the pre-irradiated mixture at 40 min.

| **Compounds** | CA_i_ | SD _CAi_ |
| --- | --- | --- |
| **IF-IF** | 3.81 | 0.52 |
| **IF-QW** | 2.49 | 0.46 |
| **IF-SW** | 2.72 | 0.72 |
| **IF-TW** | 3.10 | 0.31 |
| **IF-VW** | 2.89 | 0.55 |
| **KW-IF** | 2.54 | 0.15 |
| **KW-KW** | 2.24 | 0.66 |
| **KW-LW** | 1.91 | 0.48 |
| **KW-QW** | 2.01 | 0.48 |
| **KW-RF** | 2.95 | 0.78 |
| **KW-SW** | 1.89 | 0.72 |
| **KW-TW** | 1.97 | 0.54 |
| **KW-VW** | 2.03 | 0.57 |
| **LW-IF** | 3.11 | 0.64 |
| **LW-LW** | 2.90 | 0.06 |
| **LW-QW** | 2.71 | 0.28 |
| **LW-SW** | 1.93 | 0.43 |
| **LW-TW** | 2.65 | 0.52 |
| **LW-VW** | 3.04 | 0.73 |
| **QW-QW** | 1.67 | 0.37 |
| **RF-IF** | 2.29 | 0.50 |
| **RF-LW** | 2.21 | 0.41 |
| **RF-QW** | 2.16 | 0.63 |
| **RF-RF** | 2.32 | 0.75 |
| **RF-SW** | 1.95 | 0.58 |
| **RF-TW** | 2.30 | 0.43 |
| **RF-VW** | 2.10 | 0.51 |
| **RW-IF** | 2.21 | 0.41 |
| **RW-KW** | 2.28 | 0.72 |
| **RW-LW** | 1.73 | 0.51 |
| **RW-QW** | 2.24 | 0.26 |
| **RW-RF** | 2.45 | 0.62 |
| **RW-RW** | 2.59 | 0.71 |
| **RW-SW** | 2.15 | 0.63 |
| **RW-TW** | 1.46 | 0.36 |
| **RW-VW** | 2.02 | 0.43 |
| **SW-QW** | 2.04 | 0.47 |
| **SW-SW** | 2.13 | 0.16 |
| **TW-QW** | 2.21 | 0.58 |
| **TW-SW** | 1.80 | 0.41 |
| **TW-TW** | 2.34 | 0.29 |
| **VW-QW** | 2.13 | 0.74 |
| **VW-SW** | 2.28 | 0.84 |
| **VW-TW** | 2.67 | 0.56 |
| **VW-VW** | 3.06 | 0.28 |
| **WF-IF** | 2.02 | 0.24 |
| **WF-KW** | 1.61 | 0.34 |
| **WF-LW** | 1.78 | 0.33 |
| **WF-QW** | 2.14 | 0.38 |
| **WF-RF** | 1.36 | 0.22 |
| **WF-RW** | 1.32 | 0.38 |
| **WF-SW** | 1.61 | 0.31 |
| **WF-TW** | 2.60 | 0.14 |
| **WF-VW** | 1.86 | 0.49 |
| **WF-WF** | 1.54 | 0.16 |
| **WF-WW** | 0.86 | 0.25 |
| **WF-YF** | 2.39 | 0.38 |
| **WW-IF** | 2.00 | 0.11 |
| **WW-KW** | 1.47 | 0.43 |
| **WW-LW** | 1.79 | 0.54 |
| **WW-QW** | 2.64 | 0.79 |
| **WW-RF** | 1.32 | 0.38 |
| **WW-RW** | 2.39 | 0.59 |
| **WW-SW** | 2.09 | 0.22 |
| **WW-TW** | 1.98 | 0.24 |
| **WW-VW** | 1.93 | 0.22 |
| **WW-WW** | 1.07 | 0.23 |
| **WW-YF** | 2.25 | 0.22 |
| **YF-IF** | 3.84 | 0.73 |
| **YF-KW** | 2.17 | 0.52 |
| **YF-LW** | 3.10 | 0.44 |
| **YF-QW** | 2.42 | 0.57 |
| **YF-RF** | 2.17 | 0.62 |
| **YF-RW** | 2.24 | 0.56 |
| **YF-SW** | 2.39 | 0.70 |
| **YF-TW** | 2.99 | 0.50 |
| **YF-VW** | 3.22 | 0.58 |
| **YF-YF** | 3.80 | 0.58 |

Table S7. Binding site analysis of the photo-foldamers on CaM. Binding of photo-foldamers to CaM was determined by the tryptic digestion and the subsequent LC-MS/MS analysis of the protein with and without the competitor **WF-S-RW**.

| **Compounds** | **Labeled segment** | **MS Fragger Localization** |
| --- | --- | --- |
| **ph-IF** | S^39^LGQNPTEAELQDMINEVDADGNGTIDFPEFLTMMAR^75^ | S^39^LGQNPTEAELQDMINEVDADGNGT^63^ |
|  | E^15^AFSLFDKDGDGTITTK^31^ | E^15^A^16^ |
| **ph-IF**  (with competitor) | E^15^AFSLFDKDGDGTITTK^31^ | E^15^A^16^ |
| **ph-KW** | S^39^LGQNPTEAELQDMINEVDADGNGTIDFPEFLTMMAR^75^ | P^44^TEAELQDMINEVDADGNGTI^64^ |
|  | E^15^AFSLFDKDGDGTITTK^31^ | E^15^A^16^ |
| **ph-KW**  (with competitor) | E^128^ADIDGDGQVNYEEFVQMMTAK^149^ | E^128^ADIDGDGQ^136^ |
| **ph-LW** | S^39^LGQNPTEAELQDMINEVDADGNGTIDFPEFLTMMAR^75^ | N^54^EVDADGNGT^63^ |
|  | E^15^AFSLFDKDGDGTITTK^31^ | E^15^A^16^ |
| **ph-LW**  (with competitor) | S^39^LGQNPTEAELQDMINEVDADGNGTIDFPEFLTMMAR^75^ | S^39^LGQNPTEAELQDMINEVDADGNGTI^64^ |
|  | E^15^AFSLFDKDGDGTITTK^31^ | E^15^A^16^ |
|  | E^128^ADIDGDGQVNYEEFVQMMTAK^149^ | E^128^ADIDGDGQV^137^ |
| **ph-QW** | S^39^LGQNPTEAELQDMINEVDADGNGTIDFPEFLTMMAR^75^ | S^39^LGQNPTEAELQDMINEVDADGNGT^63^ |
| **ph-QW**  (with competitor) | E^128^ADIDGDGQVNYEEFVQMMTAK^149^ | E^15^A^16^ |
|  | S^39^LGQNPTEAELQDMINEVDADGNGTIDFPEFLTMMAR^75^ | A^47^ELQDMINEVDADGNGTI^64^ |
| **ph-RF** | S^39^LGQNPTEAELQDMINEVDADGNGTIDFPEFLTMMAR^75^ | F^66^ |
| **ph-RF**  (with competitor) | S^39^LGQNPTEAELQDMINEVDADGNGTIDFPEFLTMMAR^75^ | T^63^, P^44^TEAELQDMINEVDADGNGTI^64^ |
|  | L^117^TDEEVDEMIREADIDGDGQVNYEEFVQMMTAK^149^ | L^117^TDEEVDEMIREAD^130^ |
|  | E^128^ADIDGDGQVNYEEFVQMMTAK^149^ | E^128^AD^130^ |
| **ph-RW** | S^39^LGQNPTEAELQDMINEVDADGNGTIDFPEFLTMMAR^75^ | N^54^ |
| **ph-RW**  (with competitor) | L^117^TDEEVDEMIREADIDGDGQVNYEEFVQMMTAK^149^ | L^117^TDEEVDEMIREAD^130^ |
|  | S^39^LGQNPTEAELQDMINEVDADGNGTIDFPEFLTMMAR^75^ | N^54^ |
| **ph-SW** | S^39^LGQNPTEAELQDMINEVDADGNGTIDFPEFLTMMAR^75^ | N^54^EVDADGNGT^63^ |
| **ph-SW**  (with competitor) | S^39^LGQNPTEAELQDMINEVDADGNGTIDFPEFLTMMAR^75^ | S^39^LGQNPTEAELQDMINEV^56^ |
|  | E^15^AFSLFDKDGDGTITTK^31^ | E^15^A^16^ |
| **ph-TW** | S^39^LGQNPTEAELQDMINEVDADGNGTIDFPEFLTMMAR^75^ | S^39^LGQNPTEAELQDMINEVDAD^59^ |
| **ph-TW**  (with competitor) | S^39^LGQNPTEAELQDMINEVDADGNGTIDFPEFLTMMAR^75^ | S^39^LGQNPTEAELQDMINEVDADGNGTIDF^66^ |
|  | E^15^AFSLFDKDGDGTITTK^31^ | E^15^A^16^ |
|  | E^128^ADIDGDGQVNYEEFVQMMTAK^149^ | E^128^ADIDGDGQV^137^ |
| **ph-VW** | S^39^LGQNPTEAELQDMINEVDADGNGTIDFPEFLTMMAR^75^ | N^54^EV^56^ |
|  | E^15^AFSLFDKDGDGTITTK^31^ | E^15^A^16^ |
| **ph-VW**  (with competitor) | S^39^LGQNPTEAELQDMINEVDADGNGTIDFPEFLTMMAR^75^ | N^54^EVDADGNGT^63^ |
| **ph-YF** | E^15^AFSLFDKDGDGTITTK^31^ | E^15^A^16^ |
| **ph-YF**  (with competitor) | E^128^ADIDGDGQVNYEEFVQMMTAK^149^ | E^128^A^129^ |
|  | S^39^LGQNPTEAELQDMINEVDADGNGTIDFPEFLTMMAR^75^ | Q^50^DMINE^55^ |

Precision of localization of photofoldamer binding within peptide sequence depends on MS/MS fragmentation efficiency and spectrum quality.

## Table S8. Characterization of the glutathione-protected monomers for K-Ras G12D.

| Compounds | Exact mass (Da) | Detected ions | | | Retention time (min) | Detected m/z |
| --- | --- | --- | --- | --- | --- | --- |
|  |  | [M+1H+]1+ | [M+2H+]2+ | [M+3H+]3+ |  |  |
| **QR-G** | 1352.75 | 1353.75 | 677.38 | 451.92 | 9.7 | 451.9, 677.1 |
| **LR-G** | 1337.75 | 1338.75 | 669.88 | 446.92 | 10.8 | 446.9, 669.7 |
| **WR-G** | 1410.75 | 1411.75 | 706.38 | 471.25 | 11.2 | 471.28, 706.2 |
| **QF-G** | 1343.75 | 1344.75 | 672.88 | 448.92 | 11.3 | 672.6, 1343.5 |
| **QW-G** | 1382.75 | 1383.75 | 692.38 | 461.92 | 11.6 | 692.1, 1382.4 |
| **WQ-G** | 1382.75 | 1383.75 | 692.38 | 461.92 | 12.4 | 692.1, 1382.4 |
| **VF-G** | 1314.65 | 1315.65 | 658.33 | 439.22 | 13.8 | 658.1, 1314.5 |
| **VW-G** | 1353.65 | 1354.65 | 677.83 | 452.22 | 14.1 | 677.6, 1353.5 |

## Table S9. Protein-induced amplification factors (PIAFs) of the building blocks in steady state measured with different power densities

| Dissipative system (5.1 mW cm^-2^) | | | |  | Dissipative system (3.8 mW cm^-2^) | | | |  | Dissipative system (2.55 mW cm^-2^) | | | |
| --- | --- | --- | --- | --- | --- | --- | --- | --- | --- | --- | --- | --- | --- |
| # | Compound | mean PIAF | SD |  | # | Compound | mean PIAF | SD |  | # | Compound | mean PIAF | SD |
| 1 | **WW-RF** | 23.64 | 5.41 |  | 1 | **WF-RW** | 14.07 | 2.87 |  | 1 | **RW-KW** | 8.79 | 0.76 |
| 2 | **WF-RW** | 22.46 | 5.22 |  | 2 | **WW-RF** | 14.07 | 2.87 |  | 2 | **VW-QW** | 6.99 | 0.87 |
| 3 | **WF-RF** | 15.26 | 2.65 |  | 3 | **WF-KW** | 12.83 | 5.04 |  | 3 | **WF-QW** | 6.79 | 2.1 |
| 4 | **WF-KW** | 14.49 | 2.08 |  | 4 | **RW-LW** | 12.3 | 1.71 |  | 4 | **RF-TW** | 4.78 | 0.25 |
| 5 | **KW-LW** | 12.53 | 0.95 |  | 5 | **KW-LW** | 12.16 | 1.98 |  | 5 | **YF-YF** | 4.64 | 0.96 |
| 6 | **RF-IF** | 10.21 | 1.33 |  | 6 | **WF-RF** | 11.6 | 2.63 |  | 6 | **KW-SW** | 2.8 | 0.15 |
| 7 | **RW-LW** | 9.78 | 1.03 |  | 7 | **RW-VW** | 8.1 | 0.81 |  | 7 | **RW-LW** | 2.66 | 1.04 |
| 8 | **RW-IF** | 8.99 | 0.77 |  | 8 | **LW-QW** | 7.35 | 1.57 |  | 8 | **RF-QW** | 2.57 | 0.13 |
| 9 | **RF-LW** | 8.88 | 0.77 |  | 9 | **WF-SW** | 7.27 | 1.61 |  | 9 | **RF-SW** | 2.55 | 0.23 |
| 10 | **RW-VW** | 7.47 | 0.72 |  | 10 | **RF-IF** | 7.05 | 1.24 |  | 10 | **YF-KW** | 2.26 | 0.3 |
| 11 | **RF-VW** | 7.1 | 0.72 |  | 11 | **YF-RW** | 7 | 0.83 |  | 11 | **WW-RF** | 2.24 | 0.07 |
| 12 | **WF-SW** | 6.99 | 0.36 |  | 12 | **RF-VW** | 6.96 | 0.77 |  | 12 | **RF-LW** | 2.09 | 0.19 |
| 13 | **YF-RW** | 6.86 | 0.88 |  | 13 | **WF-TW** | 6.88 | 1.62 |  | 13 | **RW-SW** | 2.08 | 0.15 |
| 14 | **WF-TW** | 5.91 | 0.68 |  | 14 | **RF-LW** | 6.56 | 0.71 |  | 14 | **WF-G** | 2.07 | 0.03 |
| 15 | **LW-QW** | 5.47 | 0.91 |  | 15 | **RW-IF** | 6.56 | 0.71 |  | 15 | **TW-QW** | 2.06 | 0.42 |
| 16 | **WF-VW** | 5.43 | 0.4 |  | 16 | **YF-KW** | 6.49 | 0.46 |  | 16 | **KW-QW** | 2 | 0.24 |
| 17 | **WF-QW** | 5.41 | 1.38 |  | 17 | **WF-QW** | 6.47 | 1.5 |  | 17 | **RW-TW** | 2 | 0.24 |
| 18 | **YF-RF** | 5.28 | 0.61 |  | 18 | **LW-SW** | 6.06 | 1.28 |  | 18 | **WF-RF** | 1.98 | 0.15 |
| 19 | **LW-TW** | 4.96 | 0.73 |  | 19 | **YF-TW** | 5.93 | 1.54 |  | 19 | **KW-IF** | 1.89 | 0.18 |
| 20 | **KW-IF** | 4.87 | 0.7 |  | 20 | **LW-TW** | 5.93 | 1.3 |  | 20 | **TW-TW** | 1.89 | 0.18 |
| 21 | **WW-SW** | 4.56 | 0.63 |  | 21 | **YF-RF** | 5.59 | 0.28 |  | 21 | **RF-VW** | 1.74 | 0.2 |
| 22 | **LW-SW** | 4.49 | 0.3 |  | 22 | **WW-SW** | 5.51 | 0.05 |  | 22 | **TW-SW** | 1.71 | 0.31 |
| 23 | **VW-TW** | 4.34 | 0.49 |  | 23 | **YF-SW** | 5.27 | 1.35 |  | 23 | **WW-KW** | 1.7 | 0.19 |
| 24 | **IF-QW** | 4.34 | 0.5 |  | 24 | **VW-TW** | 5.06 | 0.92 |  | 24 | **WW-TW** | 1.67 | 0.35 |
| 25 | **YF-KW** | 4.32 | 0.51 |  | 25 | **KW-IF** | 4.95 | 0.3 |  | 25 | **YF-TW** | 1.66 | 0.41 |
| 26 | **IF-SW** | 4.07 | 0.57 |  | 26 | **TW-TW** | 4.95 | 0.3 |  | 26 | **KW-LW** | 1.46 | 0.04 |
| 27 | **IF-TW** | 4.05 | 0.36 |  | 27 | **VW-SW** | 4.94 | 0.56 |  | 27 | **SW-SW** | 1.44 | 0.71 |
| 28 | **WF-YF** | 3.99 | 2.38 |  | 28 | **WF-VW** | 4.71 | 1.54 |  | 28 | **IF-G** | 1.43 | 0.01 |
| 29 | **VW-SW** | 3.92 | 0.39 |  | 29 | **IF-SW** | 4.63 | 0.8 |  | 29 | **YF-RF** | 1.42 | 0.16 |
| 30 | **YF-IF** | 3.88 | 0.37 |  | 30 | **KW-VW** | 4.48 | 0.17 |  | 30 | **VW-G** | 1.33 | 0 |
| 31 | **TW-TW** | 3.79 | 0.74 |  | 31 | **IF-TW** | 4.45 | 0.95 |  | 31 | **LW-G** | 1.3 | 0.01 |
| 32 | **YF-SW** | 3.68 | 0.68 |  | 32 | **YF-QW** | 4.44 | 0.8 |  | 32 | **YF-G** | 1.3 | 0.02 |
| 33 | **WF-IF** | 3.51 | 0.52 |  | 33 | **IF-QW** | 4.23 | 0.67 |  | 33 | **WW-RW** | 1.29 | 0.15 |
| 34 | **YF-VW** | 3.38 | 0.6 |  | 34 | **VW-QW** | 4.23 | 0.57 |  | 34 | **RW-IF** | 1.26 | 0.27 |
| 35 | **YF-QW** | 3.19 | 0.89 |  | 35 | **WF-YF** | 4.2 | 1.55 |  | 35 | **YF-RW** | 1.2 | 0.26 |
| 36 | **WF-LW** | 3.17 | 0.56 |  | 36 | **VW-VW** | 4.19 | 1.53 |  | 36 | **WF-LW** | 1.2 | 0.34 |
| 37 | **YF-LW** | 3.03 | 0.52 |  | 37 | **WW-WW** | 4.09 | 0.17 |  | 37 | **WW-IF** | 1.2 | 0.34 |
| 38 | **VW-VW** | 2.97 | 0.77 |  | 38 | **WF-IF** | 4.08 | 1.6 |  | 38 | **KW-VW** | 1.14 | 0.21 |
| 39 | **IF-IF** | 2.95 | 0.43 |  | 39 | **YF-YF** | 4.05 | 1.76 |  | 39 | **KW-TW** | 1.14 | 0.05 |
| 40 | **YF-TW** | 2.93 | 0.24 |  | 40 | **WW-IF** | 4 | 1.3 |  | 40 | **WW-QW** | 1.11 | 0.19 |
| 41 | **LW-LW** | 2.85 | 0.59 |  | 41 | **YF-VW** | 3.97 | 1.59 |  | 41 | **RW-RF** | 1.1 | 0.13 |
| 42 | **WF-WW** | 2.82 | 0.53 |  | 42 | **WF-LW** | 3.93 | 1.54 |  | 42 | **KW-RF** | 1.08 | 0.03 |
| 43 | **IF-VW** | 2.82 | 0.52 |  | 43 | **WF-WW** | 3.89 | 1.05 |  | 43 | **WW-SW** | 1.05 | 0.09 |
| 44 | **WF-WF** | 2.74 | 0.61 |  | 44 | **LW-VW** | 3.89 | 1.29 |  | 44 | **YF-SW** | 1.04 | 0.07 |
| 45 | **TW-QW** | 2.67 | 0.53 |  | 45 | **WF-WF** | 3.84 | 1.42 |  | 45 | **WF-IF** | 1.01 | 0.02 |
| 46 | **VW-QW** | 2.65 | 0.24 |  | 46 | **WW-TW** | 3.83 | 0.04 |  | 46 | **IF-IF** | 1.00* | 0 |
| 47 | **KW-VW** | 2.59 | 0.58 |  | 47 | **WW-YF** | 3.81 | 0.78 |  | 47 | **IF-QW** | 1.00* | 0 |
| 48 | **WW-TW** | 2.54 | 0.79 |  | 48 | **WW-LW** | 3.73 | 1.24 |  | 48 | **IF-SW** | 1.00* | 0 |
| 49 | **RW-RW** | 2.53 | 0.42 |  | 49 | **WW-RW** | 3.7 | 0.62 |  | 49 | **IF-TW** | 1.00* | 0 |
| 50 | **LW-VW** | 2.45 | 0.36 |  | 50 | **WW-VW** | 3.66 | 0.79 |  | 50 | **IF-VW** | 1.00* | 0 |
| 51 | **LW-IF** | 2.24 | 0.42 |  | 51 | **RW-RW** | 3.61 | 0.36 |  | 51 | **LW-IF** | 1.00* | 0 |
| 52 | **WW-IF** | 2.19 | 0.57 |  | 52 | **YF-IF** | 3.57 | 1.46 |  | 52 | **LW-LW** | 1.00* | 0 |
| 53 | **WW-LW** | 2.14 | 0.36 |  | 53 | **TW-QW** | 3.43 | 0.1 |  | 53 | **LW-QW** | 1.00* | 0 |
| 54 | **YF-YF** | 2.09 | 0.09 |  | 54 | **LW-LW** | 3.41 | 1.42 |  | 54 | **LW-SW** | 1.00* | 0 |
| 55 | **WW-VW** | 1.95 | 0.29 |  | 55 | **YF-LW** | 3.4 | 1.32 |  | 55 | **LW-TW** | 1.00* | 0 |
| 56 | **WW-RW** | 1.91 | 0.69 |  | 56 | **IF-VW** | 3.38 | 1.31 |  | 56 | **LW-VW** | 1.00* | 0 |
| 57 | **WW-YF** | 1.88 | 0.91 |  | 57 | **IF-IF** | 3.09 | 1.23 |  | 57 | **QW-QW** | 1.00* | 0 |
| 58 | **LW-G** | 1.78 | 0.46 |  | 58 | **LW-IF** | 2.86 | 1.05 |  | 58 | **RW-QW** | 1.00* | 0 |
| 59 | **RF-TW** | 1.72 | 0.34 |  | 59 | **RW-TW** | 2.7 | 0.08 |  | 59 | **RW-RW** | 1.00* | 0 |
| 60 | **RW-RF** | 1.69 | 0.06 |  | 60 | **WW-QW** | 2.61 | 0.1 |  | 60 | **RW-VW** | 1.00* | 0 |
| 61 | **QW-QW** | 1.63 | 0.09 |  | 61 | **TW-SW** | 2.16 | 0.09 |  | 61 | **VW-SW** | 1.00* | 0 |
| 62 | **WW-WW** | 1.61 | 0.1 |  | 62 | **RW-KW** | 2.14 | 0.03 |  | 62 | **VW-TW** | 1.00* | 0 |
| 63 | **SW-QW** | 1.6 | 0.15 |  | 63 | **RW-SW** | 2.11 | 0.19 |  | 63 | **VW-VW** | 1.00* | 0 |
| 64 | **TW-SW** | 1.57 | 0.11 |  | 64 | **WW-KW** | 2.1 | 0.06 |  | 64 | **WF-KW** | 1.00* | 0 |
| 65 | **RW-SW** | 1.52 | 0.1 |  | 65 | **RF-TW** | 2.05 | 0 |  | 65 | **WF-RW** | 1.00* | 0 |
| 66 | **RF-RF** | 1.52 | 0.1 |  | 66 | **RW-QW** | 2.02 | 0.12 |  | 66 | **WF-SW** | 1.00* | 0 |
| 67 | **SW-SW** | 1.48 | 0.14 |  | 67 | **KW-TW** | 1.96 | 0.15 |  | 67 | **WF-TW** | 1.00* | 0 |
| 68 | **RW-KW** | 1.46 | 0.03 |  | 68 | **SW-QW** | 1.88 | 0.19 |  | 68 | **WF-VW** | 1.00* | 0 |
| 69 | **KW-RF** | 1.46 | 0.06 |  | 69 | **SW-SW** | 1.86 | 0.02 |  | 69 | **WF-WF** | 1.00* | 0 |
| 70 | **RF-QW** | 1.39 | 0.08 |  | 70 | **QW-QW** | 1.85 | 0.15 |  | 70 | **WF-WW** | 1.00* | 0 |
| 71 | **RW-TW** | 1.36 | 0.06 |  | 71 | **RW-RF** | 1.74 | 0.13 |  | 71 | **WF-YF** | 1.00* | 0 |
| 72 | **RW-QW** | 1.35 | 0.47 |  | 72 | **RF-RF** | 1.69 | 0.03 |  | 72 | **WW-LW** | 1.00* | 0 |
| 73 | **YF-G** | 1.25 | 0.05 |  | 73 | **WF-G** | 1.66 | 0.05 |  | 73 | **WW-VW** | 1.00* | 0 |
| 74 | **RF-SW** | 1.22 | 0.4 |  | 74 | **RF-SW** | 1.58 | 0.09 |  | 74 | **WW-WW** | 1.00* | 0 |
| 75 | **KW-TW** | 1.18 | 0.2 |  | 75 | **KW-SW** | 1.52 | 0.09 |  | 75 | **WW-YF** | 1.00* | 0 |
| 76 | **WW-KW** | 1.17 | 0.06 |  | 76 | **KW-RF** | 1.47 | 0.11 |  | 76 | **YF-IF** | 1.00* | 0 |
| 77 | **KW-KW** | 1.13 | 0.1 |  | 77 | **LW-G** | 1.45 | 0.31 |  | 77 | **YF-LW** | 1.00* | 0 |
| 78 | **KW-SW** | 1.11 | 0.09 |  | 78 | **RF-QW** | 1.45 | 0.02 |  | 78 | **YF-QW** | 1.00* | 0 |
| 79 | **KW-QW** | 1.09 | 0.09 |  | 79 | **KW-KW** | 1.41 | 0.07 |  | 79 | **YF-VW** | 1.00* | 0 |
| 80 | **WW-QW** | 1.06 | 0.33 |  | 80 | **KW-QW** | 1.39 | 0.13 |  | 80 | **TW-G** | 0.98 | 0.02 |
| 81 | **QW-G** | 1.06 | 0.05 |  | 81 | **IF-G** | 1.36 | 0.02 |  | 81 | **QW-G** | 0.96 | 0.01 |
| 82 | **SW-G** | 1.03 | 0.06 |  | 82 | **YF-G** | 1.31 | 0 |  | 82 | **SW-G** | 0.96 | 0.01 |
| 83 | **WW-G** | 1 | 0.04 |  | 83 | **VW-G** | 1.23 | 0.02 |  | 83 | **KW-KW** | 0.96 | 0.09 |
| 84 | **TW-G** | 0.99 | 0.19 |  | 84 | **TW-G** | 1.02 | 0.04 |  | 84 | **RW-G** | 0.95 | 0.02 |
| 85 | **IF-G** | 0.93 | 0.18 |  | 85 | **WW-G** | 0.99 | 0.08 |  | 85 | **RF-G** | 0.95 | 0.01 |
| 86 | **WF-G** | 0.9 | 0.38 |  | 86 | **SW-G** | 0.97 | 0.05 |  | 86 | **RF-RF** | 0.94 | 0.01 |
| 87 | **VW-G** | 0.8 | 0.16 |  | 87 | **QW-G** | 0.93 | 0.08 |  | 87 | **KW-G** | 0.93 | 0.01 |
| 88 | **KW-G** | 0.75 | 0.06 |  | 88 | **RF-G** | 0.92 | 0 |  | 88 | **WW-G** | 0.88 | 0.02 |
| 89 | **RF-G** | 0.74 | 0.04 |  | 89 | **KW-G** | 0.9 | 0.01 |  | 89 | **SW-QW** | 0.75 | 0.19 |
| 90 | **RW-G** | 0.66 | 0.05 |  | 90 | **RW-G** | 0.89 | 0.02 |  | 90 | **RF-IF** | 0.37 | 0.02 |

*amount of building block was under the detection limit; minimum detectable AUC (5E+05 AU) was used for calculations.

Gluthatione is indicated with –G after the two-letter code of the foldamer.

## Table S10. Protein-induced amplification factors of the building blocks in steady state as a function of CaM concentration

| **1 μM CaM** | | | |  | **6 μM CaM** | | | |  | **12 μM CaM** | | | |
| --- | --- | --- | --- | --- | --- | --- | --- | --- | --- | --- | --- | --- | --- |
| # | Compound | mean PIAF | SD |  | # | Compound | mean PIAF | SD |  | # | Compound | mean PIAF | SD |
| 1 | **RF-VW** | 4.5 | 0.19 |  | 1 | **WW-RF** | 23.64 | 5.41 |  | 1 | **WF-KW** | 16.4 | 0.92 |
| 2 | **RF-IF** | 4.08 | 1.06 |  | 2 | **WF-RW** | 22.46 | 5.22 |  | 2 | **WF-RW** | 16.1 | 1.1 |
| 3 | **RW-LW** | 3.64 | 1.46 |  | 3 | **WF-RF** | 15.26 | 2.65 |  | 3 | **WW-RF** | 16.1 | 1.1 |
| 4 | **WF-RF** | 3.41 | 1.21 |  | 4 | **WF-KW** | 14.49 | 2.08 |  | 4 | **KW-LW** | 13.56 | 0.93 |
| 5 | **WF-RW** | 3.3 | 0.99 |  | 5 | **KW-LW** | 12.53 | 0.95 |  | 5 | **RW-LW** | 13.51 | 0.49 |
| 6 | **WW-RF** | 3.3 | 0.99 |  | 6 | **RF-IF** | 10.21 | 1.33 |  | 6 | **WF-RF** | 13.47 | 0.97 |
| 7 | **KW-LW** | 3.21 | 1.14 |  | 7 | **RW-LW** | 9.78 | 1.03 |  | 7 | **RW-VW** | 8.67 | 0.5 |
| 8 | **RW-VW** | 3.14 | 0.71 |  | 8 | **RW-IF** | 8.99 | 0.77 |  | 8 | **LW-QW** | 8.46 | 0.39 |
| 9 | **IF-SW** | 2.9 | 0.87 |  | 9 | **RF-LW** | 8.88 | 0.77 |  | 9 | **WF-SW** | 8.41 | 0.6 |
| 10 | **IF-TW** | 2.89 | 0.94 |  | 10 | **RW-VW** | 7.47 | 0.72 |  | 10 | **WF-TW** | 8.02 | 0.57 |
| 11 | **IF-QW** | 2.86 | 0.87 |  | 11 | **RF-VW** | 7.1 | 0.72 |  | 11 | **RF-IF** | 7.92 | 0.39 |
| 12 | **VW-TW** | 2.83 | 0.97 |  | 12 | **WF-SW** | 6.99 | 0.36 |  | 12 | **YF-RW** | 7.59 | 0.54 |
| 13 | **WF-KW** | 2.82 | 0.79 |  | 13 | **YF-RW** | 6.86 | 0.88 |  | 13 | **WF-QW** | 7.54 | 0.25 |
| 14 | **VW-SW** | 2.74 | 1.01 |  | 14 | **WF-TW** | 5.91 | 0.68 |  | 14 | **RF-VW** | 7.5 | 0.6 |
| 15 | **RF-LW** | 2.71 | 0.52 |  | 15 | **LW-QW** | 5.47 | 0.91 |  | 15 | **RF-LW** | 7.06 | 0.49 |
| 16 | **YF-RW** | 2.71 | 0.69 |  | 16 | **WF-VW** | 5.43 | 0.4 |  | 16 | **RW-IF** | 7.06 | 0.49 |
| 17 | **RW-IF** | 2.69 | 0.44 |  | 17 | **WF-QW** | 5.41 | 1.38 |  | 17 | **YF-TW** | 7.02 | 0.53 |
| 18 | **IF-IF** | 2.67 | 0.71 |  | 18 | **YF-RF** | 5.28 | 0.61 |  | 18 | **LW-SW** | 6.97 | 0.32 |
| 19 | **YF-RF** | 2.66 | 0.33 |  | 19 | **LW-TW** | 4.96 | 0.73 |  | 19 | **LW-TW** | 6.85 | 0.35 |
| 20 | **VW-VW** | 2.65 | 0.72 |  | 20 | **KW-IF** | 4.87 | 0.7 |  | 20 | **YF-KW** | 6.81 | 0.59 |
| 21 | **LW-QW** | 2.6 | 0.79 |  | 21 | **WW-SW** | 4.56 | 0.63 |  | 21 | **YF-SW** | 6.23 | 0.48 |
| 22 | **LW-SW** | 2.58 | 0.84 |  | 22 | **LW-SW** | 4.49 | 0.3 |  | 22 | **WF-VW** | 5.8 | 0.34 |
| 23 | **YF-TW** | 2.57 | 0.7 |  | 23 | **VW-TW** | 4.34 | 0.49 |  | 23 | **YF-RF** | 5.79 | 0.52 |
| 24 | **IF-VW** | 2.54 | 0.76 |  | 24 | **IF-QW** | 4.34 | 0.5 |  | 24 | **VW-TW** | 5.7 | 0.31 |
| 25 | **VW-QW** | 2.53 | 0.47 |  | 25 | **YF-KW** | 4.32 | 0.51 |  | 25 | **WW-SW** | 5.54 | 0.3 |
| 26 | **LW-TW** | 2.53 | 0.88 |  | 26 | **IF-SW** | 4.07 | 0.57 |  | 26 | **VW-SW** | 5.33 | 0.3 |
| 27 | **YF-QW** | 2.51 | 0.71 |  | 27 | **IF-TW** | 4.05 | 0.36 |  | 27 | **WF-YF** | 5.3 | 0.29 |
| 28 | **YF-IF** | 2.5 | 0.68 |  | 28 | **WF-YF** | 3.99 | 2.38 |  | 28 | **YF-YF** | 5.29 | 0.33 |
| 29 | **WF-SW** | 2.48 | 0.91 |  | 29 | **VW-SW** | 3.92 | 0.39 |  | 29 | **VW-VW** | 5.27 | 0.28 |
| 30 | **WF-QW** | 2.47 | 0.8 |  | 30 | **YF-IF** | 3.88 | 0.37 |  | 30 | **WF-IF** | 5.21 | 0.24 |
| 31 | **YF-YF** | 2.44 | 0.64 |  | 31 | **TW-TW** | 3.79 | 0.74 |  | 31 | **IF-SW** | 5.19 | 0.31 |
| 32 | **YF-VW** | 2.42 | 0.71 |  | 32 | **YF-SW** | 3.68 | 0.68 |  | 32 | **KW-IF** | 5.17 | 0.16 |
| 33 | **WW-LW** | 2.34 | 0.75 |  | 33 | **WF-IF** | 3.51 | 0.52 |  | 33 | **TW-TW** | 5.17 | 0.16 |
| 34 | **YF-KW** | 2.32 | 0.39 |  | 34 | **YF-VW** | 3.38 | 0.6 |  | 34 | **IF-TW** | 5.12 | 0.42 |
| 35 | **LW-IF** | 2.32 | 0.56 |  | 35 | **YF-QW** | 3.19 | 0.89 |  | 35 | **YF-VW** | 5.09 | 0.32 |
| 36 | **LW-VW** | 2.31 | 0.52 |  | 36 | **WF-LW** | 3.17 | 0.56 |  | 36 | **WF-LW** | 5.02 | 0.25 |
| 37 | **YF-SW** | 2.31 | 0.29 |  | 37 | **YF-LW** | 3.03 | 0.52 |  | 37 | **YF-QW** | 5 | 0.1 |
| 38 | **YF-LW** | 2.3 | 0.56 |  | 38 | **VW-VW** | 2.97 | 0.77 |  | 38 | **WW-IF** | 4.92 | 0.23 |
| 39 | **LW-LW** | 2.26 | 0.5 |  | 39 | **IF-IF** | 2.95 | 0.43 |  | 39 | **WF-WF** | 4.85 | 0.19 |
| 40 | **WF-TW** | 2.24 | 0.66 |  | 40 | **YF-TW** | 2.93 | 0.24 |  | 40 | **LW-VW** | 4.8 | 0.32 |
| 41 | **WF-IF** | 2.21 | 0.48 |  | 41 | **LW-LW** | 2.85 | 0.59 |  | 41 | **IF-QW** | 4.7 | 0.2 |
| 42 | **WF-VW** | 2.2 | 0.47 |  | 42 | **WF-WW** | 2.82 | 0.53 |  | 42 | **WF-WW** | 4.63 | 0.3 |
| 43 | **TW-TW** | 2.13 | 0.53 |  | 43 | **IF-VW** | 2.82 | 0.52 |  | 43 | **VW-QW** | 4.63 | 0.09 |
| 44 | **WF-WW** | 2.13 | 0.61 |  | 44 | **WF-WF** | 2.74 | 0.61 |  | 44 | **WW-LW** | 4.61 | 0.21 |
| 45 | **WF-YF** | 2.12 | 0.47 |  | 45 | **TW-QW** | 2.67 | 0.53 |  | 45 | **YF-IF** | 4.61 | 0.21 |
| 46 | **WW-IF** | 2.09 | 0.37 |  | 46 | **VW-QW** | 2.65 | 0.24 |  | 46 | **LW-LW** | 4.42 | 0.2 |
| 47 | **WF-WF** | 2.09 | 0.45 |  | 47 | **KW-VW** | 2.59 | 0.58 |  | 47 | **WW-YF** | 4.36 | 0.44 |
| 48 | **KW-IF** | 2.08 | 0.51 |  | 48 | **WW-TW** | 2.54 | 0.79 |  | 48 | **KW-VW** | 4.36 | 0.37 |
| 49 | **WF-LW** | 2.07 | 0.42 |  | 49 | **RW-RW** | 2.53 | 0.42 |  | 49 | **YF-LW** | 4.33 | 0.24 |
| 50 | **KW-VW** | 1.97 | 0.21 |  | 50 | **LW-VW** | 2.45 | 0.36 |  | 50 | **IF-VW** | 4.3 | 0.22 |
| 51 | **WW-YF** | 1.83 | 0.55 |  | 51 | **LW-IF** | 2.24 | 0.42 |  | 51 | **WW-VW** | 4.22 | 0.39 |
| 52 | **WW-VW** | 1.82 | 0.49 |  | 52 | **WW-IF** | 2.19 | 0.57 |  | 52 | **WW-WW** | 3.97 | 1.97 |
| 53 | **RW-RF** | 1.65 | 0.32 |  | 53 | **WW-LW** | 2.14 | 0.36 |  | 53 | **IF-IF** | 3.95 | 0.21 |
| 54 | **QW-QW** | 1.65 | 0.26 |  | 54 | **YF-YF** | 2.09 | 0.09 |  | 54 | **WW-TW** | 3.86 | 0.28 |
| 55 | **RW-RW** | 1.55 | 0.43 |  | 55 | **WW-VW** | 1.95 | 0.29 |  | 55 | **LW-IF** | 3.6 | 0.22 |
| 56 | **TW-SW** | 1.53 | 0.33 |  | 56 | **WW-RW** | 1.91 | 0.69 |  | 56 | **TW-QW** | 3.36 | 0.13 |
| 57 | **SW-QW** | 1.53 | 0.31 |  | 57 | **WW-YF** | 1.88 | 0.91 |  | 57 | **RW-RW** | 3.36 | 0.07 |
| 58 | **RF-QW** | 1.51 | 0.26 |  | 58 | **LW-G** | 1.78 | 0.46 |  | 58 | **WW-RW** | 3.26 | 0.07 |
| 59 | **RF-TW** | 1.5 | 0.24 |  | 59 | **RF-TW** | 1.72 | 0.34 |  | 59 | **RW-TW** | 2.76 | 0.08 |
| 60 | **WW-SW** | 1.5 | 0.15 |  | 60 | **RW-RF** | 1.69 | 0.06 |  | 60 | **WW-QW** | 2.54 | 0.28 |
| 61 | **RW-QW** | 1.5 | 0.29 |  | 61 | **QW-QW** | 1.63 | 0.09 |  | 61 | **WW-KW** | 2.15 | 0.11 |
| 62 | **WW-WW** | 1.48 | 0.45 |  | 62 | **WW-WW** | 1.61 | 0.1 |  | 62 | **RW-KW** | 2.12 | 0.06 |
| 63 | **TW-QW** | 1.47 | 0.25 |  | 63 | **SW-QW** | 1.6 | 0.15 |  | 63 | **TW-SW** | 2.09 | 0.1 |
| 64 | **SW-SW** | 1.44 | 0.23 |  | 64 | **TW-SW** | 1.57 | 0.11 |  | 64 | **RF-TW** | 2.05 | 0.06 |
| 65 | **RF-SW** | 1.43 | 0.13 |  | 65 | **RW-SW** | 1.52 | 0.1 |  | 65 | **RW-SW** | 1.97 | 0.03 |
| 66 | **KW-RF** | 1.42 | 0.26 |  | 66 | **RF-RF** | 1.52 | 0.1 |  | 66 | **RW-QW** | 1.93 | 0.02 |
| 67 | **RW-SW** | 1.4 | 0.33 |  | 67 | **SW-SW** | 1.48 | 0.14 |  | 67 | **SW-SW** | 1.88 | 0.02 |
| 68 | **RW-KW** | 1.34 | 0.3 |  | 68 | **RW-KW** | 1.46 | 0.03 |  | 68 | **KW-TW** | 1.85 | 0.02 |
| 69 | **KW-TW** | 1.33 | 0.16 |  | 69 | **KW-RF** | 1.46 | 0.06 |  | 69 | **QW-QW** | 1.75 | 0.03 |
| 70 | **KW-KW** | 1.32 | 0.18 |  | 70 | **RF-QW** | 1.39 | 0.08 |  | 70 | **SW-QW** | 1.74 | 0.02 |
| 71 | **KW-SW** | 1.32 | 0.18 |  | 71 | **RW-TW** | 1.36 | 0.06 |  | 71 | **RF-RF** | 1.72 | 0.08 |
| 72 | **KW-QW** | 1.29 | 0.2 |  | 72 | **RW-QW** | 1.35 | 0.47 |  | 72 | **RW-RF** | 1.65 | 0.03 |
| 73 | **RW-TW** | 1.28 | 0.32 |  | 73 | **YF-G** | 1.25 | 0.05 |  | 73 | **RF-SW** | 1.64 | 0.01 |
| 74 | **WW-RW** | 1.25 | 0.44 |  | 74 | **RF-SW** | 1.22 | 0.4 |  | 74 | **WF-G** | 1.63 | 0.07 |
| 75 | **WW-TW** | 1.21 | 0.39 |  | 75 | **KW-TW** | 1.18 | 0.2 |  | 75 | **LW-G** | 1.47 | 0.1 |
| 76 | **RF-RF** | 1.17 | 0.04 |  | 76 | **WW-KW** | 1.17 | 0.06 |  | 76 | **KW-SW** | 1.45 | 0.01 |
| 77 | **WW-QW** | 1.12 | 0.3 |  | 77 | **KW-KW** | 1.13 | 0.1 |  | 77 | **RF-QW** | 1.44 | 0.04 |
| 78 | **WW-KW** | 1.09 | 0.43 |  | 78 | **KW-SW** | 1.11 | 0.09 |  | 78 | **KW-RF** | 1.4 | 0.12 |
| 79 | **SW-G** | 0.93 | 0.05 |  | 79 | **KW-QW** | 1.09 | 0.09 |  | 79 | **KW-KW** | 1.37 | 0.19 |
| 80 | **LW-G** | 0.93 | 0.05 |  | 80 | **WW-QW** | 1.06 | 0.33 |  | 80 | **IF-G** | 1.34 | 0.02 |
| 81 | **RF-G** | 0.92 | 0.04 |  | 81 | **QW-G** | 1.06 | 0.05 |  | 81 | **YF-G** | 1.32 | 0 |
| 82 | **KW-G** | 0.9 | 0 |  | 82 | **SW-G** | 1.03 | 0.06 |  | 82 | **KW-QW** | 1.3 | 0.05 |
| 83 | **IF-G** | 0.9 | 0.02 |  | 83 | **WW-G** | 1 | 0.04 |  | 83 | **VW-G** | 1.24 | 0.06 |
| 84 | **YF-G** | 0.89 | 0.07 |  | 84 | **TW-G** | 0.99 | 0.19 |  | 84 | **SW-G** | 1.01 | 0.01 |
| 85 | **QW-G** | 0.89 | 0.06 |  | 85 | **IF-G** | 0.93 | 0.18 |  | 85 | **TW-G** | 0.99 | 0.06 |
| 86 | **RW-G** | 0.88 | 0.04 |  | 86 | **WF-G** | 0.9 | 0.38 |  | 86 | **QW-G** | 0.99 | 0.01 |
| 87 | **TW-G** | 0.85 | 0.03 |  | 87 | **VW-G** | 0.8 | 0.16 |  | 87 | **WW-G** | 0.94 | 0.04 |
| 88 | **WF-G** | 0.85 | 0.05 |  | 88 | **KW-G** | 0.75 | 0.06 |  | 88 | **RF-G** | 0.92 | 0.06 |
| 89 | **VW-G** | 0.83 | 0.04 |  | 89 | **RF-G** | 0.74 | 0.04 |  | 89 | **KW-G** | 0.89 | 0 |
| 90 | **WW-G** | 0.75 | 0.08 |  | 90 | **RW-G** | 0.66 | 0.05 |  | 90 | **RW-G** | 0.88 | 0.04 |

Gluthatione is indicated with –G after the two-letter code of the foldamer.

## Table S11. Protein-induced amplification factors (PIAFs) of the building blocks in equilibrium

| Equilibrium | | | |
| --- | --- | --- | --- |
| # | Compound | mean PIAF | SD |
| 1 | WF-RW | 13.99 | 1.66 |
| 2 | WF-KW | 12.78 | 2.43 |
| 3 | WW-KW | 12.01 | 1.35 |
| 4 | WF-RF | 11.79 | 1.22 |
| 5 | WW-RW | 11.57 | 1.32 |
| 6 | WW-RF | 11.44 | 1.03 |
| 7 | KW-LW | 10.60 | 1.27 |
| 8 | RW-LW | 10.38 | 0.82 |
| 9 | YF-KW | 9.64 | 1.29 |
| 10 | RW-VW | 9.51 | 1.42 |
| 11 | RF-LW | 9.35 | 0.96 |
| 12 | RW-IF | 9.35 | 1.25 |
| 13 | RF-VW | 9.32 | 1.25 |
| 14 | RF-IF | 9.10 | 1.3 |
| 15 | YF-RW | 9.06 | 0.68 |
| 16 | YF-RF | 8.79 | 1.05 |
| 17 | RW-RW | 8.45 | 0.67 |
| 18 | RW-KW | 8.35 | 0.99 |
| 19 | RW-RF | 8.03 | 0.31 |
| 20 | KW-VW | 7.42 | 1.02 |
| 21 | LW-SW | 7.18 | 0.45 |
| 22 | TW-TW | 6.94 | 0.6 |
| 23 | KW-KW | 6.61 | 0.42 |
| 24 | WW-QW | 6.60 | 0.66 |
| 25 | VW-QW | 6.56 | 0.08 |
| 26 | YF-YF | 6.16 | 0.14 |
| 27 | WF-TW | 6.01 | 1.38 |
| 28 | VW-SW | 5.99 | 1.32 |
| 29 | IF-TW | 5.84 | 0.57 |
| 30 | RW-SW | 5.79 | 0.84 |
| 31 | LW-TW | 5.79 | 0.38 |
| 32 | WF-SW | 5.77 | 0.39 |
| 33 | IF-IF | 5.76 | 1.06 |
| 34 | RW-TW | 5.72 | 0.14 |
| 35 | IF-SW | 5.66 | 0.05 |
| 36 | YF-TW | 5.66 | 0.31 |
| 37 | WF-QW | 5.39 | 0.52 |
| 38 | YF-IF | 5.25 | 1.16 |
| 39 | VW-TW | 5.11 | 0.45 |
| 40 | YF-SW | 5.11 | 0.42 |
| 41 | WW-TW | 5.06 | 0.15 |
| 42 | RF-TW | 4.95 | 0.21 |
| 43 | LW-LW | 4.94 | 0.96 |
| 44 | VW-VW | 4.93 | 0.1 |
| 45 | SW-SW | 4.93 | 0.55 |
| 46 | KW-IF | 4.90 | 1.34 |
| 47 | WW-SW | 4.88 | 0.41 |
| 48 | KW-TW | 4.88 | 0.12 |
| 49 | KW-RF | 4.84 | 0.2 |
| 50 | LW-IF | 4.84 | 0.63 |
| 51 | LW-QW | 4.82 | 0.35 |
| 52 | RF-RF | 4.82 | 0.36 |
| 53 | YF-QW | 4.78 | 0.84 |
| 54 | RW-QW | 4.71 | 0.7 |
| 55 | QW-QW | 4.27 | 0.47 |
| 56 | RF-SW | 4.26 | 1.29 |
| 57 | LW-VW | 4.24 | 0.22 |
| 58 | TW-QW | 4.22 | 0.06 |
| 59 | YF-VW | 4.20 | 1.26 |
| 60 | RF-QW | 4.20 | 0.04 |
| 61 | YF-LW | 4.17 | 1.46 |
| 62 | IF-VW | 4.07 | 0.49 |
| 63 | KW-SW | 4.05 | 0.28 |
| 64 | WF-VW | 4.01 | 0.4 |
| 65 | KW-QW | 3.92 | 0.2 |
| 66 | WW-IF | 3.90 | 1.11 |
| 67 | TW-SW | 3.81 | 0.22 |
| 68 | SW-QW | 3.79 | 1.04 |
| 69 | WW-YF | 3.78 | 0.9 |
| 70 | WF-LW | 3.65 | 1.04 |
| 71 | WW-WW | 3.61 | 0.67 |
| 72 | WF-WF | 3.53 | 1.58 |
| 73 | WW-VW | 3.45 | 0.62 |
| 74 | WF-IF | 3.40 | 0.31 |
| 75 | WF-YF | 3.29 | 0.4 |
| 76 | WW-LW | 3.07 | 0.49 |
| 77 | IF-QW | 3.01 | 0.51 |
| 78 | WF-WW | 3.00 | 0.55 |

## Table S12. Product distribution in the thermodynamically controlled system and in the UV-induced system

|  |  | **Thermodynamically controlled system** | | | |  | **UV-induced system** | | | | Statistical distribution |
| --- | --- | --- | --- | --- | --- | --- | --- | --- | --- | --- | --- |
|  |  | protein-free |  | protein-  containing |  |  | protein-free |  | protein-  catalyzed |  |  |
|  |  | c (µM) | SD | c (µM) | SD |  | c (µM) | SD | c (µM) | SD | c (calc) (µM) |
| 1 | IF-IF | 0.18 | 0.03 | 0.52 | 0.08 |  | 0.08 | 0.01 | 0.22 | 0.04 | *0.21* |
| 2 | IF-QW | 0.16 | 0.05 | 0.19 | 0.05 |  | 0.07 | 0.02 | 0.31 | 0.09 | *0.42* |
| 3 | IF-SW | 0.08 | 0.01 | 0.24 | 0.04 |  | 0.07 | 0.01 | 0.29 | 0.09 | *0.42* |
| 4 | IF-TW | 0.12 | 0.03 | 0.35 | 0.07 |  | 0.09 | 0.03 | 0.37 | 0.07 | *0.42* |
| 5 | IF-VW | 0.31 | 0.00 | 0.76 | 0.01 |  | 0.07 | 0.02 | 0.24 | 0.09 | *0.42* |
| 6 | KW-IF | 0.04 | 0.00 | 0.21 | 0.03 |  | 0.17 | 0.06 | 0.93 | 0.13 | *0.42* |
| 7 | KW-KW | 0.10 | 0.06 | 0.03 | 0.02 |  | 0.13 | 0.02 | 0.09 | 0.03 | *0.21* |
| 8 | KW-LW | 0.02 | 0.00 | 0.17 | 0.04 |  | 0.05 | 0.01 | 0.53 | 0.17 | *0.42* |
| 9 | KW-QW | 0.03 | 0.02 | 0.08 | 0.07 |  | 0.22 | 0.06 | 0.30 | 0.10 | *0.42* |
| 10 | KW-RF | 0.04 | 0.01 | 0.09 | 0.03 |  | 0.31 | 0.11 | 0.26 | 0.07 | *0.42* |
| 11 | KW-SW | 0.03 | 0.02 | 0.08 | 0.05 |  | 0.20 | 0.05 | 0.25 | 0.08 | *0.42* |
| 12 | KW-TW | 0.07 | 0.06 | 0.23 | 0.19 |  | 0.51 | 0.17 | 0.93 | 0.30 | *0.42* |
| 13 | KW-VW | 0.03 | 0.01 | 0.15 | 0.04 |  | 0.06 | 0.01 | 0.43 | 0.16 | *0.42* |
| 14 | LW-IF | 0.20 | 0.02 | 0.66 | 0.07 |  | 0.08 | 0.03 | 0.44 | 0.13 | *0.42* |
| 15 | LW-LW | 0.38 | 0.09 | 0.98 | 0.22 |  | 0.06 | 0.02 | 0.25 | 0.09 | *0.21* |
| 16 | LW-QW | 0.15 | 0.01 | 0.41 | 0.03 |  | 0.08 | 0.02 | 0.50 | 0.08 | *0.42* |
| 17 | LW-SW | 0.12 | 0.01 | 0.34 | 0.02 |  | 0.06 | 0.02 | 0.31 | 0.12 | *0.42* |
| 18 | LW-TW | 0.18 | 0.01 | 0.51 | 0.03 |  | 0.08 | 0.01 | 0.35 | 0.04 | *0.42* |
| 19 | LW-VW | 0.52 | 0.07 | 1.20 | 0.15 |  | 0.07 | 0.03 | 0.19 | 0.04 | *0.42* |
| 20 | QW-QW | 0.04 | 0.01 | 0.09 | 0.02 |  | 0.16 | 0.05 | 0.22 | 0.05 | *0.21* |
| 21 | RF-IF | 0.03 | 0.01 | 0.16 | 0.04 |  | 0.09 | 0.03 | 0.46 | 0.06 | *0.42* |
| 22 | RF-LW | 0.08 | 0.02 | 0.49 | 0.13 |  | 0.13 | 0.01 | 1.08 | 0.24 | *0.42* |
| 23 | RF-QW | 0.06 | 0.04 | 0.17 | 0.12 |  | 0.38 | 0.14 | 0.54 | 0.18 | *0.42* |
| 24 | RF-RF | 0.01 | 0.01 | 0.04 | 0.04 |  | 0.22 | 0.05 | 0.14 | 0.03 | *0.21* |
| 25 | RF-SW | 0.03 | 0.02 | 0.10 | 0.07 |  | 0.20 | 0.07 | 0.37 | 0.10 | *0.42* |
| 26 | RF-TW | 0.05 | 0.04 | 0.17 | 0.14 |  | 0.25 | 0.09 | 0.57 | 0.07 | *0.42* |
| 27 | RF-VW | 0.03 | 0.01 | 0.20 | 0.06 |  | 0.05 | 0.01 | 0.39 | 0.12 | *0.42* |
| 28 | RW-IF | 0.08 | 0.02 | 0.51 | 0.14 |  | 0.14 | 0.05 | 1.02 | 0.22 | *0.42* |
| 29 | RW-KW | 0.03 | 0.01 | 0.11 | 0.06 |  | 0.27 | 0.10 | 0.26 | 0.04 | *0.42* |
| 30 | RW-LW | 0.03 | 0.03 | 0.27 | 0.15 |  | 0.05 | 0.01 | 0.65 | 0.17 | *0.42* |
| 31 | RW-QW | 0.07 | 0.06 | 0.26 | 0.10 |  | 0.38 | 0.10 | 0.67 | 0.24 | *0.42* |
| 32 | RW-RF | 0.04 | 0.02 | 0.16 | 0.09 |  | 0.25 | 0.07 | 0.30 | 0.12 | *0.42* |
| 33 | RW-RW | 0.16 | 0.08 | 0.14 | 0.07 |  | 0.10 | 0.03 | 0.18 | 0.04 | *0.21* |
| 34 | RW-SW | 0.04 | 0.02 | 0.13 | 0.06 |  | 0.19 | 0.05 | 0.36 | 0.11 | *0.42* |
| 35 | RW-TW | 0.09 | 0.04 | 0.30 | 0.14 |  | 0.28 | 0.07 | 0.34 | 0.10 | *0.42* |
| 36 | RW-VW | 0.04 | 0.01 | 0.28 | 0.08 |  | 0.07 | 0.01 | 0.40 | 0.10 | *0.42* |
| 37 | SW-QW | 0.16 | 0.04 | 0.29 | 0.08 |  | 0.33 | 0.11 | 0.68 | 0.25 | *0.42* |
| 38 | SW-SW | 0.03 | 0.00 | 0.06 | 0.00 |  | 0.07 | 0.01 | 0.15 | 0.03 | *0.21* |
| 39 | TW-QW | 0.16 | 0.03 | 0.40 | 0.08 |  | 0.19 | 0.05 | 0.66 | 0.18 | *0.42* |
| 40 | TW-SW | 0.11 | 0.02 | 0.21 | 0.04 |  | 0.11 | 0.03 | 0.34 | 0.13 | *0.42* |
| 41 | TW-TW | 0.05 | 0.00 | 0.13 | 0.00 |  | 0.08 | 0.02 | 0.47 | 0.06 | *0.21* |
| 42 | VW-QW | 0.11 | 0.02 | 0.47 | 0.08 |  | 0.06 | 0.02 | 0.18 | 0.06 | *0.42* |
| 43 | VW-SW | 0.08 | 0.02 | 0.32 | 0.07 |  | 0.05 | 0.01 | 0.13 | 0.04 | *0.42* |
| 44 | VW-TW | 0.01 | 0.00 | 0.03 | 0.00 |  | 0.08 | 0.01 | 0.31 | 0.08 | *0.42* |
| 45 | VW-VW | 0.16 | 0.01 | 0.41 | 0.01 |  | 0.02 | 0.00 | 0.08 | 0.02 | *0.21* |
| 46 | WF-IF | 0.37 | 0.04 | 0.79 | 0.09 |  | 0.15 | 0.05 | 0.29 | 0.10 | *0.42* |
| 47 | WF-KW | 0.02 | 0.00 | 0.17 | 0.04 |  | 0.07 | 0.01 | 0.35 | 0.09 | *0.42* |
| 48 | WF-LW | 1.22 | 0.28 | 2.52 | 0.58 |  | 0.29 | 0.11 | 0.74 | 0.13 | *0.42* |
| 49 | WF-QW | 0.10 | 0.01 | 0.45 | 0.05 |  | 0.10 | 0.01 | 0.26 | 0.07 | *0.42* |
| 50 | WF-RF | 0.03 | 0.01 | 0.27 | 0.04 |  | 0.09 | 0.04 | 0.43 | 0.17 | *0.42* |
| 51 | WF-RW | 0.09 | 0.02 | 1.00 | 0.19 |  | 0.14 | 0.05 | 1.19 | 0.26 | *0.42* |
| 52 | WF-SW | 0.09 | 0.00 | 0.30 | 0.01 |  | 0.08 | 0.02 | 0.13 | 0.03 | *0.42* |
| 53 | WF-TW | 0.11 | 0.00 | 0.37 | 0.02 |  | 0.09 | 0.04 | 0.22 | 0.07 | *0.42* |
| 54 | WF-VW | 0.43 | 0.06 | 1.08 | 0.15 |  | 0.11 | 0.02 | 0.20 | 0.03 | *0.42* |
| 55 | WF-WF | 0.25 | 0.08 | 0.51 | 0.16 |  | 0.11 | 0.03 | 0.11 | 0.03 | *0.21* |
| 56 | WF-WW | 1.54 | 0.25 | 3.84 | 0.62 |  | 0.02 | 0.01 | 0.24 | 0.09 | *0.42* |
| 57 | WF-YF | 0.50 | 0.04 | 1.18 | 0.10 |  | 0.22 | 0.08 | 0.41 | 0.12 | *0.42* |
| 58 | WW-IF | 1.00 | 0.18 | 2.23 | 0.41 |  | 0.34 | 0.11 | 0.33 | 0.05 | *0.42* |
| 59 | WW-KW | 0.04 | 0.01 | 0.34 | 0.07 |  | 0.07 | 0.02 | 0.26 | 0.05 | *0.42* |
| 60 | WW-LW | 1.40 | 0.25 | 2.85 | 0.51 |  | 0.06 | 0.01 | 0.35 | 0.08 | *0.42* |
| 61 | WW-QW | 0.23 | 0.02 | 0.93 | 0.06 |  | 0.09 | 0.01 | 0.25 | 0.06 | *0.42* |
| 62 | WW-RF | 0.06 | 0.01 | 0.43 | 0.06 |  | 0.12 | 0.04 | 1.24 | 0.12 | *0.42* |
| 63 | WW-RW | 0.10 | 0.02 | 0.83 | 0.15 |  | 0.07 | 0.01 | 0.34 | 0.14 | *0.42* |
| 64 | WW-SW | 0.18 | 0.02 | 0.63 | 0.08 |  | 0.09 | 0.02 | 0.23 | 0.07 | *0.42* |
| 65 | WW-TW | 0.17 | 0.01 | 0.48 | 0.04 |  | 0.05 | 0.01 | 0.12 | 0.02 | *0.42* |
| 66 | WW-VW | 0.98 | 0.08 | 2.09 | 0.17 |  | 0.03 | 0.01 | 0.18 | 0.07 | *0.42* |
| 67 | WW-WW | 1.40 | 0.40 | 3.09 | 0.89 |  | 0.10 | 0.02 | 1.10 | 0.39 | *0.21* |
| 68 | WW-YF | 1.14 | 0.24 | 2.42 | 0.51 |  | 0.07 | 0.02 | 0.45 | 0.06 | *0.42* |
| 69 | YF-IF | 0.44 | 0.04 | 1.18 | 0.12 |  | 0.25 | 0.09 | 0.50 | 0.16 | *0.42* |
| 70 | YF-KW | 0.03 | 0.01 | 0.19 | 0.04 |  | 0.13 | 0.03 | 0.46 | 0.14 | *0.42* |
| 71 | YF-LW | 0.60 | 0.03 | 1.50 | 0.07 |  | 0.18 | 0.03 | 0.62 | 0.08 | *0.42* |
| 72 | YF-QW | 0.02 | 0.00 | 0.06 | 0.01 |  | 0.15 | 0.03 | 0.29 | 0.05 | *0.42* |
| 73 | YF-RF | 0.05 | 0.01 | 0.28 | 0.04 |  | 0.17 | 0.03 | 0.78 | 0.20 | *0.42* |
| 74 | YF-RW | 0.06 | 0.02 | 0.39 | 0.12 |  | 0.14 | 0.05 | 0.71 | 0.11 | *0.42* |
| 75 | YF-SW | 0.15 | 0.02 | 0.43 | 0.06 |  | 0.13 | 0.02 | 0.51 | 0.13 | *0.42* |
| 76 | YF-TW | 0.20 | 0.03 | 0.67 | 0.09 |  | 0.15 | 0.03 | 0.51 | 0.06 | *0.42* |
| 77 | YF-VW | 0.43 | 0.01 | 1.07 | 0.02 |  | 0.10 | 0.03 | 0.33 | 0.12 | *0.42* |
| 78 | YF-YF | 0.44 | 0.09 | 1.50 | 0.31 |  | 0.20 | 0.03 | 0.25 | 0.04 | *0.21* |

# Peptide characterization data

**ph-IF**


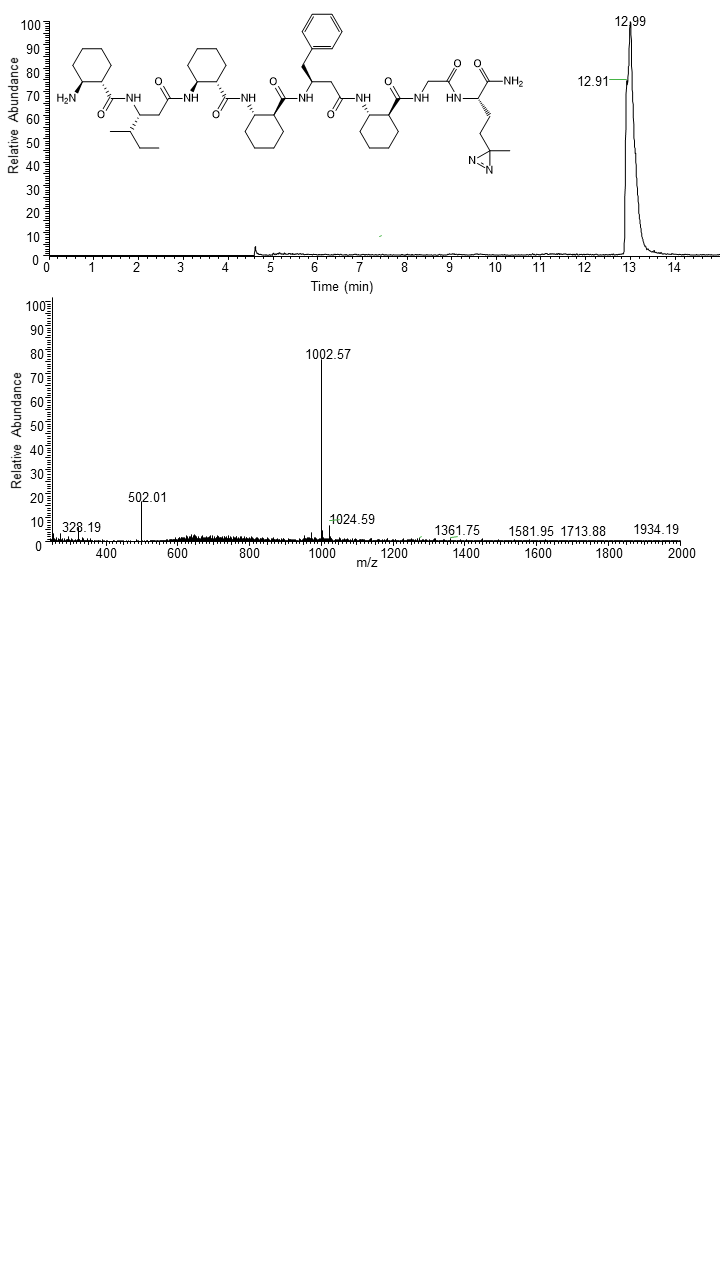


HPLC trace and mass spectrum of the **ph-IF** (Exact mass: 1001.64) Calculated m/z (average): 1002.65 [M+H]^+^, 501.83 [M+2H]^2+^; Observed m/z: 1002.57 [M+H]^+^, 502.01 [M+2H]^2+^

**ph-KW**

^
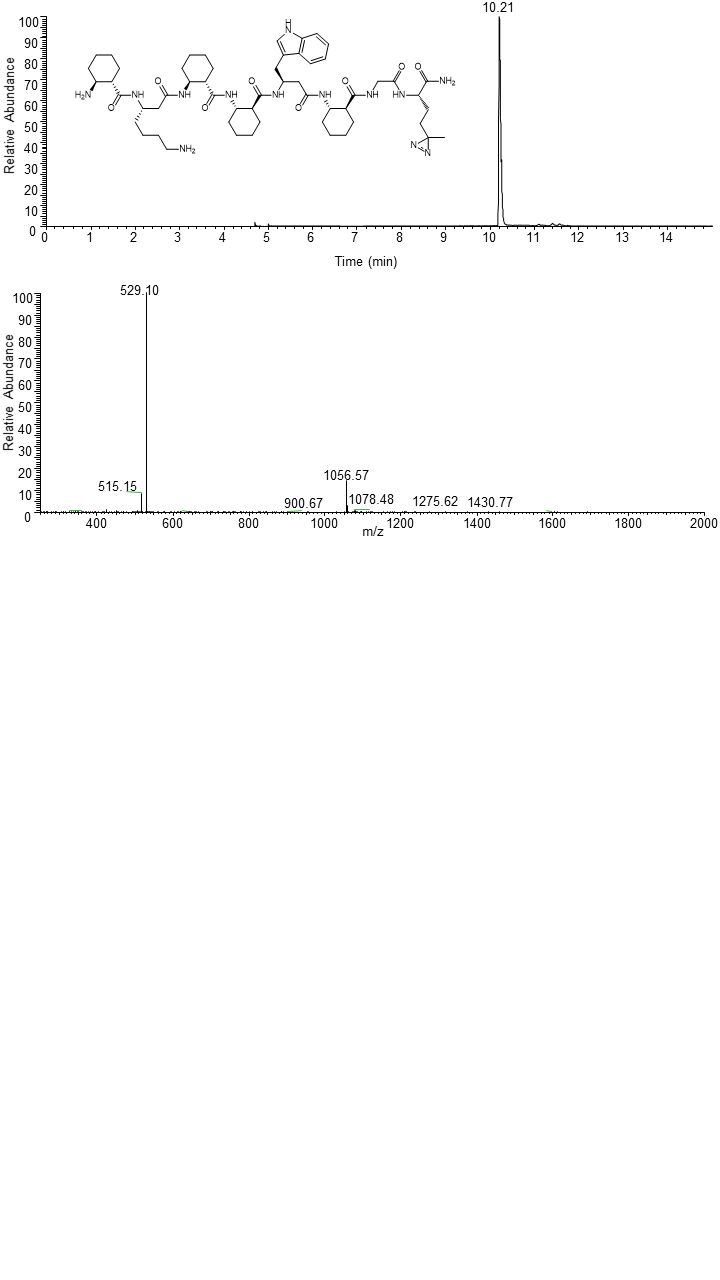
^

HPLC trace and mass spectrum of the **ph-KW** (Exact mass: 1055.66) Calculated m/z (average): 1056.67 [M+H]^+^, 528.84 [M+2H]^2+^; Observed m/z: 1056.57 [M+H]^+^, 529.1 [M+2H]^2+^

**ph-TW**


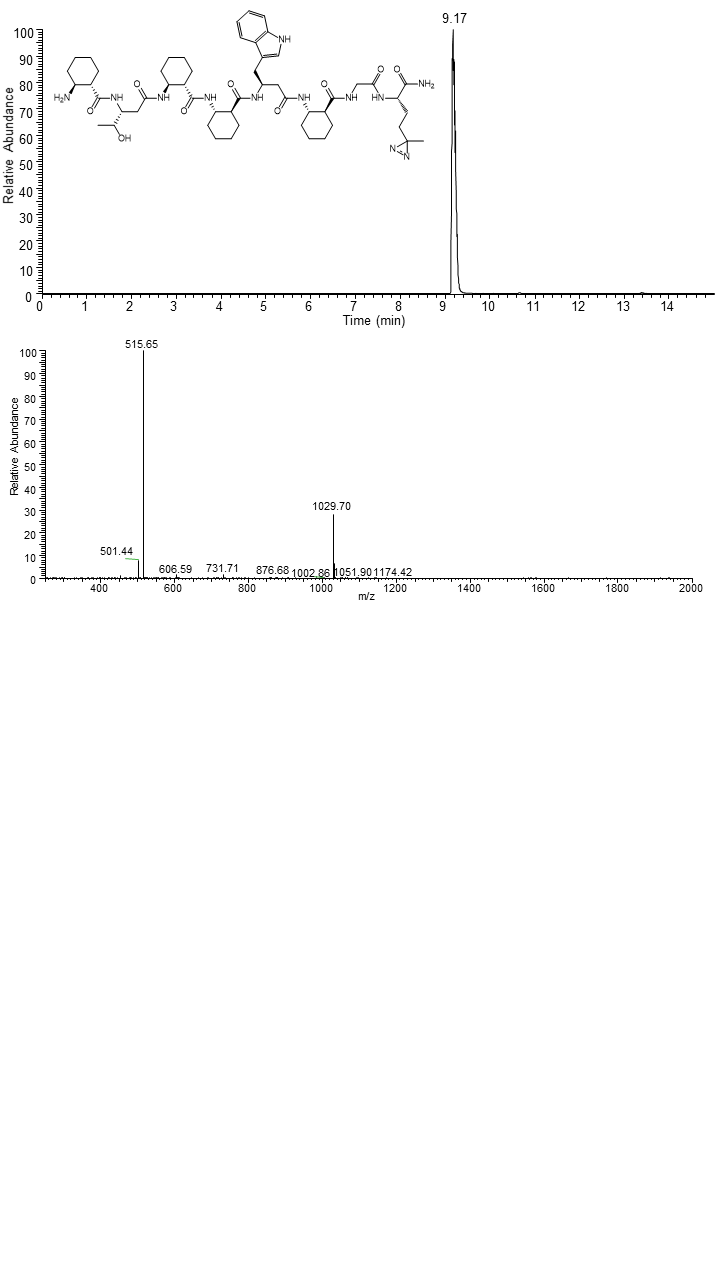


HPLC trace and mass spectrum of the **ph-TW** (Exact mass: 1028.62) Calculated m/z (average): 1029.63 [M+H]^+^, 515.32 [M+2H]^2+^; Observed m/z: 1029.70 [M+H]^+^, 515.65 [M+2H]^2+^.

**VW-G**

HPLC trace and mass spectrum of the Glutathione precursor **VW- SG** (exact mass: 1352.66); m/z calculated 1354.65 [M+H]^+^, 677.83 [M+2H]^2+^; m/z observed: 1353.67 [M+H]^+^, 677.66 [M+2H]^2+^.

**VF- G**

HPLC trace and mass spectrum of the glutathione precursor **VF-SG** (exact mass: 1313.65); m/z calculated 1315.65 [M+H]^+^, 658.33 [M+2H]^2+^; m/z observed: 1314.80 [M+H]^+^, 658.24 [M+2H]^2+^.

**QR-G**

**
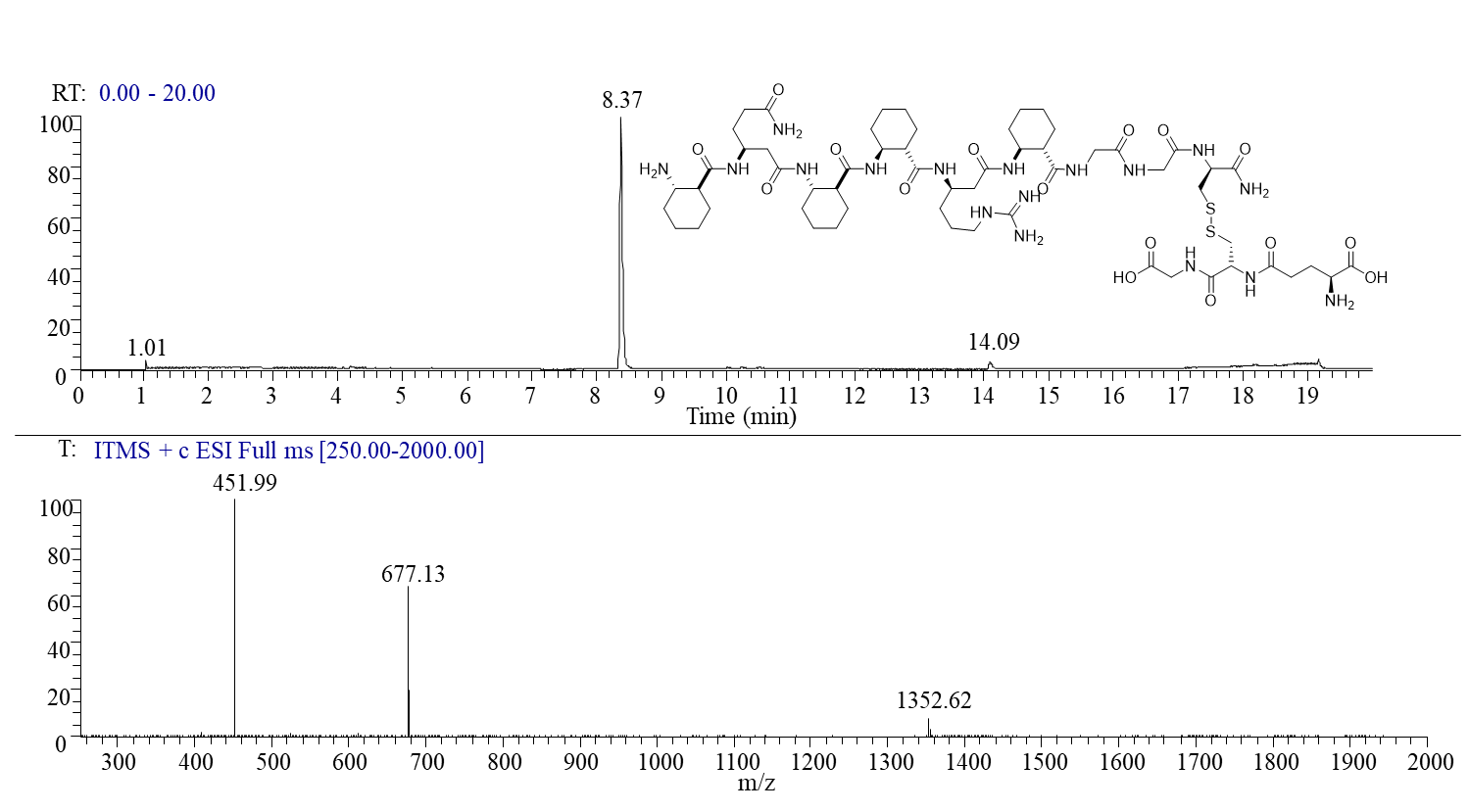
**

HPLC trace and mass spectrum of the glutathione precursor **QR-SG** (exact mass: 1351.67); m/z calculated 1353.75 [M+H]^+^, 677.38 [M+2H]^2+^, 451.92 [M+2H]^3+^; m/z observed: 1352.62 [M+H]^+^, 677.13 [M+2H]^2+^, 451.99 [M+2H]^3+^.

**LR-G**

**
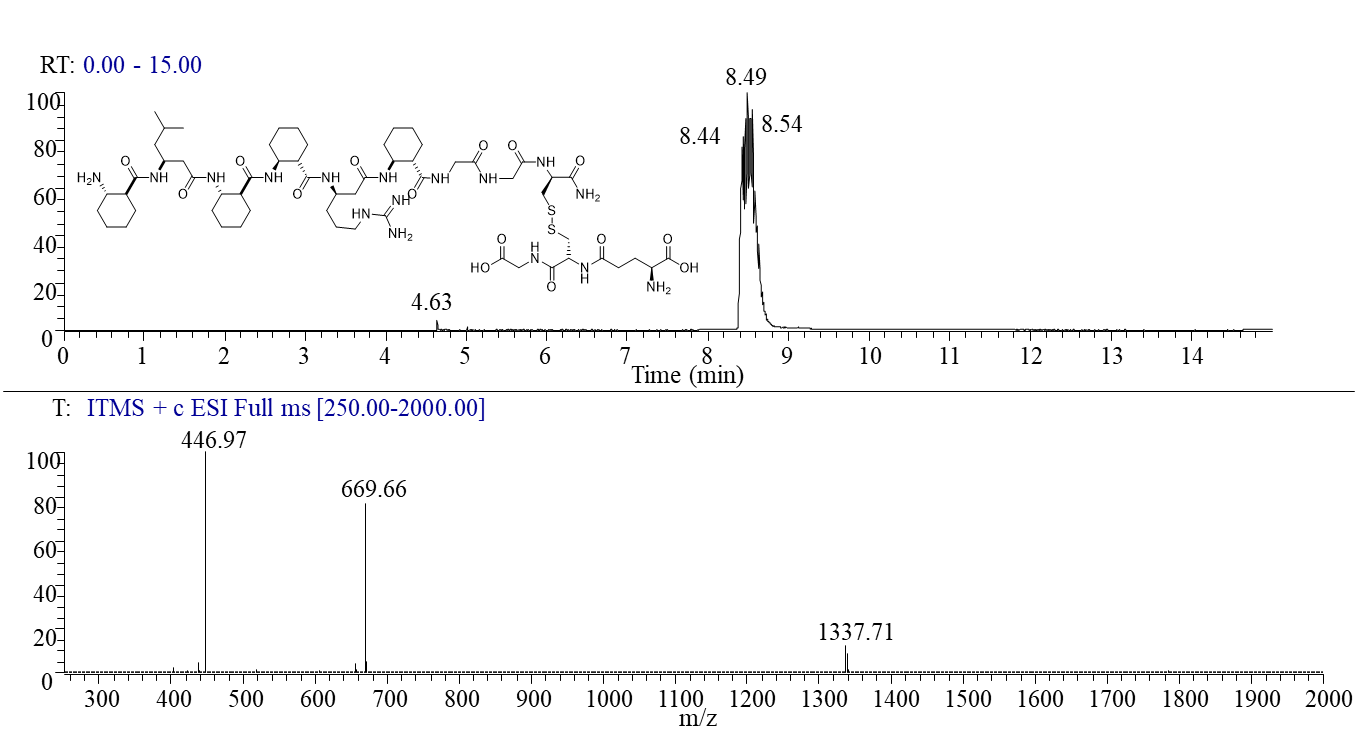
**

HPLC trace and mass spectrum of the glutathione precursor **LR-SG** (exact mass: 1336.70); m/z calculated 1338.75 [M+H]^+^, 669.88 [M+2H]^2+^, 446.92 [M+2H]^3+^; m/z observed: 1337.71 [M+H]^+^, 669.66 [M+2H]^2+^, 446.97 [M+2H]^3+^.

**QF-G**

**
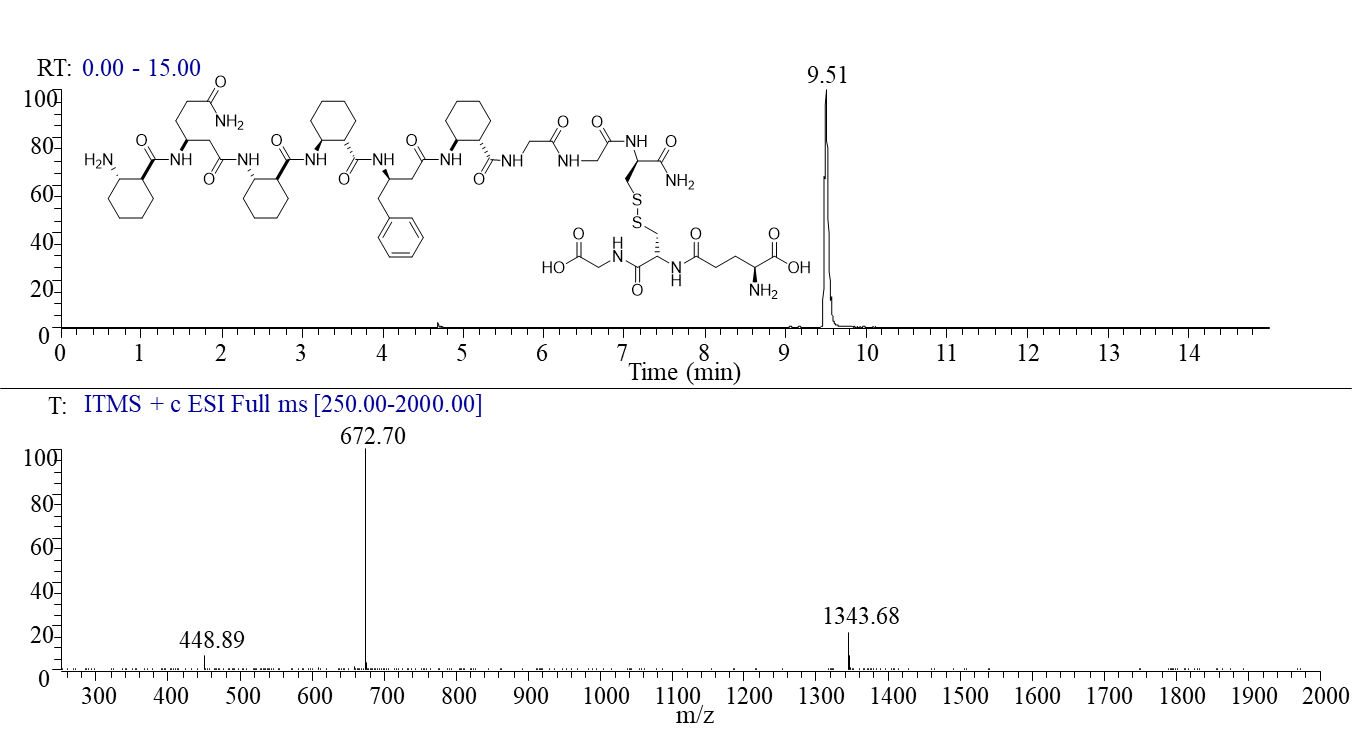
**

HPLC trace and mass spectrum of the glutathione precursor **QF-SG** (exact mass: 1342.64); m/z calculated 1344.75 [M+H]^+^, 672.88 [M+2H]^2+^, 448.92 [M+2H]^3+^; m/z observed: 1343.68 [M+H]^+^, 672.70 [M+2H]^2+^, 448.89 [M+2H]^3+^.

**WQ-G**

**
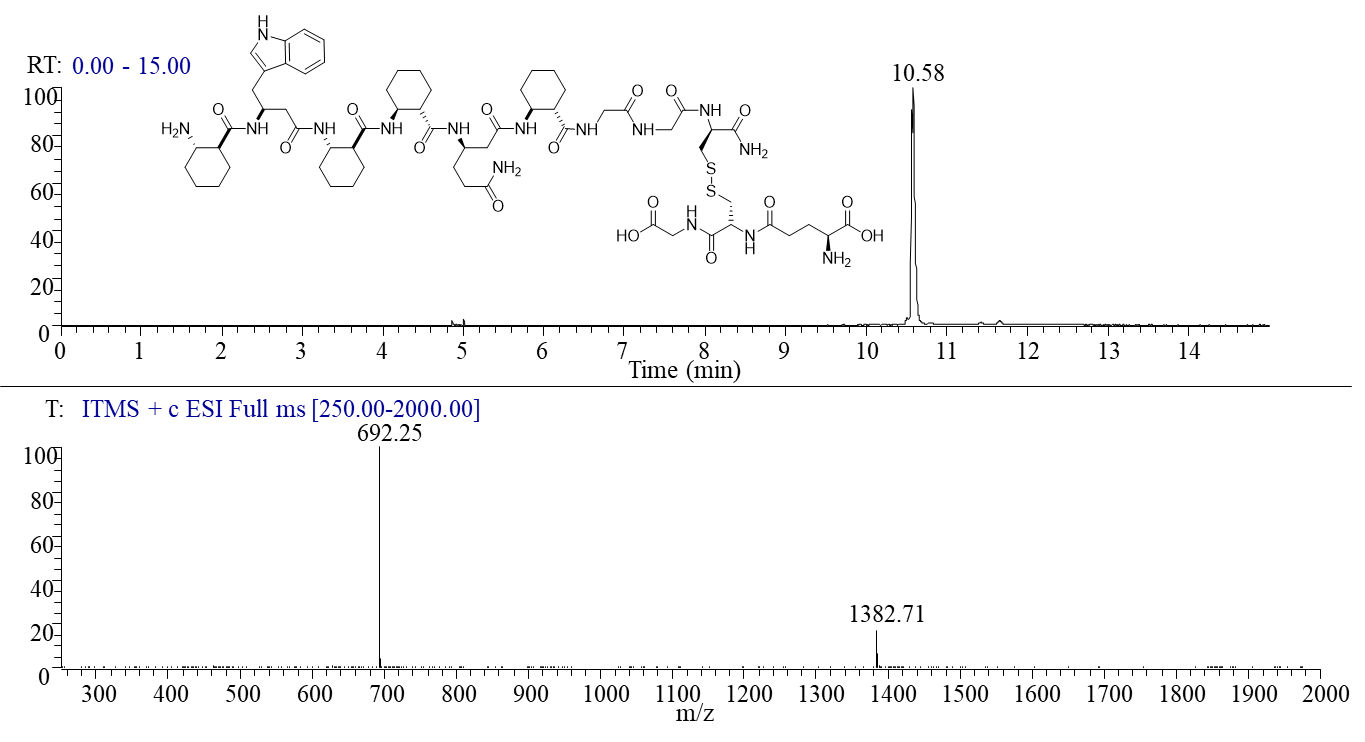
**

HPLC trace and mass spectrum of the glutathione precursor **WQ-SG** (exact mass: 1381.65); m/z calculated 1383.75 [M+H]^+^, 692.38 [M+2H]^2+^; m/z observed: 1382.71 [M+H]^+^, 692.25 [M+2H]^2+^.

**WR-G**


HPLC trace and mass spectrum of the glutathione precursor **WR-SG** (exact mass: 1409.69); m/z calculated 1411.75 [M+H]^+^, 706.38 [M+2H]^2+^, 471.25 [M+2H]^3+^; m/z observed: 1411.64 [M+H]^+^, 706.14 [M+2H]^2+^, 471.32 [M+2H]^3+^.

**QW-G**


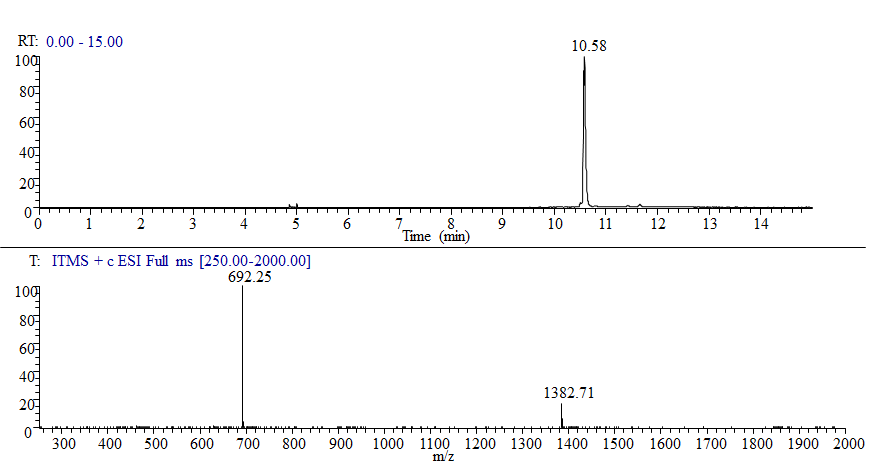


HPLC trace and mass spectrum of the glutathione precursor **QW-SG** (exact mass: 1381.65); m/z calculated 1383.75 [M+H]^+^, 692.38 [M+2H]^2+^; m/z observed: 1382.71 [M+H]^+^, 692.25 [M+2H]^2+^.

Supporting references

Author Contributions

É.B. synthesized the foldameric building blocks, performed the dissipative chemical network experiments with CaM and analysed the data. E.W. performed the photofoldamer screening studies and the ITC experiments and evaluated the data. A.T. carried out the CaM protein expression and analysed the protein-foldamer interactions. F.B. fitted the kinetic chemical evolution model to the experimental data arrays, M.R.F.M. performed the UV-induced disulfide rearrangement study in the presence of K-Ras G12D and synthesized the foldameric building blocks. G.K. Z. Sz. and Z.K. carried out CaM stability test and binding site localization by mass spectrometry. A.P. and M.G. carried out the K-Ras G12D protein expression and purification. Z. O. and G. M. K. designed the photofoldamer screening studies. T.A.M designed the experiment, evaluated the experimental results and wrote the manuscript.

References

[1] É. Bartus, A. Tököli, B. Mag, Á. Bajcsi, G. Kecskeméti, E. Wéber, Z. Kele, G. Fenteany, T. A. Martinek, “Light-Fueled Primitive Replication and Selection in Biomimetic Chemical Systems” *J. Am. Chem. Soc.* **2023**, *145*, 13371–13383.

[2] E. Wéber, P. Ábrányi‐Balogh, B. Nagymihály, D. K. Menyhárd, N. Péczka, M. Gadanecz, G. Schlosser, Z. Orgován, F. Bogár, D. Bajusz, G. Kecskeméti, Z. Szabó, É. Bartus, A. Tököli, G. K. Tóth, T. V. Szalai, T. Takács, E. De Araujo, L. Buday, A. Perczel, T. A. Martinek, G. M. Keserű, “Target‐Templated Construction of Functional Proteomimetics Using Photo‐Foldamer Libraries” *Angew. Chem. Int. Ed.* **2024**, e202410435.

[3] P. Virtanen, R. Gommers, T. E. Oliphant, M. Haberland, T. Reddy, D. Cournapeau, E. Burovski, P. Peterson, W. Weckesser, J. Bright, S. J. Van Der Walt, M. Brett, J. Wilson, K. J. Millman, N. Mayorov, A. R. J. Nelson, E. Jones, R. Kern, E. Larson, C. J. Carey, İ. Polat, Y. Feng, E. W. Moore, J. VanderPlas, D. Laxalde, J. Perktold, R. Cimrman, I. Henriksen, E. A. Quintero, C. R. Harris, A. M. Archibald, A. H. Ribeiro, F. Pedregosa, P. Van Mulbregt, SciPy 1.0 Contributors, A. Vijaykumar, A. P. Bardelli, A. Rothberg, A. Hilboll, A. Kloeckner, A. Scopatz, A. Lee, A. Rokem, C. N. Woods, C. Fulton, C. Masson, C. Häggström, C. Fitzgerald, D. A. Nicholson, D. R. Hagen, D. V. Pasechnik, E. Olivetti, E. Martin, E. Wieser, F. Silva, F. Lenders, F. Wilhelm, G. Young, G. A. Price, G.-L. Ingold, G. E. Allen, G. R. Lee, H. Audren, I. Probst, J. P. Dietrich, J. Silterra, J. T. Webber, J. Slavič, J. Nothman, J. Buchner, J. Kulick, J. L. Schönberger, J. V. De Miranda Cardoso, J. Reimer, J. Harrington, J. L. C. Rodríguez, J. Nunez-Iglesias, J. Kuczynski, K. Tritz, M. Thoma, M. Newville, M. Kümmerer, M. Bolingbroke, M. Tartre, M. Pak, N. J. Smith, N. Nowaczyk, N. Shebanov, O. Pavlyk, P. A. Brodtkorb, P. Lee, R. T. McGibbon, R. Feldbauer, S. Lewis, S. Tygier, S. Sievert, S. Vigna, S. Peterson, S. More, T. Pudlik, T. Oshima, T. J. Pingel, T. P. Robitaille, T. Spura, T. R. Jones, T. Cera, T. Leslie, T. Zito, T. Krauss, U. Upadhyay, Y. O. Halchenko, Y. Vázquez-Baeza, “SciPy 1.0: fundamental algorithms for scientific computing in Python” *Nat. Methods* **2020**, *17*, 261–272.

[4] H. Motulsky, A. Christopoulos, *Fitting Models to Biological Data Using Linear and Nonlinear Regression: A practical guide to curve fitting*, Oxford University PressNew York, NY, **2004**.

[5] A. C. Hindmarsh, L. R. Petzold, “LSODAR, ordinary differential equation solver for stiff or non-stiff system with root-finding” **2005**.

[6] S.-Y. Lau, E. Procko, R. Gaudet, “Distinct properties of Ca2+–calmodulin binding to N- and C-terminal regulatory regions of the TRPV1 channel” *J. Gen. Physiol.* **2012**, *140*, 541–555.

[7] M. T. Marty, A. J. Baldwin, E. G. Marklund, G. K. A. Hochberg, J. L. P. Benesch, C. V. Robinson, “Bayesian Deconvolution of Mass and Ion Mobility Spectra: From Binary Interactions to Polydisperse Ensembles” *Anal. Chem.* **2015**, *87*, 4370–4376.

[8] É. Bartus, Z. Hegedüs, E. Wéber, B. Csipak, G. Szakonyi, T. A. Martinek, “De Novo Modular Development of a Foldameric Protein–Protein Interaction Inhibitor for Separate Hot Spots: A Dynamic Covalent Assembly Approach” *ChemistryOpen* **2017**, *6*, 236–241.

1. The separate initiation ([1a] and [2a]), termination ([1b] and [2b]), and chain propagation steps ([3a,b] and [4a,b]) are indicated with the back-and-forth arrows which do not refer to any preequilibrium or microscopic reversibility. [↑](#footnote-ref-2)
